# Nutrition Metabolism Plays an Important Role in the Alternate Bearing of the Olive Tree (*Olea europaea* L.)

**DOI:** 10.1371/journal.pone.0059876

**Published:** 2013-03-28

**Authors:** Mine Turktas, Behcet Inal, Sezer Okay, Emine Gulden Erkilic, Ekrem Dundar, Pilar Hernandez, Gabriel Dorado, Turgay Unver

**Affiliations:** 1 Faculty of Science, Department of Biology, Cankiri Karatekin University, Cankiri, Turkey; 2 Department of Biology, Faculty of Art and Science, Balikesir University, Balikesir, Turkey; 3 Instituto de Agricultura Sostenible (IAS-CSIC), Alameda del Obispo s/n, Córdoba, Spain; 4 Dep. Bioquímica y Biología Molecular, Campus Rabanales C6-1-E17, Campus de Excelencia Internacional Agroalimentario (ceiA3), Universidad de Córdoba, Córdoba, Spain; East Carolina University, United States of America

## Abstract

The olive tree (*Olea europaea* L.) is widely known for its strong tendency for alternate bearing, which severely affects the fruit yield from year to year. Microarray based gene expression analysis using RNA from olive samples (on-off years leaves and ripe-unripe fruits) are particularly useful to understand the molecular mechanisms influencing the periodicity in the olive tree. Thus, we carried out genome wide transcriptome analyses involving different organs and temporal stages of the olive tree using the NimbleGen Array containing 136,628 oligonucleotide probe sets. Cluster analyses of the genes showed that cDNAs originated from different organs could be sorted into separate groups. The nutritional control had a particularly remarkable impact on the alternate bearing of olive, as shown by the differential expression of transcripts under different temporal phases and organs. Additionally, hormonal control and flowering processes also played important roles in this phenomenon. Our analyses provide further insights into the transcript changes between ”on year” and “off year” leaves along with the changes from unrpipe to ripe fruits, which shed light on the molecular mechanisms underlying the olive tree alternate bearing. These findings have important implications for the breeding and agriculture of the olive tree and other crops showing periodicity. To our knowledge, this is the first study reporting the development and use of an olive array to document the gene expression profiling associated with the alternate bearing in olive tree.

## Introduction

The olive (*Olea europaea* L., Oleaceae) is an evergreen tree that is largely distributed in the Mediterranean Basin. Its cultivated forms have been introduced into many areas [1], being one of the most economically important fruit crops in the world from a socio-economical point of view, and also due to the nutritional properties of the olive fruits and the olive oil derived from them. In fact, the olive oil has gained the label of “qualified health claim” for cardiovascular protection by the internationally recognized Food and Drug Administration (FDA) of the United States of America (USA) <http://www.fda.gov/Food/LabelingNutrition/LabelClaims/QualifiedHealthClaims/default.htm>. Such claim considers the olive oil as a healthy medicine, due to its protective effect against cardiovascular diseases, being the third of such labels approved for a conventional food (after the hazelnut and omega-3 fatty acids) <http://www.fda.gov/Food/LabelingNutrition/LabelClaims/QualifiedHealthClaims/ucm072756.htm>. The olive tree is adapted to abiotic stresses like drought and heat (Mediterranean climate), and thus the climate change and global warming. Additionally, the olive tree fields are important from an ecological point of view, being a source of biodiversity.

The olive tree exhibits a peculiar behavior, named alternate bearing (biennial bearing or periodicity), being defined as a tendency of some fruit trees not to bear a regular and similar crop year after year. Thus, a high-yield crop year (“on-year”) is followed by a low-yield or even a no-crop year (“off-year“), which severely affects the fruit yield [2]. Since this phenomenon occurs in different types of fruit trees, it has been suggested that all species showing alternating may behave in a similar unified manner [3]. The alternate bearing is so pronounced in the olive tree, that it has been considered that this crop shows a biennial developmental cycle.

The alternate bearing represents a strategic mechanism of the olive tree to save nutrient reserves for significant vegetative growth, as well as to survive biotic and abiotic stresses in environments prone to macronutrient/micronutrient deficiencies in a dry climate, like the one of the Mediterranean Basin. Yet, the periodicity in bearing of the olive tree represents a serious problem from the breeding and agricultural points of view, since the fruit yield may not be regular and uniform year-over-year, but instead may suffer extremely drastic variations, from a high-production to even no-yield at all.

The olive tree produces vegetative buds that generate shoots and leaves after the seed germination for 12 o more years in normal field conditions (juvenile period). Once the adult (reproductive) period is induced by natural or artificial conditions (which may reduce the juvenile period to just two years after germination), the olive tree buds undergo a standard differentiation program towards flowers and fruits (with a natural tendency to produce large numbers of them), being genetically controlled [4], unless such pathway is inhibited. In such a case, the olive tree buds are directed towards vegetative buds. Interestingly, the developing seeds inside the olive fruits produce molecular messengers (eg., gibberellins) that are effective inhibitors of the floral induction. Such floral bud inhibition may also occur when the olive tree carbohydrate reserves are scarce, which is typical after a high-yield fruit production, as well as due to abiotic and biotic stresses that may deplete such reserves.

Although many genetic and physiological traits of species affect the yield variation between “on” and “off” years, three main factors have been suggested for the alternate bearing in fruit trees [5]. They include i) flowering-site limitation, with the competition between vegetative and reproductive organs being proposed to have influence on the periodicity in the olive tree [3]; ii) nutritional control, since it has been shown that the storage of nutrients during the “off” year is used for reproductive growth the following year in some species like the pistachio tree [6]; and iii) endogenous hormonal control, since differences in certain endogenous hormones in the olive tree have been reported, with balances between these hormones being considered as key regulators of the alternate bearing [7].

These facts have led to different agronomical strategies to limit or even eliminate the periodicity in bearing in the olive tree; namely: i) pruning the year before the expected high production, effectively reducing the subsequent fruit yield; ii) reduction of the high-density of the tiny olive fruits at the earliest possible developmental stage, by physical fruit excision; iii) early harvesting of the immature olive fruits (large but still green; before they become mature, which typically are purple, black, brown or pink, depending on the variety), which may help to reduce the alternate bearing severity in some cases, even though at such stage the flowering inhibition has already started; and iv) favoring the biosynthesis and accumulation of carbohydrate reserves in the olive tree, providing a proper nourishment (light, micronutrients/macronutrients, irrigation, etc) [8].

The induction-initiation cycle of olive tree takes about eight months. It starts in July, while the floral initiation occurs in November and the process is completed in March [9]. As indicated, the olive tree is well known for its extreme alternation, with considerable effect on crop yield. Due to this tendency, difference between “on” and “off” year product yield varies between 5–30 t/ha [2]. This is therefore a crucial phenomenom to consider for its cultivation management. For example, recent studies [10], [11] have shown that crop loads influence irrigation response, in a complex process where the degree of water deficit and the age of the orchad are interactive factors [12].

Dag et al. [13] showed that the main factor determining flowering and fruit yield in the olive tree was the existence of new mature buds. Since the transition from the vegetative to the reproductive phase is under the tight control of a complex genetic network [14], discovering control mechanisms of these transitions is crucial to understand the basis of this tendency. Ozdemir-Ozgenturk et al. [15] constructed cDNA libraries from young olive tree leaves and immature fruits, and arbitrarily sequenced 3,734 ESTs to identify the functions of the genes, and annotated them by homologies to known genes. In order to identify microRNA (miRNA) associated to such phase-transition in the olive tree, Donaire et al. [16] sequenced miRNA from the juvenile and adult shoots. They identified several miRNA, and suggested that miR156, miR172 and miR390 were involved in controlling the developmental transition. On the other hand, Fernández-Ocaña et al, 2010 have used subtractive cDNA libraries to identify a differentially expressed gene (*jat*) involved in the juvenile-to-adult transition of the olive tree.

On the other hand, the microarray analysis for genome-wide transcription analysis is a powerful approach to reveal the changes in the gene expression profiles of organisms in response to different conditions, and thus provides wide-scale insights into the underlying molecular mechanisms. In fact, the transcriptome profiling has been widely used to uncover regulatory processes in several plant species [17]–[23]. Microarray hybridization allows the use of closely-related non target species probe sets, thus paving the way for unsequenced genomes like the olive to be analyzed.

In the present study, the microarray expression profiling of six *O. europaea* samples from on-off years and ripe-unripe fruits was performed to facilitate the understanding of the molecular basis of the alternate bearing in the olive tree. A total of 136,628 oligonucleotide probe sets, based on the publicly available olive ESTs as well as the sequenced model species populus were arrayed by using a comparative genomics approach. The gene expression profiles with regard to different tissues and temporal stages were examined. The results presented will greatly help unravel the molecular network involved in the olive periodicity in addition to providing useful information for the olive breeding programs with a corresponding impact on olive agriculture in general.

## Materials and Methods

### Plant Material

Leaves of two side by side olive (*Olea europaea* cv. Ayvalık) trees (15 years old, about 5 m high, and approximately 4 m apart from each other) were collected from the Edremit Olive Seedling Growing Station (Balikesir Province, Turkey. All necessary permits were obtained for the described field studies). Six sample sets were prepared: i) unripe fruit (UF); ii) ripe fruit (RF); iii) “on-year” mature leaf (November sample, ON-M); iv) “on-year” juvenile leaf (July sample, ON-J); v) “off-year” mature leaf (November samples, OFF-M); and vi) “off-year” juvenile leaf (July sample, OFF-J). The fruited (on year) leaves were collected in July (juvenile) and November (mature) 2010, while the non-fruited (off year) leaves were taken in the same period of 2011. The unripe and ripe fruits were collected on July and October 2011 from an ‘on’ tree. After collection, the samples were directly transferred into liquid nitrogen and stored at –80°C until used. The “on year” and the “off year” olive trees were approximately 4 m apart from each other, and they were not shading one another.

### RNA Isolation, cDNA Synthesis, Labeling and Hybridization

For each sampling data, the total RNA was extracted using the RNeasy Plant Mini Kit (Qiagen, Hilden, Germany) according to the manufacturer’s instructions. The RNA quality was checked on 1.5% agarose gel, and the concentration of the RNA was determined using a NanoDrop 2000c spectrophotometer (Thermo Fisher Scientific, Lenexa, KS, USA). Two biological replicates of each six sample were used in the analysis. Double stranded cDNA was synthesized from 10 µg of total RNA using the SuperScript Double-Stranded cDNA Synthesis Kit (Invitrogen, Carlsbad, CA, USA) and labeled with Cy3 random nonamers with the One-Color DNA Labeling Kit (Roche NimbleGen, Madison, WI, USA). The following steps were carried out with the equipment and software from the same manufacturer. Thus, the transcriptome profiles of the samples were analyzed by direct comparison of the transcription activities between the six olive tree data sets on the same oligo microarray. The custom 12×135K array was incubated at 42°C for 17 h in a Hybridization System 4, and washed at room temperature following the manufacturer’s directions. Then, the microarray slide was scanned with 2 µm resolution using a MS 200 Microarray Scanner, generating the corresponding 532 nm TIFF images. The data were imported into the DEVA software to quantify the signal intensities of the spots on the image.

### Microarray Design

The microarrays were designed according to the Roche NimbleGen protocol. A large-scale custom microarray comprising a total of 136,628 oligonucleotide probes was designed for broad representation of the olive tree transcriptome. Due to the scarcity of olive tree Expressed Sequence Tag (EST) sequences in databases, we have conducted cross-species microarray hybridizations using oligoarrays derived from the closest-available related species (*Populus trichocarpa*). Thus, 16,629 probes were based on olive tree EST sequences, while the rest were from *P. trichocarpa* cDNA sequences. In consequence, some ESTs were non-annotated. The ESTs were spotted as duplicates and triplicates on the array. The array comprised 5,543 and 49,072 individual EST derived from the olive tree and poplar, respectively.

### Data Processing and Statistical Analyses

The normalization of the signal intensities was carried out with the DEVA software previously indicated. The signal intensities of the samples were transformed into log_2_-ratio data. The array data were normalized according to the quantile method for standardization [24], and the Robust Multichip Average (RMA) algorithm [25]. The dye-normalized and background-subtracted intensity data were exported into the ArrayStar software (DNAStar, Madison, WI, USA) to perform gene expression analyses. The Student’s *t-*test was used to identify differentially expressed genes. A gene was defined as being differentially expressed only if the log_2_-based expression value of the gene differed more than two-fold and *P*<0.05 between two data sets.

A single-raw intensity value was determined for each gene in the array by averaging two or three spot replicates of each gene. Out of 55,504 individual genes, 54,515 produced appropriate signals and thus were used for the further statistical analyses. The Basic Local Alignment Search Tool (BLAST) algorithm and BLAST to Gene Ontology (Blast2GO) tool was used against the National Center for Biotechnology Information (NCBI) database to annotate the genes corresponding to the hybridized cDNA signals. The Kyoto Encyclopedia of Genes and Genomes (KEGG) pathway analyses were performed for the predicted target genes, to improve the elucidation of the biological functions of the genes. Putative mRNA sequences were used as queries against the KEGG database.

The data produced in this work have been deposited in NCBI’s Gene Expression Omnibus and are accessible through GEO Series accession number GSE42950.

### Quantitative RT-PCR

To verify the microarray data, the expressions of nine selected genes were measured via quantitative Reverse-Transcription Polymerase Chain Reaction (qRT-PCR). The relative expression levels of the predicted olive tree genes were compared in the UF, RF, ON-M, OFF-M, ON-J and OFF-J samples. The expression profiles of these genes were also measured and the specific PCR primers used in the qRT-PCR are listed in Table 1. The reverse transcription reaction was performed with the Fermentas First Strand cDNA Synthesis Kit (Thermo Fisher Scientific) according to the manufacturer’s protocol. The qRT-PCR experiments were carried out as previously reported [26]. Briefly, 2 µl of cDNA were amplified with 0.1 µl of specific primers in a total volume of 18 µl, using a LightCycler 480 Real-Time PCR System with SYBR Green I Master (Roche Applied Science, Penzberg, Germany). Specific PCR primers were designed using the Primer3Plus software version 2.3.3<http://primer3plus.com> [27]. The 18S rRNA (GenBank ID: AF147501; forward primer: 5′-GTGACGGGTGACGGAGAATT-3′; reverse primer: 5′-GACACTAATGCGCCCGGTAT-3′) was used as the normalizer RNA/cDNA/gene [28], [29]. The qRT-PCR conditions were as follows: preheating at 95°C for 10 min; and 40 cycles (95°C for 30 s; 52°C, 54°C or 57°C, depending on the cDNA annealing temperature, for 1 min; and 72°C for 10 min).

**Table 1:** Sequences of the primers used for qRT-PCR.

Seq_ID	GenBank Accession	Description	Forward Primer (5′ –>3′)	Reverse Primers (5′ –>3′)
FL684126	gi|256857013	Hypothetical protein	ATGTGCCGGTTCTCTTTCAG	GTTCACCGTTGAGGAGGTTC
GO243710	gi|242396505	Hypothetical protein	GGCAAGATTGACTCACAGCA	GAAGCCCTTTTCGAGGATTT
GO244999	gi|242394448	Hypothetical protein	CACTTTTCAGCCACAGGTCA	CCTCTGGCATTGGTTTCACT
GO245913	gi|242392926	Hypothetical protein	GTGGTGTTGGTGATGGGACT	ATGCTTCCGATTTTTGCATC
GO245304	gi|242394753	Hypothetical protein	AAAGGGGATGCCTCCATTAC	CATGTGCGGACACTATCAGG
GO244140	gi|242396935	Hypothetical protein	GCCTACCAGAGAACAACTTC	ATCGTCCACGTGTTTTAGGC
FL684126	gi|256857013	Hypothetical protein	ATGTGCCGGTTCTCTTTCAG	GTTCACCGTTGAGGAGGTTC
GO243710	gi|242396505	Hypothetical protein	GGCAAGATTGACTCACAGCA	GAAGCCCTTTTCGAGGATTT
GO244999	gi|242394448	Hypothetical protein	CACTTTTCAGCCACAGGTCA	CCTCTGGCATTGGTTTCACT
FL684399	gi|256857286	Hypothetical protein	CCATGCCACCAACTTCTTTT	AGCCAATAATGCGAGTGGTC

Three replicates were carried out for each sample. The gene expression levels were calculated as the mean-signal intensity across the three replicates. The normalizations were performed using the 18S rRNA.

## Results

To identify the genes involved in the alternate bearing phenomenon, the transcript expression values were analyzed on the six olive tree sample sets. The leaves and fruits were compared on the basis of their developmental stages (mature and juvenile), and the leaves were also evaluated by means of their timing (“on” and “off” years). The data obtained from the microarray analyses were arranged in five comparisons: i) unripe (UF) vs. ripe (RF) fruit; ii) “on-year” leaves of juvenile (ON-J) vs. mature (ON-M) stage; iii) “off-year” leaves of juvenile (OFF-J) vs. mature (OFF-M); iv) mature leaves of “on-year (ON-M) vs. “off-year” (OFF-M); and v) juvenile leaves of “on-year” (ON-J) vs. “off-year” (OFF-J).

### Accuracy of Microarray Analyses

**Figure 1:**
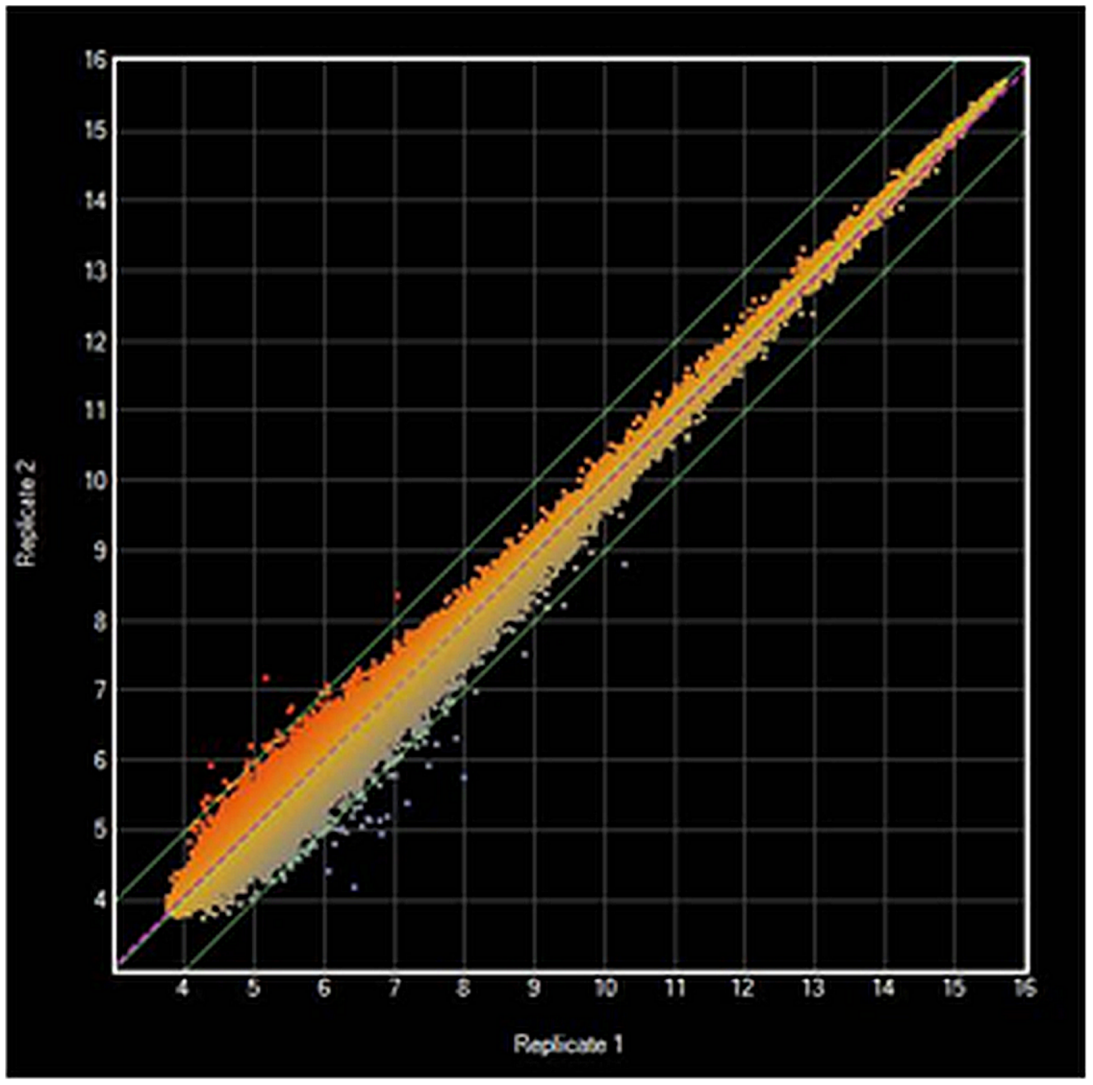
Scatter plot of replicates. A high correlation was found between the replicates (R^2^∶0.99).

A total of 55,504 individual probes were designed, of which 54,515 produced detectable signals (Table S1). The intensities of the hybridization signals were used to determine the target concentrations. The hybridization of each sample was replicated to ascertain the quality of each probe in the array. The signals of the probes and the replicates of the whole data sets were analyzed to examine the accuracy of the hybridizations. The Pearson’s coefficient correlation between the replicated data sets showed almost a perfect correlation (R^2^∶ 0.99), indicating a very-high reproducibility of the microarray experiment (Figure 1).

**Figure 2:**
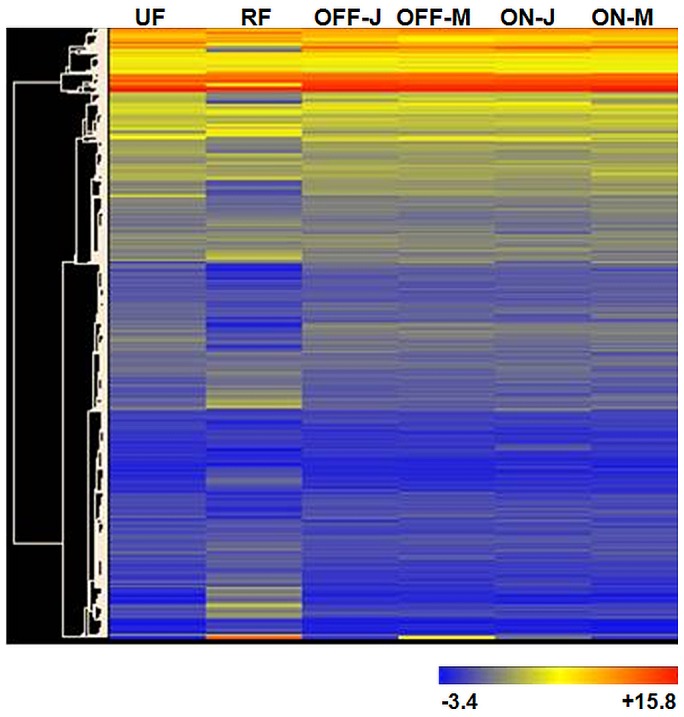
Heat map representing normalized signal intensities of 54,515 genes. Red indicates high, and blue indicates low gene expression.

The cluster analyses of the genes showed that samples collected from different organs could be sorted into different groups. Both the unripe and the ripe fruit samples fell into separate groups than the leaves, while the ripe fruit was placed far away from the rest. The analyses also indicated that 54,515 genes could be sorted into two main groups on the basis of their expression levels (Figure 2). The first group contained highly-expressed genes. The second group was divided into two subgroups; namely, the genes expressed at low levels, and the ones with intermediate expression levels.

**Figure 3:**
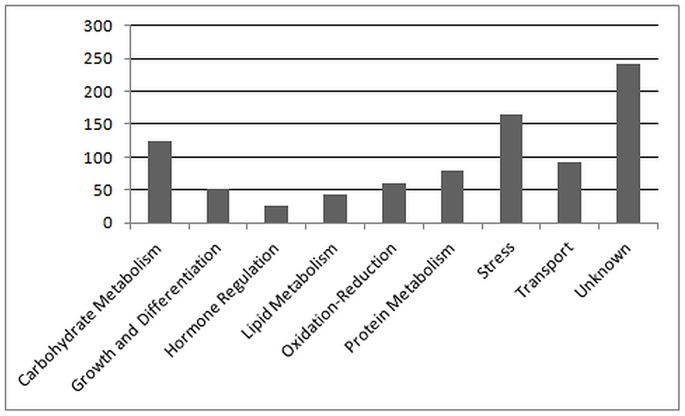
Differentially expressed transcripts between leaves comparisons. Transcripts are grouped on the basis of their predicted biological roles.

**Figure 4:**
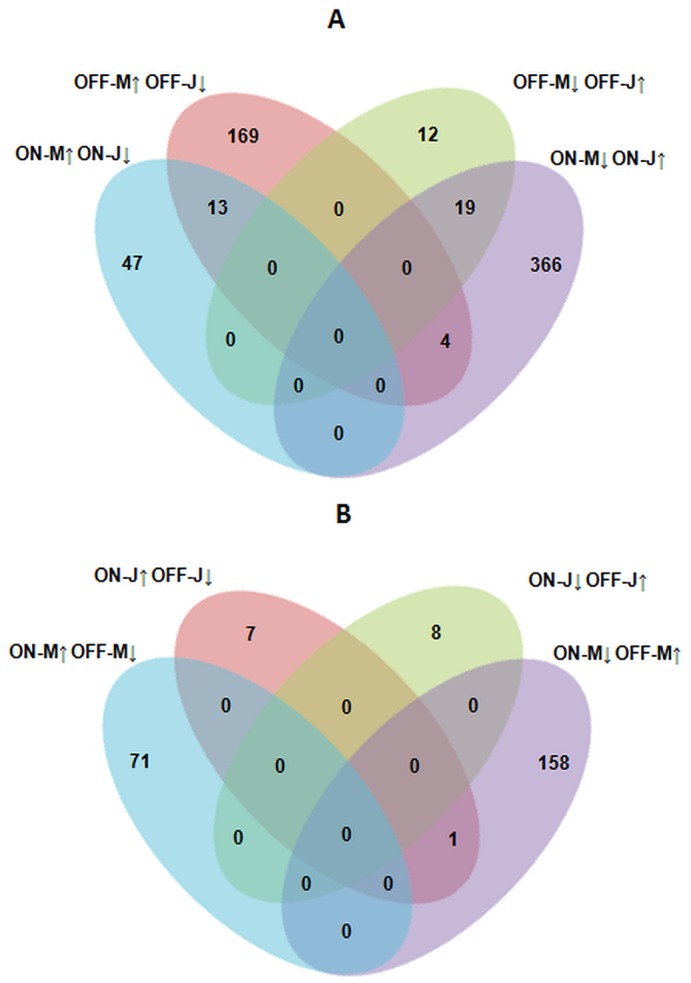
Genes differentially expressed according to the developmental stage and bearing year of leaves. Venn diagrams showing differentially expressed genes in the microarray experiments. Comparisons between juvenile and mature, and “on” and “off” years are shown in the panel A and B, respectively. Arrowhead orientation indicates up (↑) and down (↓) regulation.

The analyses revealed that the expression of 699 transcripts were altered among leaves. They were involved in different biological processes. Most of them were grouped into eight biological processes (Figure 3). Of those, a wide set were involved in unknown biological processes, and the rest were mainly related to carbohydrate metabolism, stress response, transport, oxidoreductase (redox) activity, growth, lipid metabolism and hormone regulation. The comparison between the olive tree samples showed that 630 transcripts were differentially expressed between mature and juvenile leaves, and 245 transcripts were differentially expressed between “on” and “off” years (Figure 4).

### Transcriptome Analysis of Ripe versus Unripe Fruits

A comparison between fruits identified 13,898 genes with statistically significant expression differences between samples (*P*<0.05). A wide set of them were related to photosynthesis, carbohydrate metabolism processes, ion transport and homeostasis, cell growth and differentiation, cytoskeleton organization, stress response, fatty-acid metabolism, hormone-mediated signaling and biosynthesis, transcription, protein modifications, rRNA processing, and transport. A total of 1,058 genes showed higher expressions in the ripe than in unripe fruits. Among the differentially expressed transcripts, 2,648 genes showed more than two-fold change between the fruits, with 15 genes showing eight-fold differences (Table 2).

**Table 2:** Transcripts showing more than eight-fold differential gene expression between unripe and ripe fruits. Fold changes were given in log_2_-based numbers.

Seq_ID	Description	UF-RF-fold change*	*P* value	GO biological process
FL683634_1	FL683634 D_L16_F08_0414F_p12 *Olea europaea* cv. Leccino fruitlet *Olea europaea* cDNA	–9.53	0	Unknown
FL683787_1	FL683787 D_E21_C11_0414F_p13 *Olea europaea* cv. Leccino fruitlet *Olea europaea* cDNA	–9.41	0	Unknown
FL683591_1	FL683591 D_A06_A03_0414F_p14 *Olea europaea* cv. Leccino fruitlet *Olea europaea* cDNA	–9.26	0	Unknown
FL683506_1	FL683506 D_K02_F01_0414F_p14 *Olea europaea* cv. Leccino fruitlet *Olea europaea* cDNA	–9.08	0	Unknown
FL683529_1	FL683529 D_B20_A10_0414F_p12 *Olea europaea* cv. Leccino fruitlet *Olea europaea* cDNA	–8.97	0	Anthocyanin biosynthetic process, regulation of flavonol biosynthetic process, response to oxidative stress, response to sucrose stimulus, response to UV-B, toxin catabolic process
FL684044_1	FL684044 D_G09_D05_0414F_p13 *Olea europaea* cv. Leccino fruitlet *Olea europaea* cDNA	–8.86	0	Response to oxidative stress
FL684399_1	FL684399 D_I08_E04_0414F_p14 *Olea europaea* cv. Leccino fruitlet *Olea europaea* cDNA	–8.67	0	Unknown
FL684062_1	FL684062 A_G16_D08_0414F_p2 *Olea europaea* cv. Leccino fruitlet *Olea europaea* cDNA	−8.28	0	Unknown
GO244680_1	GO244680 OEAA-070810_Plate5e16.b1 cDNA library from Olive leaves and fruits *Olea europaea* cDNA	8.12	0.00	Unknown
GO243351_1	GO243351 OEAA-070810_Plate1l16.b1 cDNA library from Olive leaves and fruits *Olea europaea* cDNA	8.17	0	Defense response to bacterium, photosynthetic electron transport in photosystem I, photosystem II assembly, regulation of proton transport, response to red light, response to blue light, response to far red light, response to high light intensity, response to karrikin, response to sucrose stimulus
GO246036_1	GO246036 OEAA-070810_Plate8o21.b1 cDNA library from Olive leaves and fruits *Olea europaea* cDNA	8.19	0	Salicylic acid metabolic process
GO246141_1	GO246141 OEAA-070810_Plate9d07.b1 cDNA library from Olive leaves and fruits *Olea europaea* cDNA	8.21	0	Metabolic process
FL684196_1	FL684196 A_D23_B12_0414F_p3 *Olea europaea* cv. Leccino fruitlet *Olea europaea* cDNA	8.32	0	Proteolysis
GO244977_1	GO244977 OEAA-070810_Plate6b15.b1 cDNA library from Olive leaves and fruits *Olea europaea* cDNA	8.63	0	Alcohol metabolic process, ovule development, oxidation-reduction process, petal development, stamen development
GO244976_1	GO244976 OEAA-070810_Plate6b14.b1 cDNA library from Olive leaves and fruits *Olea europaea* cDNA	8.88	0	Alcohol metabolic process, ovule development, oxidation-reduction process, petal development, stamen development

(−) indicates down-regulation.

### Transcriptome Analyses of Mature versus Juvenile Leaves

A total of 449 genes were found to be differentially expressed within “on-year” leaves at the mature (ON-M) and juvenile (ON-J) stages (two-fold change, *P*<0.05), the majority of them being up-regulated in the juvenile leaves (87.5%). The majority of the differentially expressed genes were related to cell organization and biogenesis, ion transport and homeostasis, lipid metabolic process, photosynthesis, oxidation-reduction, and responses to biotic and abiotic stresses (Table S2).

On the other hand, there were 217 differentially expressed genes between the juvenile (OFF-J) and the mature (OFF-M) “off-year” leaves. They were mainly annotated within the carbohydrate metabolic pathways, response to stresses, cell wall organization, and developmental processes. The regulation of most of the developmental processes and carbohydrate metabolism genes were increased in the mature leaves in relation to the immature ones (Table S2).

The analyses revealed that 36 transcripts were differentially expressed between juvenile and mature leaves, irrespective of the bearing of the plants. Among those, 32 transcripts showed developmental stage-specific expression patterns in leaves (Table S3). For example, the probe ID GO245535_1 was more expressed in mature leaves than in juvenile ones of both “on” and “off” years. A similar pattern was observed for the expression of FL683438_1, being down-regulated with leaf maturation, irrespective of the timing. The GO analyses showed that those 36 genes were mainly involved in the cell wall biogenesis, transcription regulation, growth, proteolysis, and biotic/abiotic stress responses.

### Transcriptome Analyses of “on” versus “off” Year Leaves

Comparing the mature leaves of both years (ON-M and OFF-M), 230 genes were found to be differentially expressed (Table S4), which were mainly involved in ion transport and homeostasis, hormone response, developmental processes, response to stress, lipid metabolism, oxidation-reduction, growth and development, transport, response to chemical stimuli, cell organization and biogenesis, and other cellular and metabolic processes. On the other hand, only 16 transcripts were identified to be differentially expressed between juvenile leaves of “on” (ON-J) and “off” (OFF-J) years. Of those, the majority had unknown biological roles (Table S4). Taking these two comparisons into account, only one transcript was found to be common. However, the probe FL684126_1, playing a role in the flavonoid biosynthetic processes, was more expressed (2.13-fold) in the “on-year” juvenile leaves (ON-J) than in the “off-year” juvenile ones (OFF-J), while its expression was 4.76-fold down-regulated in the “on-year” mature (ON-M) relative to the “off-year” mature (OFF-M) leaves.

The comparison between the “on” and “off” year transcripts showed that a wider set of transcripts were differentially regulated in the mature leaves than in the juvenile ones. In addition to that, the gene ontology analyses revealed that the majority of the transcripts related to oxidation-reduction, carbohydrate metabolism, ion transport and homeostasis were up-regulated in the “off” year of the mature leaves, while the juvenile leaves did not reveal a similar pattern (Table 3).

**Table 3:** List of differentially expressed transcripts associated to oxidation-reduction metabolism, carbohydrate metabolism, ion transport and homeostasis between “on” and “off” years.

Seq_ID	Description	ON_M/OFF_M_fold change	*P* value	GO biological process	KEGG pathway
GO244165_1	GO244165 OEAA-070810_Plate3o13.b1 cDNA library from Olive leaves and fruits *Olea europaea* cDNA	–3.43	0.00	Transmembrane transport, water transport, response to abscisic acid stimulus	Unknown
GO243991_1	GO243991 OEAA-070810_Plate3h07.b1 cDNA library from Olive leaves and fruits *Olea europaea* cDNA	–3.35	0.00	Oxidation-reduction process, fatty acid metabolic process	Fatty acid metabolism, retinol metabolism, steroid hormone biosynthesis, linoleic acid metabolism, drug metabolism - cytochrome P450, drug metabolism - other enzymes, metabolism of xenobiotics by cytochrome P450, caffeine metabolism, aminobenzoate degradation, tryptophan metabolism
GO243114_1	GO243114 OEAA-070810_Plate1b09.b1 cDNA library from Olive leaves and fruits *Olea europaea* cDNA	–3.31	0.00	Flavonol biosynthetic process, oxidation-reduction process, response to karrikin, response to light stimulus	Flavonoid biosynthesis
GO244094_1	GO244094 OEAA-070810_Plate3l14.b1 cDNA library from Olive leaves and fruits *Olea europaea* cDNA	–3.24	0.00	Fatty acid metabolic process, oxidation-reduction process	Fatty acid metabolism, retinol metabolism, steroid hormone biosynthesis, linoleic acid metabolism, drug metabolism - cytochrome P450, drug metabolism - other enzymes, metabolism of xenobiotics by cytochrome P450, caffeine metabolism, aminobenzoate degradation, tryptophan metabolism
GO243842_1	GO243842 OEAA-070810_Plate3b01.b1 cDNA library from Olive leaves and fruits *Olea europaea* cDNA	–2.97	0.00	Oxidation-reduction process, amine metabolic process	Isoquinoline alkaloid biosynthesis, phenylalanine metabolism, glycine, serine and threonine metabolism, tyrosine metabolism, beta-Alanine metabolism, Tropane, piperidine and pyridine alkaloid biosynthesis
GO243358_1	GO243358 OEAA-070810_Plate1l24.b1 cDNA library from Olive leaves and fruits *Olea europaea* cDNA	–2.96	0.00	Oxidation-reduction process, amine metabolic process, amine metabolic process	Isoquinoline alkaloid biosynthesis, phenylalanine metabolism, glycine, serine and threonine metabolism, tyrosine metabolism, beta-Alanine metabolism, Tropane, piperidine and pyridine alkaloid biosynthesis
GO243043_1	GO243043 OEAA-070810_Plate10o07.b1 cDNA library from Olive leaves and fruits *Olea europaea* cDNA	–2.95	0.00	Transmembrane transport, response to abiotic stimulus, monovalent inorganic cation transport, response to chemical stimulus, calcium ion transport, cellular divalent inorganic cation homeostasis, response to stress	Unknown
GO243363_1	GO243363 OEAA-070810_Plate1m05.b1 cDNA library from Olive leaves and fruits *Olea europaea* cDNA	–2.93	0.00	Oxidation-reduction process, fatty acid metabolic process	Fatty acid metabolism, retinol metabolism, steroid hormone biosynthesis, linoleic acid metabolism, drug metabolism - cytochrome P450, drug metabolism - other enzymes, metabolism of xenobiotics by cytochrome P450, caffeine metabolism, aminobenzoate degradation, tryptophan metabolism
GO245033_1	GO245033 OEAA-070810_Plate6d24.b1 cDNA library from Olive leaves and fruits *Olea europaea* cDNA	–2.82	0.01	Fatty acid metabolic process, oxidation-reduction process	Fatty acid metabolism, retinol metabolism, steroid hormone biosynthesis, linoleic acid metabolism, drug metabolism - cytochrome P450, drug metabolism - other enzymes, metabolism of xenobiotics by cytochrome P450, caffeine metabolism, aminobenzoate degradation, tryptophan metabolism
GO245373_1	GO245373 OEAA-070810_Plate7c14.b1 cDNA library from Olive leaves and fruits *Olea europaea* cDNA	–2.80	0.00	Cellular divalent inorganic cation homeostasis, monovalent inorganic cation transport, response to abiotic stimulus, response to chemical stimulus, response to stress, transmembrane transport, calcium ion transport	Unknown
GO242798_1	GO242798 OEAA-070810_Plate10d24.b1 cDNA library from Olive leaves and fruits *Olea europaea* cDNA	–2.55	0.00	Carbohydrate metabolic process	Other glycan degradation, cyanoamino acid metabolism
GO243051_1	GO243051 OEAA-070810_Plate10o15.b1 cDNA library from Olive leaves and fruits *Olea europaea* cDNA	–2.53	0.00	Monovalent inorganic cation transport, response to abiotic stimulus, response to chemical stimulus, response to stress, transmembrane transport, calcium ion transport, cellular divalent inorganic cation homeostasis	Unknown
GO243038_1	GO243038 OEAA-070810_Plate10o02.b1 cDNA library from Olive leaves and fruits *Olea europaea* cDNA	–2.49	0.00	Monovalent inorganic cation transport, response to abiotic stimulus, response to chemical stimulus, response to stress, transmembrane transport, calcium ion transport, cellular divalent inorganic cation homeostasis	Unknown
GO243685_1	GO243685 OEAA-070810_Plate2j24.b1 cDNA library from Olive leaves and fruits *Olea europaea* cDNA	–2.42	0.01	Brassinosteroid biosynthetic process, response to oxidative stress, trichoblast differentiation, oxidation-reduction process, nitrate transport, response to desiccation, response to cold, hyperosmotic salinity response, cellular response to iron ion starvation, iron ion transportresponse to nitrate	Phenylalanine metabolism, phenylpropanoid biosynthesis, methane metabolism
GO244702_1	GO244702 OEAA-070810_Plate5f15.b1 cDNA library from Olive leaves and fruits *Olea europaea* cDNA	–2.42	0.00	Calcium ion transport, cellular cation homeostasis, water transport, methylammonium transmembrane transport, urea transmembrane transport, response to salt stress	Unknown
GO246108_1	GO246108 OEAA-070810_Plate9b22.b1 cDNA library from Olive leaves and fruits *Olea europaea* cDNA	–2.39	0.00	Monovalent inorganic cation transport, response to abiotic stimulus, response to chemical stimulus, response to stress, transmembrane transport, calcium ion transport, cellular divalent inorganic cation homeostasis	Unknown
GO243337_1	GO243337 OEAA-070810_Plate1l02.b1 cDNA library from Olive leaves and fruits *Olea europaea* cDNA	–2.38	0.02	Oxidation-reduction process, lipid metabolic process	GO243337_1
FL683558_1	FL683558 A_M17_G09_0414F_p1 *Olea europaea* cv. Leccino fruitlet *Olea europaea* cDNA	–2.32	0.00	Glycolysis, golgi organization, hyperosmotic response, regulation of protein localization, response to cadmium ion, response to salt stress, response to temperature stimulus, response to water deprivation, transmembrane transport, water transport, carbon dioxide transport	Unknown
GO242745_1	GO242745 OEAA-070810_Plate10b19.b1 cDNA library from Olive leaves and fruits *Olea europaea* cDNA	–2.32	0.00	Monovalent inorganic cation transport, response to abiotic stimulus, response to chemical stimulus, response to stress, transmembrane transport, calcium ion transport, cellular divalent inorganic cation homeostasis	Unknown
GO246184_1	GO246184 OEAA-070810_Plate9f02.b1 cDNA library from Olive leaves and fruits *Olea europaea* cDNA	–2.24	0.01	Monovalent inorganic cation transport, response to abiotic stimulus, response to chemical stimulus, response to stress, transmembrane transport, calcium ion transport, cellular divalent inorganic cation homeostasis	Unknown
eugene3.00040001	SKS4 (SKU5 Similar 4)	–2.22	0.00	Oxidation-reduction process	Unknown
GO245788_1	GO245788 OEAA-070810_Plate8e07.b1 cDNA library from Olive leaves and fruits *Olea europaea* cDNA	–2.22	0.00	Oxidation-reduction process	Amino sugar and nucleotide sugar metabolism, isoquinoline alkaloid biosynthesis, peptidoglycan biosynthesis
FN998444_1	FN998444 FN998444 *Olea europaea* flower *Olea europaea* cDNA clone c2-2-F11	–2.21	0.04	Carbohydrate metabolic process	Unknown
GO242755_1	GO242755 OEAA-070810_Plate10c05.b1 cDNA library from Olive leaves and fruits *Olea europaea* cDNA	–2.21	0.02	Transport	Transport
GO246009_1	GO246009 OEAA-070810_Plate8n16.b1 cDNA library from Olive leaves and fruits *Olea europaea* cDNA	–2.19	0.01	Monovalent inorganic cation transport, response to abiotic stimulus, response to chemical stimulus, response to stress, transmembrane transport, calcium ion transport, cellular divalent inorganic cation homeostasis	Unknown
GO246175_1	GO246175 OEAA-070810_Plate9e17.b1 cDNA library from Olive leaves and fruits *Olea europaea* cDNA	–2.18	0.01	Monovalent inorganic cation transport, response to abiotic stimulus, response to chemical stimulus, response to stress, transmembrane transport, calcium ion transport, cellular divalent inorganic cation homeostasis	Unknown
GO245712_1	GO245712 OEAA-070810_Plate8b01.b1 cDNA library from Olive leaves and fruits *Olea europaea* cDNA	–2.17	0.00	Monovalent inorganic cation transport, response to abiotic stimulus, calcium ion transport, cellular divalent inorganic cation homeostasis, response to chemical stimulus, response to stres, transmembrane transport	Unknown
GO244028_1	GO244028 OEAA-070810_Plate3i20.b1 cDNA library from Olive leaves and fruits *Olea europaea* cDNA	–2.15	0.00	Oxidation-reduction process, fatty acid biosynthetic process	Glycolysis/Gluconeogenesis, glycine, serine and threonine metabolism, fatty acid metabolism, tyrosine metabolism, retinol metabolism, biosynthesis of unsaturated fatty acids, drug metabolism - cytochrome P450, metabolism of xenobiotics by cytochrome P450, naphthalene degradation, chloroalkane and chloroalkene degradation,
GO242772_1	GO242772 OEAA-070810_Plate10c22.b1 cDNA library from Olive leaves and fruits *Olea europaea* cDNA	–2.14	0.00	Monovalent inorganic cation transport, response to abiotic stimulus, response to chemical stimulus, response to stress, calcium ion transport, cellular divalent inorganic cation homeostasis, transmembrane transport	Unknown
GO242993_1	GO242993 OEAA-070810_Plate10m05.b1 cDNA library from Olive leaves and fruits *Olea europaea* cDNA	–2.11	0.01	Monovalent inorganic cation transport, response to abiotic stimulus, response to chemical stimulus, response to stress, transmembrane transport, calcium ion transport, cellular divalent inorganic cation homeostasis,	Unknown
GO244140_1	GO244140 OEAA-070810_Plate3n12.b1 cDNA library from Olive leaves and fruits *Olea europaea* cDNA	–2.11	0.01	Oxidation-reduction process, fatty acid metabolic process	Fatty acid metabolism, retinol metabolism, steroid hormone biosynthesis, linoleic acid metabolism, drug metabolism - cytochrome P450, drug metabolism - other enzymes, metabolism of xenobiotics by cytochrome P450, caffeine metabolism, aminobenzoate degradation, tryptophan metabolism
GO246093_1	GO246093 OEAA-070810_Plate9b07.b1 cDNA library from Olive leaves and fruits *Olea europaea* cDNA	–2.11	0.00	Monovalent inorganic cation transport, response to abiotic stimulus, response to chemical stimulus, response to stress, transmembrane transport, calcium ion transport, cellular divalent inorganic cation homeostasis	Unknown
FL684145_1	FL684145 C_I19_E10_0414F_p9 *Olea europaea* cv. Leccino fruitlet *Olea europaea* cDNA	–2.10	0.01	Glycolysis, lipid metabolic process, response to cadmium ion, phosphorylation	Glycolysis/Glucongeogenesis, carbon fixation in photosynthetic organisms, purine metabolism, pyruvate metabolism,
FN998690_1	FN998690 FN998690 *Olea europaea* flower *Olea europaea* cDNA clone c2-6-D1	–2.10	0.01	Oxidation-reduction process, proteolysis, response to ethylene stimulus, aging, defense response to fungus, incompatible interaction	Unknown
GO246394_1	GO246394 OEAA-070810_Plate9o05.b1 cDNA library from Olive leaves and fruits *Olea europaea* cDNA	–2.10	0.01	Monovalent inorganic cation transport, response to abiotic stimulus, response to chemical stimulus, response to stres, transmembrane transport, calcium ion transport, cellular divalent inorganic cation homeostasis	Unknown
GO242964_1	GO242964 OEAA-070810_Plate10k23.b1 cDNA library from Olive leaves and fruits *Olea europaea* cDNA	–2.09	0.05	Monovalent inorganic cation transport, response to abiotic stimulus, response to chemical stimulus, response to stress, transmembrane transport, calcium ion transport, cellular divalent inorganic cation homeostasis	Unknown
GO245065_1	GO245065 OEAA-070810_Plate6f08.b1 cDNA library from Olive leaves and fruits *Olea europaea* cDNA	–2.08	0.00	Oxidation-reduction process	Unknown
GO245725_1	GO245725 OEAA-070810_Plate8b16.b1 cDNA library from Olive leaves and fruits *Olea europaea* cDNA	–2.08	0.00	Monovalent inorganic cation transport, transmembrane transport, cellular divalent inorganic cation homeostasis, calcium ion transport, response to abiotic stimulus, response to stress, response to chemical stimulus	Unknown
GO245986_1	GO245986 OEAA-070810_Plate8m17.b1 cDNA library from Olive leaves and fruits *Olea europaea* cDNA	_2.07	0.01	Monovalent inorganic cation transport, transmembrane transport, cellular divalent inorganic cation homeostasis, calcium ion transport, response to abiotic stimulus, response to stress, response to chemical stimulus	Unknown
GO242886_1	GO242886 OEAA-070810_Plate10h17.b1 cDNA library from Olive leaves and fruits *Olea europaea* cDNA	_2.05	0.00	Transmembrane transport, response to abiotic stimulus, monovalent inorganic cation transport, response to chemical stimulus, calcium ion transport, cellular divalent inorganic cation homeostasis, response to stress	Unknown
GO243107_1	GO243107 OEAA-070810_Plate1b01.b1 cDNA library from Olive leaves and fruits *Olea europaea* cDNA	_2.04	0.00	Oxidation-reduction process, abscisic acid mediated signalling pathway, cellular response to water deprivation, response to hydrogen peroxide, toxin catabolic process	Arachidonic acid metabolism, glutathione metabolism
GO245678_1	GO245678 OEAA-070810_Plate7p14.b1 cDNA library from Olive leaves and fruits *Olea europaea* cDNA	_2.04	0.00	Oxidation-reduction process, response to oxidative stress, response to salt stres, trichoblast differentiation,	Phenylalanine metabolism, phenylpropanoid biosynthesis, methane metabolism
GO244126_1	GO244126 OEAA-070810_Plate3m22.b1 cDNA library from Olive leaves and fruits *Olea europaea* cDNA	_2.03	0.00	Photosynthetic electron transport chain, response to karrikin, response to light stimulus, ferredoxin metabolic process	Unknown
GO246079_1	GO246079 OEAA-070810_Plate9a17.b1 cDNA library from Olive leaves and fruits *Olea europaea* cDNA	_2.01	0.00	Monovalent inorganic cation transport, transmembrane transport, cellular divalent inorganic cation homeostasis, calcium ion transport, response to abiotic stimulus, response to stress, response to chemical stimulus	Unknown

Fold changes were given in log2-based numbers. (-) indicates down-regulation.

### Validation of Microarray Data by Quantitative Reverse-transcription PCR

**Figure 5:**
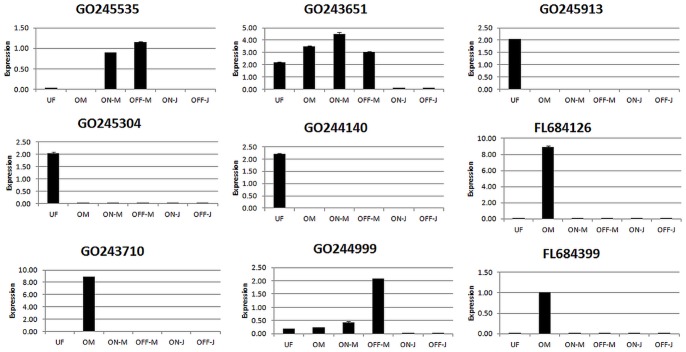
Comparison of expression levels of selected genes calculated by qRT-PCR. Gene expressions represented are normalized to the 18S rRNA.

The microarray gene expression data were validated by qRT-PCR (Table S5). The differentially expressed genes were randomly selected representing up-regulated and down-regulated genes in the microarray analyses. The qRT-PCR gene expression measurements consistent with microarray data are shown in Figure 5. The data shows a high compatibility between the two analyses. The expression pattern of GO245535 in microarray analysis was almost identical to that of qRT-PCR result. Similarly, both analyses revealed comparable expression patterns for GO243651, GO245913, GO244140, FL68499 probes. The other tested probes, although the gene expression profile was generally consistent, there were some minor differences between the analyses. For example, GO244999 was up-regulated in ON-J relative to OFF-J in microarray analysis, whereas it was found to be the opposite in qRT-PCR. However, the majority of the data obtained by two methods was compatible with each other. Thus, these data confirms the accuracy of the oligo microarray method to analyze the expression of the olive tree transcripts.

## Discussion

The miRNA associated to the developmental phase transition in the olive tree have been reported [16]. On the other hand, we have previously carried out a deep sequencing of olive tree samples at different developmental phases [30]. The tendency of the olive trees to alternate bearing is a well-known phenomenon [5]. In this study, the gene expression profiles associated with different tissues and developmental stages were examined, comparing the gene expression of six olive tree samples. To our knowledge, this is the first report on microarray gene expression profiling associated to alternate bearing in the olive tree.

Although cross-species array hybridization has certain potential risks, in literature, several studies showed that heterologous microarrays provide confident results [31], [32], [33], [34]. Sequencing of the whole genome of olive has been initiated by the International Olive (*Olea europea*) Genome Consortium (IOGC) has been started (http://olivegenome.karatekin.edu.tr/), however a scarce sequence information of olive is available at the moment. Therefore,in our analysis, the probes derived from poplar EST sequences, together with the data for all the publicly available olive ESTs were used. The analysis showed that poplar probes could be applied reliably for olive microarray analysis.

The plant species exhibit certain specific processes, with the flowering-site limitations, endogenous hormonal controls, and nutritional controls having major impacts on the physiological processes of periodicity [5]. Our analyses show that some other factors play important roles on this phenomenon. Additionally, some olive tree transcripts could not be associated with a specific function. As previously reported, the poor annotation of the olive tree genes can be due to quite divergent gene functions in such species in relation to other plants [35].

Additionally, for qRT-PCR measurement to validate the microarray data, expression level of 9 randomly selected transcripts shown in Figure 5 were confirmed. However, there were some differences between microarray and qRT-PCR results. Possibly, the interspecies cross hybridization of populus ESTs with olive cDNA/mRNA transcripts led this minor discrepancies. Although some of the reference gene candidates such as *18s rRNA*, *GAPDH,* and *beta-tubulin* were measured for normalizations, the *18s rRNA* gene was found to be the useful alternative as the housekeeping gene for olive qRT-PCR normalization.

### Nutritional Status

The relationships between the alternate bearing and the carbohydrate storage have been indicated by a number of studies. Thus, Rosecrance et al. [6] showed that the pistachio trees entering the “off” year required low amounts of carbohydrates, due to the low fruiting at the time, and thus accumulated some starch. Additionally, the citrus trees also showed similar carbohydrate accumulation patterns [36]. Similarly, Spann et al. [37] found that the bearing and non-bearing pistachio trees differed in their carbohydrate storage and mobilization patterns, and suggested that the in-season carbon mobilization might have an effect on the alternate bearing. In conclusion, the studies carried out so far indicate that the nutrients are stored during the “off” year, and that they are used for reproductive growth the following year in pistachio trees. On the other hand, conflicting results for the relevance of the carbohydrate storage on reproductive development in olive trees have been reported. Thus, Ulger et al. [7] indicated that the carbohydrates and mineral nutrients had trivial influence on the flower bud formation in the olive trees. Also, Bustan et al. [38] suggested that the status of the carbohydrate reserves was not the main determinant for the alternate bearing, being rather involved in the survival of the olive trees. Additionally, comparable amounts of carbohydrates were found in the leaves of “on” and “off” year olive trees [39]. On the contrary, the starch content was found to be increased in winter, and decreased during bud development in the olive tree [40]. In addition to the carbohydrates, the mineral contents of the olive tree leaves was investigated, and the results showed that the leaf-nutrient content fluctuated between “on” and “off” years [41], [42], [43].

In the present study, the microarray analyses of six olive tree samples indicated that the expression of the genes involved in the carbohydrate processes varied between samples. In comparison to bearing year leaves, the non-bearing year leaves had elevated expressions of genes responsible for the carbohydrate metabolism (Table S3). In addition, the genes for the mineral transport were also up-regulated in the “off” year leaves. Supporting the present study, we have found a lower expression of the miRNA targeting minerals and carbohydrate biosynthesis and transport genes in bearing year leaves, in a high-throughput sequencing study of miRNA in olive tree leaves [30]. In the view of the present study, we conclude that, as found in pistachios, the olive trees also use more nutrients in the “off” year. Hence, the regulation of the nutrients plays a major role in the alternate bearing in this species.

### Developmental Stages and Organ-specific Gene Expressions

Different transcripts with the same predicted annotation revealed sometimes opposite expression patterns between the developing stages, which can be explained due to the adjustment of the metabolic pathways and the presence of alternative metabolites in the olive tree [35]. However, the data suggested that the regulations of certain processes were correlated with the developmental stage and bearing of the olive tree.

The analyses indicated that there was a striking difference between the two studied organs. Thus, the transcriptome profiles of the fruits were clearly different than those of the leaves. Moreover, the profiles of the ripe fruits showed obvious differences in relation to that of unripe fruits, with abundant differentially expressed genes. Similarly, the blueberry fruits revealed different transcriptome profiles at different developmental stages [44]. The photosynthesis is an essential process to promote the fruit growth. As stated by Alagna et al [35], during the fruit growth the main energy source is the photosynthesis, while at the end of the ripening it is the mitochondrial respiration of photoassimilates. Consequently, decreased expression of transcripts related to photosynthesis in the ripe fruits in relation to the unripe fruits. Consistent with that, the transcripts related to photosynthesis were mainly up-regulated in unripe fruits in relation to the ripe fruits. Supportingly, the KEGG analyses also revealed that the transcripts involved in the photosynthesis pathways were increased in the unripe fruits. Supportingly, the transcripts associated with the carbohydrate metabolism were more expressed in the mature leaves, as compared to the juvenile ones, while the opposite expression patterns were observed for the photosynthesis-related transcripts.

A higher proportion of transcripts showed altered expressions between the samples at different developmental stages (mature and juvenile) than between the samples harvested at different timings (“on and off” years). Thus, a total of 32 differentially expressed genes between mature and juvenile leaves showed comparable expression patterns for both “on” and “off” years (Table S3), suggesting a common response to environmental factors such as temperature rather than fruit bearing status of the trees.

The amount of total proteins in the leaves and young shoots of the olive tree was found to be opposite in “on” and “off” years, which indicates that different proteins were induced during the “on” and “off” years in the olive trees [2]. In congruence with that, our analyses showed that the “on” and “off” year leaves shared just one common differentially expressed gene, (probe FL684126) indicating a striking effect of tree load on the EST profiles of the olive tree samples.

According to the comparisons between the juvenile and the mature leaves, a large set of differentially expressed transcripts was related to the cell organization and biogenesis. The higher gene expression levels of those in the juvenile leaves in relation to the mature ones may be explanined by the active status of most biological processes including photosynthesis, growth, fruit nourishment, transport from source to sink tissues and cell division comparing to a relatively less number of metabolic and cellular acitivies in a phase close to winter. Another difference between the two olive tree samples was observed for the transcripts associated with the lipid metabolism. Being congruent with the deep sequencing data [31], the higher expression of transcripts related to the fatty-acid biosynthesis in mature leaves clearly depicted the fatty-acid accumulation in the mature olive fruits.

The redox state regulates a wide variety of biological processes. Indeed, the plant growth and development are driven by oxidation-reduction reactions. The plant growth is adjusted by the redox state of the apoplast in tobacco [45]. The Pyridine Nucleotides (PN) are key components of the redox reactions, being therefore developmental cues for the transitioning from the vegetative to the reproductive states in the spinach leaves [46]. Additionally, the environmental stimuli alter the redox state and trigger the plant defense. The Reactive Oxygen Species (ROS), produced as a consequence of the electron transport processes in photosynthesis and respiration, induce alterations in the cellular redox state and have a positive influence on the plant growth [47]. Indeed, the olive tree microarray data showed that the redox-associated transcripts were higher in the juvenile leaves than in the mature ones of the bearing year. Since the induction in the olive tree starts in July, an elevated expression of those transcripts means a continuation of the development in the juvenile leaves.

Additionally, the comparison between the mature bearing and the non-bearing leaves showed that almost all of the transcripts related to the oxidation-reduction cascade were significantly up-regulated in the non-bearing leaves. In connection with that, owing to the occurrence of the high redox activity triggering the stress-signals, several stress-related transcripts were up-regulated in the non-bearing leaves in relation to the bearing ones. As an example, the photosynthesis involving the reduction of the carbon dioxide into sugars is a well-known redox process. Thus, elevated expressions of the transcripts belonging to the photosynthesis in “off” year in relation to the “on” year were observed for citrus [48]. Besides, the photosynthesis was inhibited by the bud morphology in the “on” year, whereas “off” year leaves were filled with photoassimilates. In fact, it has been proposed that its induction in “off” citrus buds provides a leaf signal indicating the available nutrition richness. Similarly, the pistachio trees accumulated more carbohydrate during “off” years in relation to the “on” ones [6]. Thus, Goldschmidt [49] supported the regulatory role of the photoassimilate availability for the flowering induction. The comparison between the mature bearing and non-bearing leaves also showed that the ion transport- and homeostasis-related transcripts were more expressed in the non-bearing leaves than in the bearing ones. The KEGG analyses of those up-regulated transcript in the non-bearing leaves indicated that they were mainly involved in the lipid and amino acid metabolism, xenobiotic biodegradation and metabolism, biosynthesis of secondary metabolites, and carbohydrate and energy metabolism. Interestingly, the xenobiotic biodegradation and metabolism participates in the defense mechanisms. Indeed, a relationship between the carbohydrate nutritional status and the responses to the xenobiotics has been found in *Arabidopsis thaliana*, showing that the presence of sugars triggered the defense mechanisms [50].

On the other hand, the flavonoids controlling the aroma and flavor are secondary metabolites, being synthesized in response to an excess of photoassimilation [51]. The expression level of the transcripts related with flavonoid biosynthesis were increased in the “off” year leaves in relation to the “on” year ones in the olive tree. A similar gene expression pattern was observed with citrus buds, being based on flavonoids acting as a reservoir for the photoassimilation surplus [48]. Taking account the higher expression of transcripts related to the oxidation-reduction, carbohydrate metabolism and mineral transport, together with flavonoid biosynthesis in “off” year leaves, we conclude that the nutritional status may be the principal key controlling the alternate bearing in the olive tree. Supporting the present work, a wide range of the genes targeted by the olive tree miRNA were found to be mainly involved in the carbohydrate metabolic pathways [30].

### Flowering

Although an olive tree typically produces a large amount of flowers when it blossoms, only a small percentage of them become fruits in the “on” year. In fact, it has been found that when an olive tree enters an ‘on’ year, the flower and inflorescence density is not correlated to the fruit density [52].

Interestingly, the fructification process alters both the floral bud differentiation and the flowering induction, being recognized as an inhibitor of the flowering in the fruit trees as previously indicated. Thus, the “off” year leaves showed significantly increased expression levels of the genes *cift* (citrus flowering locus T) and *soc1* (suppressor of overexpression of constants 1), which are responsible for flowering, as compared with those from the “on” year leaves in mandarin [53]. However, the microarray data of the present work indicates that the expression level of the only one transcript related to flower development (GO243632_1) was altered among the “on” and “off” year leaves. The expression of GO243632_1 was two-fold higher in the “off” year leaves as compared to the “on” year ones in the olive tree. Nevertheless, the microarray data suggests that the flowering is not the main factor in the alternate bearing in the olive tree.

### Endogenous Plant Hormones

Previous studies have indicated that the endogenous plant-growth hormones influence the alternate bearing. In fact, significant differences were revealed in the presence of some endogenous plant growth hormones including the abscisic acid (ABA), gibberellins like the gibberellic acids (GA_3_ and GA_4_) and auxins like the indole-3-acetic acid (IAA), between the “on” and “off” years for the olive tree samples [7], [54]. Thus, the floral formation was inhibited in the presence of high GA_3_ levels, whereas the application of high concentrations of GA_4_, ABA and cytokinins resulted in elevated levels of flower formation in the olive tree [7]. It has been stated that high levels of GA_3_ caused vegetative growth, negatively affecting the generative bud development in the following year. The “on” and “off” year buds had equal amounts of ABA in orange, being suggested that the ABA was not related to the alternate bearing. On the other hand, the 2-trans-abscisic acid (*t-*ABA) was almost twice the concentration of the ABA in the “on” year buds, with the difference decreasing at later sampling dates. Consequently, the impact of the *t*-ABA on the bud dormancy was proposed in orange [55]. On the contrary, it has been found that the “off” year olive trees produced more ABA than the “on” year ones [56].

The olive tree microarray analyses of this work showed that among 246 differentially expressed genes between the “on” and “off” year leaves, irrespective to their developmental stage, only 14 genes were found to be associated to hormone regulation (Table S4). Of these, five genes (probes fgenesh4_pg.C_scaffold_19987000001, eugene3.00160596, eugene3.00020895, eugene3.00170500 and FL683585_1) showed significantly elevated expressions in the “on” year leaves, as compared to the “off” year ones. The other nine transcripts (probes grail3.0111003801, GO245518_1, GO245517_1, GO243107_1, GO245994_1, FN998690_1, GO244677_1, GO243685_1 and gw1.VII.2355.1) revealed opposite expression patterns. Lavee [57] reported that the phytohormones were present at lower levels in the olive tree than in other fruit trees. Consequently, our results indicate that although the endogenous hormones had an influence on the alternate bearing at a certain level, they were not the key determinants of this phenomenon in the olive tree.

In summary, a total of 136,628 oligonucleotide probe sets were arrayed in this first microarray gene expression profiling of six *O. europaea* samples from fruits and leaves. The gene expression profiles with regard to the different tissues and developmental stages were examined. The expression of the transcripts greatly varied among the six studied libraries, indicating the involvement of diverse processes in response to bearing. The expressions of the transcripts for different organs under different developmental phases indicated that the nutrition metabolism had a remarkable impact on the olive tree alternate bearing. Additionally, the hormonal control also played relevant roles in this complex phenomenon.

## Supporting Information

Table S1 **Normalized signal intensities of the 54,515 probes.**
(TXT)

Table S2 **Differentially expressed transcripts between juvenile and mature leaves.** Fold changes were given in log2-based numbers. (−) indicates down-regulation.
(DOCX)

Table S3 **Differentially expressed transcripts between mature and juvenile leaves, irrespective to the bearing year.** Fold changes were given in log2-based numbers. (−) indicates down-regulation.
(DOCX)

Table S4 **Differentially expressed transcripts between the “on” and “off” years.** Fold changes were given in log2-based numbers. (−) indicates down-regulation.
(DOCX)

Table S5 **The raw qRT-PCR data.**
(DOCX)

## References

[1] Besnard G, Henry P, Wille L, Cooke D, Chapuis D (2007) On the origin of the invasive olives (*Olea europaea* L., Oleaceae). Heredity 99: 608–619. 17687251 10.1038/sj.hdy.6801037

[2] Lavee S (2007) Biennial bearing in olive (*Olea europaea*). Annales Ser His Nat 17: 101–112.

[3] Monselise SP, Goldschmidt EE (1982) Alternate bearing in fruit trees. Hortic Reviews 4: 128–173.

[4] Fernández-Ocaña A, García-López MC, Jiménez-Ruiz J, Saniger L, Macías D, et al (2010) Identification of a gene involved in the juvenile-to-adult transition (JAT) in cultivated olive trees. Tree Genet Genomes 6: 891–903.

[5] Goldschmidt EE (2005) Regulatory aspects of alternate-bearing in fruit trees. Italus Hortus 12: 11–17.

[6] Rosecrance RC, Weinbaum SA, Brown PH (1998) Alternate bearing affects nitrogen, phosphorus, potassium and starch storage pools in mature pistachio trees. Ann Bot - London 82: 463–470.

[7] Baktir I, Ulger S, Kaynak L, Himelrick DG (2004) Relationship of seasonal changes in endogenous plant hormones and alternate bearing of olive trees. Hort Science 39(5): 987–990.

[8] Barranco D, Fernández-Escobar R, Rallo L (Eds): “Olive Growing”. 1^st^ English Edition of the 5^th^ revised and enlarged edition of “El Cultivo del Olivo”, Mundi-Prensa - Junta de Andalucía - Australian Olive Association (Rural Industries Research and Development Corporation; RIRDC). The 6^th^ edition (2008) is available in Spanish, Mundi-Prensa - Junta de Andalucía (Madrid, Spain) 2010.

[9] Ulger S, Sonmez S, Karkacier M, Ertoy N, Akdesir O, et al (2004) Determination of endogenous hormones, sugars and mineral nutrition levels during the induction, initiation and differentiation stage and their effects on flower formation in olive. Plant Growth Regul 42: 89–95.

[10] Ben-Gal A, Yermiyahu U, Zipori I, Presnov E, Hanoch E, et al (2011) The influence of bearing cycles on olive oil production response to irrigation. Irrig Sci 29: 253–263. 10.1021/jf202324x 21950468

[11] Naor A, Schneider D, Ben-Gal A, Zipori I, Dag A et al.. (2012) The effects of crop load and irrigation rate in the oil accumulation stage on oil yield and water relations of ‘Koroneiki’ olives. Irrig Sci doi: 10.1007/s00271-012-0363-z.

[12] Martín-Vertedor AI, Rodríguez JMP, Losada HP, Castiel EF (2011) Interactive responses to water deficits and crop load in olive (olea europaea L., cv. Morisca) I. – Growth and water relations. Agr Water Manage 98: 941–949.

[13] Dag A, Bustan A, Avni A, Tzipori I, Lavee S, et al (2010) Timing of fruit removal affects concurrent vegetative growth and subsequent return bloom and yield in olive (*Olea europaea* L.). Sci Hortic-Ansterdam 123: 469–472.

[14] Huijser P, Schmid M (2011) The control of developmental phase transitions in plants. Development 138: 4117–4129. 21896627 10.1242/dev.063511

[15] Ozdemir-Ozgenturk N, Oruç F, Sezerman U, Kucukural A, Vural Korkut S et al.(2010) Generation and analysis of expressed sequence tags from *Olea europaea* L. Comp Funct Genom doi:10.1155/2010/757512. 10.1155/2010/757512 PMC3004401 21197085

[16] Donaire L, Pedrola L, de la Rosa R, Llave C (2011) High-throughput sequencing of RNA silencing-associated small RNAs in olive (*Olea europaea* L.). PLoS One 6(11): e27916 doi:10.1371/journal.pone.0027916 22140484 10.1371/journal.pone.0027916 PMC3225373

[17] Lai Z, Gross BL, Zou Y, Andrews J, Rieseberg LH (2006) Microarray analysis reveals differential gene expression in hybrid sunflower species. Mol Eco 15: 1213–1227. 10.1111/j.1365-294X.2006.02775.x PMC2536761 16626449

[18] Wellmer F, Alves-Ferreira M, Dubois A, Riechmann JL, Meyerowitz EM (2006) Genome-wide analysis of gene expression during early Arabidopsis flower development PLoS Genet. 2(7): 1012–1024. 10.1371/journal.pgen.0020117 PMC1523247 16789830

[19] Roach MJ, Dayholos MK (2007) Microarray analysis of flax (*Linum usitatissimum* L.) stems identifies transcripts enriched in fibre-bearing phloem tissues. Mol Genet Genomics 278 (2): 149–165. 10.1007/s00438-007-0241-1 17503083

[20] Fenart S, Assoumou Ndong YP, Duorte J, Rivière N, Wilmer J, et al (2010) Development and validation of a flax (*Linum usitatissimum* L.) gene expression oligo microarray. BMC Genomics 11: 592. 20964859 10.1186/1471-2164-11-592 PMC3091737

[21] Unver T, Bakar M, Shearman RC, Budak H (2010) Genome-wide profiling and analysis of *Festuca arundinacea* miRNAs and transcriptomes in response to foliar glyphosate application. Mol Genet Genomics 283: 397–413. 20213187 10.1007/s00438-010-0526-7

[22] Bonghi C, Trainotti L, Botton A, Tadiello A, Rasori A, et al (2011) A microarray approach to identify genes involved in seed-pericarp cross-talk and development in peach. BMC Plant Biol 11: 107. 21679395 10.1186/1471-2229-11-107 PMC3141638

[23] An, Yang J, Zhang P (2012) Transcriptome profiling of low temperature treated cassava apical shoots showed dynamic responses of tropical plant to cold stress. BMC Genomics 13: 64. 22321773 10.1186/1471-2164-13-64 PMC3339519

[24] Bolstad BM, Irizarry RA, Astrand M, Speed TP (2003) A comparison of normalization methods for high density oligonucleotide array data based on variance and bias. Bioinformatics 19: 185–193. 12538238 10.1093/bioinformatics/19.2.185

[25] Irizarry RA, Hobbs B, Collin F, Beazer-Barclay YD, Antonellis KJ, et al (2003) Exploration, normalization, and summaries of high density oligonucleotide array probe level data. Biostatistics 4(2): 249–264. 12925520 10.1093/biostatistics/4.2.249

[26] Unver T, Budak H (2009) Conserved microRNAs and their targets in model grass species *Brachypodium distachyon* . Planta 230: 659–669. 19585143 10.1007/s00425-009-0974-7

[27] Untergasser A, Cutcutache I, Koressaar T, Ye J, Faircloth BC, et al (2012) Primer3-new capabilities and interfaces. Nucleic Acids Res 40(15): e115. 22730293 10.1093/nar/gks596 PMC3424584

[28] Unver T, Parmaksız I, Dündar E (2010a) Identification of conserved micro-RNAs and their target transcripts in opium poppy (*Papaver somniferum* L.). Plant Cell Rep 29: 757–769. 20443006 10.1007/s00299-010-0862-4

[29] Eldem V, Çelikkol Akçay U, Ozhuner E, Bakır Y, Uranbey S, et al (2012) Genome-Wide Identification of miRNAs Responsive to Drought in Peach (*Prunus persica*) by High-Throughput Deep Sequencing. PLoS ONE 7(12): e50298 doi:10.1371/journal.pone.0050298 23227166 10.1371/journal.pone.0050298 PMC3515591

[30] Yanik H, Türktaş M, Dundar E, Hernandez P, Dorado G et al. (2013) Genome-wide identification of alternate bearing-associated miRNA in the olive tree (*Olea europaea)*. BMC Plant Biology doi:10.1186/1471-2229-13-1. 10.1186/1471-2229-13-10 PMC3564680 23320600

[31] Weber M, Harada E, Vess C, von Roepenack-Lahaye E, Clemens S (2004) Comparative microarray analysis of Arabidopsis thaliana and Arabidopsis halleri roots identifies nicotianamine synthase, a ZIP transporter and other genes as potential metal hyperaccumulation factors. Plant Journal 37(2): 269–281. 14690510 10.1046/j.1365-313x.2003.01960.x

[32] Moore S, Payton P, Wright M, Tanksley S, Giovannoni J (2005) Utilization of tomato microarrays for comparative gene expression analysis in the Solanaceae. Journal of Experimental Botany 56(421): 2885–2895. 16216847 10.1093/jxb/eri283

[33] Davey M, Graham N, Vanholme B, Swennen R, May S, et al (2009) Heterologous oligonucleotide microarrays for transcriptomics in a non-model species; a proof-of-concept study of drought stress in Musa. BMC Genomics 10: 436. 19758430 10.1186/1471-2164-10-436 PMC2761422

[34] Parikh A, Miranda ER, Katoh-Kurasawa M, Fuller D, Rot G, Zagar L, Curk T, Sucgang R, Chen R, Zupan B, Loomis WF, Kuspa A, Shaulsky G (2010) Conserved developmental transcriptomes in evolutionarily divergent species. Genome Biology 11: R35. 20236529 10.1186/gb-2010-11-3-r35 PMC2864575

[35] Alagna F, Nunzio D’Agostino N, Torchia L, Servili M, Rao R, et al (2009) Comparative 454 pyrosequencing of transcripts from two olive genotypes during fruit development. BMC Genomics 10: 399. 19709400 10.1186/1471-2164-10-399 PMC2748093

[36] Goldschmidt EE, Golomb A (1982) Girdling affects carbohydrate related gene expression in leaves bark and roots of alternate bearing citrus trees. Ann Bot - London 107(2): 206–208. 10.1093/aob/mcg108 PMC4243633 12763756

[37] Spann TM, Beede RH, Dejong TM (2008) Seasonal carbohydrate storage and mobilization in bearing and non-bearing pistachio (*Pistacia vera*) trees. Tree Physiol 28: 207–213. 18055431 10.1093/treephys/28.2.207

[38] Bustan A, Avni A, Lavee S, Zipori I, Yeselson Y, et al (2011) Role of carbohydrate reserves in yield production of intensively cultivated oil olive (*Olea europaea* L.) trees. Tree Physiol 31: 519–530. 21571726 10.1093/treephys/tpr036

[39] Stutte G, Martin GC (1986) Effect of light intensity and carbohydrate reserves on flowering in olive. J Amer Soc Hort Sci 11: 27–31.

[40] De la Rosa, R, Rallo L, Rapoport HF (2000) Olive floral bud growth and starch content during winter rest and spring budbreak. Hort Science 35: 1223–1227.

[41] Bouranis DL, Kitsaki CK, Chorianopoulou SN, Aivalakis G, Drossopoulos JB (1999) Nutritional dynamics of olive tree flowers. J Plant Nutr 22(2): 245–257.

[42] Erel R, Dag A, Ben-Gal A, Schwarts A, Yermiyahu U (2008) Flowering and fruit set of olive trees in response to nitrogen, phosphorus, and potassium. J Amer Soc Hort Sci 133(5): 639–647.

[43] Fernândez-Escobar R, Moreno R, Garcia-Creus M (1999) Seasonal changes of mineral nutrients in olive leaves during the alternate-bearing cycle. Sci Hortic - Amsterdam 82: 25–45.

[44] Rowland LJ, Alkharouf N, Darwish O, Ogden EL, Polashock JJ, et al (2012) Generation and analysis of blueberry transcriptome sequences from leaves, developing fruit, and flower buds from cold acclimation through deacclimation. BMC Plant Biol 12: 46. 22471859 10.1186/1471-2229-12-46 PMC3378433

[45] Pignocchi C, Kiddle G, Hernández I, Foster SJ, Asensi A, et al (2006) Ascorbate oxidase-dependent changes in the redox state of the apoplast modulate gene transcript accumulation leading to modified hormone signaling and orchestration of defense processes in tobacco. Plant Physiol 141: 423–435. 16603663 10.1104/pp.106.078469 PMC1475448

[46] Bonzon M, Simon P, Greppin H, Wagner E (1983) Pyridine-nucleotides and redox charge evolution during the induction of flowering in spinach leaves. Planta 159: 254–260. 24258176 10.1007/BF00397533

[47] Foreman J, Demidchik V, Bothwell JHF, Mylona P, Miedema H, et al (2003) Reactive oxygen species produced by NADPH oxidase regulate plant cell growth. Nature 422: 442–446. 12660786 10.1038/nature01485

[48] Shalom L, Samuels S, Zur N, Shlizerman L, Zemach H, et al (2012) Alternate bearing in citrus: changes in the expression of flowering control genes and in global gene expression in on- versus off-crop trees. PLoS ONE 7(10): e46930 doi:10.1371/journal.pone.0046930 23071667 10.1371/journal.pone.0046930 PMC3469648

[49] Goldschmidt EE (1999) Carbohydrate supply as a critical factor for citrus fruit development and productivity. Hortscience 34: 1020–1024.

[50] Ramel F, Sulmon C, Gouesbet G, Couée I (2009) Natural variation reveals relationships between pre-stress carbohydrate nutritional status and subsequent responses to xenobiotic and oxidative stress in *Arabidopsis thaliana* . Ann Bot - London 104: 1323–1337. 10.1093/aob/mcp243 PMC2778391 19789177

[51] Grace SC, Logan BA (2000) Energy dissipation and radical scavenging by the plant phenylpropanoid pathway. Phil Trans Royal Soc London, Series B-Biol Sci 355: 1499–1510. 10.1098/rstb.2000.0710 PMC1692864 11128003

[52] Lavee S, Rallo L, Rapoport HF, Troncoso A (1996) The floral biology of the olive: effect of flower number, type and distribution on fruitset. Sci Hortic-Amsterdam 66: 149–158.

[53] Muñoz-Fambuena N, Mesejo S, Gonźales-Mas MC, Primo-Millo E, Agustí M, et al (2011) Fruit regulates seasonal expression of flowering genes in alternate-bearing ‘Moncada’ mandarin. Ann Bot-London 108: 511–519. 10.1093/aob/mcr164 PMC3158683 21856639

[54] Ulger S, Baktır İ, Kaynak L (1999) Zeytinlerde periyodisite ve çiçek tomurcuğu oluşumu üzerine içsel büyüme hormonlarının etkilerinin saptanması. Turk J Agric For 23(3): 619–623 (in Tr)..

[55] Jones WW, Coggins CW, Embleton TW (1976) Endogenous abscisic acid in relation to bud growth in alternate bearing ‘Valencia’ orange. Plant Physiol 58: 681–682. 16659743 10.1104/pp.58.5.681 PMC542282

[56] Al-Shdiefat SM, Qrunfleh MM (2008) Alternate bearing of the olive (*Olea europaea* L.) as related to endogenous hormonal content. JJAS 4(1): 12–25.

[57] Lavee S (1986) Olive P: 261–276 In: Monselise SP (ed). CRC handbook of fruit set and development. CRC Press, Boca Raton, Fla.

