# Supplementary material for: Nutrition Metabolism Plays an Important Role in the Alternate Bearing of the Olive Tree (Olea europaea L.)
Source: PLoS One. 2013 Mar 28;8(3):e59876. doi: 10.1371/journal.pone.0059876 (PMC3610735; doi:10.1371/journal.pone.0059876)
Supplement: Table S2 — Differentially expressed transcripts between juvenile and mature leaves. Fold changes were given in log2-based numbers. (−) indicates down-regulation. (DOCX) [file pone.0059876.s002.docx]

**Table S1.** Differentially expressed transcripts between juvenile and mature leaves. Fold changes were given in log2-based numbers. (-) indicates down-regulation.

| **Seq_ID** | **Description** | **ON-M /ON-J-fold change** | ***P* value** | **GO biological process** |
| --- | --- | --- | --- | --- |
| FL684392 | FL684392 A_K15_F08_0414F_p1 *Olea europaea* cv. Leccino fruitlet *Olea europaea* cDNA | –6.42 | 0.00 | Unknown |
| FL683857 | FL683857 A_C23_B12_0414C_p1 *Olea europaea* cv. Leccino fruitlet *Olea europaea* cDNA | –6.15 | 0.00 | Cellular amino acid metabolic process |
| GO245805 | GO245805 OEAA-070810_Plate8e24.b1 cDNA library from Olive leaves and fruits *Olea europaea* cDNA | –6.03 | 0.00 | Cellular amino acid metabolic process |
| FL683748 | FL683748 A_M22_G11_0414F_p2 *Olea europaea* cv. Leccino fruitlet *Olea europaea* cDNA | –5.91 | 0.00 | Cellular amino acid metabolic process |
| FL683833 | FL683833 A_K01_F01_0414C_p1 *Olea europaea* cv. Leccino fruitlet *Olea europaea* cDNA | –5.85 | 0.00 | Cellular amino acid metabolic process |
| GO245304 | GO245304 OEAA-070810_Plate6p15.b1 cDNA library from Olive leaves and fruits *Olea europaea* cDNA | –5.35 | 0.00 | Response to karrikin, syncytium formation |
| GO243808 | GO243808 OEAA-070810_Plate2p13.b1 cDNA library from Olive leaves and fruits *Olea europaea* cDNA | –5.23 | 0.00 | Cellular amino acid metabolic process |
| FL684126 | FL684126 D_B23_A12_0414F_p11 *Olea europaea* cv. Leccino fruitlet *Olea europaea* cDNA | –4.76 | 0.00 | Flavonoid biosynthetic process |
| GO245492 | GO245492 OEAA-070810_Plate7h17.b1 cDNA library from Olive leaves and fruits *Olea europaea* cDNA | –4.72 | 0.00 | Unknown |
| GO243777 | GO243777 OEAA-070810_Plate2o06.b1 cDNA library from Olive leaves and fruits *Olea europaea* cDNA | –4.68 | 0.00 | Type I hypersensitivity |
| GO243932 | GO243932 OEAA-070810_Plate3e19.b1 cDNA library from Olive leaves and fruits *Olea europaea* cDNA | –4.66 | 0.00 | Unknown |
| FL684185 | FL684185 B_N02_G01_0414F_p4 *Olea europaea* cv. Leccino fruitlet *Olea europaea* cDNA | –4.58 | 0.00 | Nucleoside metabolic process, protein ubiquitination |
| GO243169 | GO243169 OEAA-070810_Plate1d20.b1 cDNA library from Olive leaves and fruits *Olea europaea* cDNA | –4.56 | 0.00 | Unknown |
| GO244883 | GO244883 OEAA-070810_Plate5n13.b1 cDNA library from Olive leaves and fruits *Olea europaea* cDNA | –4.49 | 0.00 | Fatty acid metabolic process, oxidation-reduction process |
| GO243710 | GO243710 OEAA-070810_Plate2l05.b1 cDNA library from Olive leaves and fruits *Olea europaea* cDNA | –4.48 | 0.00 | Metabolic process |
| GO245050 | GO245050 OEAA-070810_Plate6e17.b1 cDNA library from Olive leaves and fruits *Olea europaea* cDNA | –4.36 | 0.00 | Cellular amino acid metabolic process |
| FL683725 | FL683725 A_I08_E04_0414F_p2 *Olea europaea* cv. Leccino fruitlet *Olea europaea* cDNA | –4.19 | 0.00 | Chlorophyll biosynthetic process, terpenoid biosynthetic process, |
| FL684170 | FL684170 B_K11_F06_0414F_p5 *Olea europaea* cv. Leccino fruitlet *Olea europaea* cDNA | –4.14 | 0.01 | defense response to bacterium, protein folding, response to arsenic-containing substance, response to heat |
| GO243394 | GO243394 OEAA-070810_Plate1n12.b1 cDNA library from Olive leaves and fruits *Olea europaea* cDNA | –4.13 | 0.00 | Unknown |
| FL683395 | FL683395 C_O09_H05_0414F_p9 *Olea europaea* cv. Leccino fruitlet *Olea europaea* cDNA | –4.04 | 0.00 | Unknown |
| GO244575 | GO244575 OEAA-070810_Plate5a01.b1 cDNA library from Olive leaves and fruits *Olea europaea* cDNA | –3.99 | 0.00 | Unknown |
| FL684327 | FL684327 B_K02_F01_0414C_p2 *Olea europaea* cv. Leccino fruitlet *Olea europaea* cDNA | –3.91 | 0.00 | Unknown |
| GO243610 | GO243610 OEAA-070810_Plate2g19.b1 cDNA library from Olive leaves and fruits *Olea europaea* cDNA | –3.88 | 0.00 | Methionine biosynthetic process, regulation of meristem growth, proteolysis, negative regulation of catalytic activity, RNA splicing, via endonucleolytic cleavage and ligation, anther development, plant-type cell wall organization, plant-type cell wall biogenesis |
| FN998508 | FN998508 FN998508 *Olea europaea* flower *Olea europaea* cDNA clone c2-3-D2 | –3.88 | 0.00 | Mucilage metabolic process involved seed coat development, mucilage extrusion from seed coat, proteolysis, negative regulation of catalytic activity |
| GO245613 | GO245613 OEAA-070810_Plate7m21.b1 cDNA library from Olive leaves and fruits *Olea europaea* cDNA | –3.86 | 0.00 | Metabolic process |
| GO244854 | GO244854 OEAA-070810_Plate5m07.b1 cDNA library from Olive leaves and fruits *Olea europaea* cDNA | –3.85 | 0.00 | Urea transmembrane transport, water transport |
| GO243938 | GO243938 OEAA-070810_Plate3f01.b1 cDNA library from Olive leaves and fruits *Olea europaea* cDNA | –3.85 | 0.00 | Unknown |
| GO243399 | GO243399 OEAA-070810_Plate1n17.b1 cDNA library from Olive leaves and fruits *Olea europaea* cDNA | –3.81 | 0.00 | Biosynthetic process |
| GO244502 | GO244502 OEAA-070810_Plate4m21.b1 cDNA library from Olive leaves and fruits *Olea europaea* cDNA | –3.81 | 0.00 | Cellular amino acid metabolic process |
| FN998379 | FN998379 FN998379 *Olea europaea* flower *Olea europaea* cDNA clone c2-1-G9 | –3.79 | 0.01 | Mucilage metabolic process involved seed coat development, mucilage extrusion from seed coat, proteolysis, negative regulation of catalytic activity |
| GO243240 | GO243240 OEAA-070810_Plate1g20.b1 cDNA library from Olive leaves and fruits *Olea europaea* cDNA | –3.78 | 0.00 | Lipid metabolic process |
| GO243557 | GO243557 OEAA-070810_Plate2e11.b1 cDNA library from Olive leaves and fruits *Olea europaea* cDNA | –3.77 | 0.00 | Lipid transport, megagametogenesis, transition metal ion transport |
| GO245635 | GO245635 OEAA-070810_Plate7n19.b1 cDNA library from Olive leaves and fruits *Olea europaea* cDNA | –3.71 | 0.00 | Unknown |
| GO245023 | GO245023 OEAA-070810_Plate6d14.b1 cDNA library from Olive leaves and fruits *Olea europaea* cDNA | –3.69 | 0.00 | ATP catabolic process, cuticle development, drug transmembrane transport |
| GO243156 | GO243156 OEAA-070810_Plate1d03.b1 cDNA library from Olive leaves and fruits *Olea europaea* cDNA | –3.69 | 0.00 | Response to biotic stimulus, defense response |
| GO244287 | GO244287 OEAA-070810_Plate4d17.b1 cDNA library from Olive leaves and fruits *Olea europaea* cDNA | –3.68 | 0.00 | Plant-type cell wall organization |
| GO245554 | GO245554 OEAA-070810_Plate7k08.b1 cDNA library from Olive leaves and fruits *Olea europaea* cDNA | –3.65 | 0.00 | Unknown |
| GO244599 | GO244599 OEAA-070810_Plate5b03.b1 cDNA library from Olive leaves and fruits *Olea europaea* cDNA | –3.61 | 0.00 | Urea transmembrane transport, water transport |
| GO243712 | GO243712 OEAA-070810_Plate2l07.b1 cDNA library from Olive leaves and fruits *Olea europaea* cDNA | –3.60 | 0.00 | Plant-type cell wall organization |
| GO243532 | GO243532 OEAA-070810_Plate2d10.b1 cDNA library from Olive leaves and fruits *Olea europaea* cDNA | –3.60 | 0.00 | Cellulose biosynthetic process, cellular cell wall organization |
| GO243405 | GO243405 OEAA-070810_Plate1n23.b1 cDNA library from Olive leaves and fruits *Olea europaea* cDNA | –3.59 | 0.00 | Unknown |
| GO244526 | GO244526 OEAA-070810_Plate4n23.b1 cDNA library from Olive leaves and fruits *Olea europaea* cDNA | –3.57 | 0.00 | Unknown |
| GO243415 | GO243415 OEAA-070810_Plate1o09.b1 cDNA library from Olive leaves and fruits *Olea europaea* cDNA | –3.55 | 0.00 | Unknown |
| FL684367 | FL684367 C_C09_B05_0414F_p9 *Olea europaea* cv. Leccino fruitlet *Olea europaea* cDNA | –3.53 | 0.00 | Unknown |
| GO243457 | GO243457 OEAA-070810_Plate2a04.b1 cDNA library from Olive leaves and fruits *Olea europaea* cDNA | –3.52 | 0.00 | Unknown |
| GO245680 | GO245680 OEAA-070810_Plate7p16.b1 cDNA library from Olive leaves and fruits *Olea europaea* cDNA | –3.49 | 0.00 | Water transport, transmembrane transport, response to abscisic acid stimulus |
| FL684184 | FL684184 C_D12_B06_0414F_p8 *Olea europaea* cv. Leccino fruitlet *Olea europaea* cDNA | –3.47 | 0.00 | Response to endoplasmic reticulum stress, protein folding, heat acclimation, protein unfolding, response to high light intensity, response to hydrogen peroxide |
| GO245394 | GO245394 OEAA-070810_Plate7d11.b1 cDNA library from Olive leaves and fruits *Olea europaea* cDNA | –3.45 | 0.00 | Unknown |
| GO244165 | GO244165 OEAA-070810_Plate3o13.b1 cDNA library from Olive leaves and fruits *Olea europaea* cDNA | –3.43 | 0.00 | Response to abscisic acid stimulus, transmembrane transport, water transport |
| FL683438 | FL683438 D_K16_F08_0414F_p14 *Olea europaea* cv. Leccino fruitlet *Olea europaea* cDNA | –3.43 | 0.00 | Unknown |
| GO243211 | GO243211 OEAA-070810_Plate1f15.b1 cDNA library from Olive leaves and fruits *Olea europaea* cDNA | –3.41 | 0.00 | Defense response, response to biotic stimulus, |
| GO243219 | GO243219 OEAA-070810_Plate1f23.b1 cDNA library from Olive leaves and fruits *Olea europaea* cDNA | –3.41 | 0.01 | Unknown |
| FL684339 | FL684339 B_K12_F06_0414C_p2 *Olea europaea* cv. Leccino fruitlet *Olea europaea* cDNA | –3.39 | 0.00 | Unknown |
| GO243814 | GO243814 OEAA-070810_Plate2p19.b1 cDNA library from Olive leaves and fruits *Olea europaea* cDNA | –3.37 | 0.00 | Defense response, response to biotic stimulus, |
| GO243991 | GO243991 OEAA-070810_Plate3h07.b1 cDNA library from Olive leaves and fruits *Olea europaea* cDNA | –3.35 | 0.00 | Fatty acid metabolic process, oxidation-reduction process |
| GO243798 | GO243798 OEAA-070810_Plate2p03.b1 cDNA library from Olive leaves and fruits *Olea europaea* cDNA | –3.34 | 0.00 | Fatty acid biosynthetic process, response to cold, very long-chain fatty acid metabolic process, cuticle development, response to light stimulus, response to karrikin, epidermal cell differentiation |
| GO243147 | GO243147 OEAA-070810_Plate1c18.b1 cDNA library from Olive leaves and fruits *Olea europaea* cDNA | –3.33 | 0.00 | Wax biosynthetic process, cutin biosynthetic process, lateral root formation, defense response to insect, long-chain fatty acid metabolic process, defense response to fungus |
| GO244025 | GO244025 OEAA-070810_Plate3i17.b1 cDNA library from Olive leaves and fruits *Olea europaea* cDNA | –3.31 | 0.01 | Response to cadmium ion, protein folding, response to high light intensity, response to hydrogen peroxide, response to endoplasmic reticulum stress, response to heat |
| GO243114 | GO243114 OEAA-070810_Plate1b09.b1 cDNA library from Olive leaves and fruits *Olea europaea* cDNA | –3.31 | 0.00 | Flavonol biosynthetic process, oxidation-reduction process, response to karrikin, response to light stimulus, |
| GO243362 | GO243362 OEAA-070810_Plate1m04.b1 cDNA library from Olive leaves and fruits *Olea europaea* cDNA | –3.31 | 0.00 | ATP catabolic process, wax biosynthetic process, response to salt stress, response to abscisic acid stimulus, response to karrikin |
| GO245612 | GO245612 OEAA-070810_Plate7m20.b1 cDNA library from Olive leaves and fruits *Olea europaea* cDNA | –3.29 | 0.00 | Unknown |
| GO244094 | GO244094 OEAA-070810_Plate3l14.b1 cDNA library from Olive leaves and fruits *Olea europaea* cDNA | –3.24 | 0.00 | Fatty acid metabolic process, oxidation-reduction process, |
| GO244351 | GO244351 OEAA-070810_Plate4g09.b1 cDNA library from Olive leaves and fruits *Olea europaea* cDNA | –3.21 | 0.00 | Metabolic process |
| GO245563 | GO245563 OEAA-070810_Plate7k17.b1 cDNA library from Olive leaves and fruits *Olea europaea* cDNA | –3.21 | 0.00 | Unknown |
| GO246183 | GO246183 OEAA-070810_Plate9f01.b1 cDNA library from Olive leaves and fruits *Olea europaea* cDNA | –3.20 | 0.03 | Unknown |
| FL684349 | FL684349 B_A04_A02_0414C_p2 *Olea europaea* cv. Leccino fruitlet *Olea europaea* cDNA | –3.20 | 0.00 | Unknown |
| GO244400 | GO244400 OEAA-070810_Plate4i11.b1 cDNA library from Olive leaves and fruits *Olea europaea* cDNA | –3.16 | 0.00 | Unknown |
| GO245628 | GO245628 OEAA-070810_Plate7n12.b1 cDNA library from Olive leaves and fruits *Olea europaea* cDNA | –3.15 | 0.00 | Response to stress |
| GO245020 | GO245020 OEAA-070810_Plate6d11.b1 cDNA library from Olive leaves and fruits *Olea europaea* cDNA | –3.14 | 0.00 | Unknown |
| GO244894 | GO244894 OEAA-070810_Plate5n24.b1 cDNA library from Olive leaves and fruits *Olea europaea* cDNA | –3.12 | 0.00 | Lipid metabolic process |
| GO246421 | GO246421 OEAA-070810_Plate9p09.b1 cDNA library from Olive leaves and fruits *Olea europaea* cDNA | –3.09 | 0.00 | Defense response to fungus, incompatible interaction, salicylic acid metabolic process, systemic acquired resistance, salicylic acid mediated signaling pathway |
| FL684364 | FL684364 A_H23_D12_0414F_p3 *Olea europaea* cv. Leccino fruitlet *Olea europaea* cDNA | –3.08 | 0.01 | Unknown |
| FL683700 | FL683700 B_B22_A11_0414F_p4 *Olea europaea* cv. Leccino fruitlet *Olea europaea* cDNA | –3.07 | 0.00 | Response to water deprivation, protein folding, response to salt stress, heat acclimation, flower development, protein stabilization, cellular response to calcium ion, leaf development, response to arsenic-containing substance, response to high light intensity, response to hydrogen peroxide, defense response to bacterium, incompatible interaction |
| GO244424 | GO244424 OEAA-070810_Plate4j13.b1 cDNA library from Olive leaves and fruits *Olea europaea* cDNA | –3.05 | 0.00 | Response to sucrose stimulus, response to red light, nonphotochemical quenching, rRNA processing, response to blue light, regulation of proton transport, response to far red light, photosynthesis, light harvesting, response to high light intensity, cysteine biosynthetic process |
| GO244188 | GO244188 OEAA-070810_Plate3p13.b1 cDNA library from Olive leaves and fruits *Olea europaea* cDNA | –3.04 | 0.00 | Oxidation-reduction process |
| GO244677 | GO244677 OEAA-070810_Plate5e13.b1 cDNA library from Olive leaves and fruits *Olea europaea* cDNA | –3.03 | 0.00 | Response to wounding, steroid metabolic process, xylem and phloem pattern formation, |
| GO246070 | GO246070 OEAA-070810_Plate9a08.b1 cDNA library from Olive leaves and fruits *Olea europaea* cDNA | –3.01 | 0.00 | Unknown |
| GO244678 | GO244678 OEAA-070810_Plate5e14.b1 cDNA library from Olive leaves and fruits *Olea europaea* cDNA | –3.01 | 0.00 | Response to biotic stimulus, defense response |
| FL684306 | FL684306 C_C01_B01_0414F_p9 *Olea europaea* cv. Leccino fruitlet *Olea europaea* cDNA | –2.99 | 0.00 | Response to water deprivation, protein folding, response to salt stress, heat acclimation, flower development, protein stabilization, cellular response to calcium ion, leaf development, response to arsenic-containing substance, response to high light intensity, response to hydrogen peroxide, defense response to bacterium, incompatible interaction |
| GO244836 | GO244836 OEAA-070810_Plate5l12.b1 cDNA library from Olive leaves and fruits *Olea europaea* cDNA | –2.99 | 0.00 | Unknown |
| GO244999 | GO244999 OEAA-070810_Plate6c13.b1 cDNA library from Olive leaves and fruits *Olea europaea* cDNA | –2.99 | 0.00 | Unknown |
| GO244699 | GO244699 OEAA-070810_Plate5f12.b1 cDNA library from Olive leaves and fruits *Olea europaea* cDNA | –2.99 | 0.00 | Tetracyclic triterpenoid biosynthetic process, pentacyclic triterpenoid biosynthetic process, tricyclic triterpenoid biosynthetic process, thalianol metabolic process, root development |
| FL683508 | FL683508 A_A21_A11_0414C_p1 *Olea europaea* cv. Leccino fruitlet *Olea europaea* cDNA | –2.98 | 0.01 | Oxidation-reduction process, response to oxidative stress |
| GO243079 | GO243079 OEAA-070810_Plate10p19.b1 cDNA library from Olive leaves and fruits *Olea europaea* cDNA | –2.98 | 0.00 | Transmembrane transport, response to abiotic stimulus, monovalent inorganic cation transport, response to chemical stimulus, calcium ion transport, cellular divalent inorganic cation homeostasis, response to stress |
| GO243927 | GO243927 OEAA-070810_Plate3e14.b1 cDNA library from Olive leaves and fruits *Olea europaea* cDNA | –2.97 | 0.00 | Oxidation-reduction process |
| GO243842 | GO243842 OEAA-070810_Plate3b01.b1 cDNA library from Olive leaves and fruits *Olea europaea* cDNA | –2.97 | 0.00 | Amine metabolic process, oxidation-reduction process, |
| FL684199 | FL684199 A_A01_A01_0414F_p1 *Olea europaea* cv. Leccino fruitlet *Olea europaea* cDNA | –2.96 | 0.00 | Oxidation-reduction process, response to oxidative stress |
| GO243358 | GO243358 OEAA-070810_Plate1l24.b1 cDNA library from Olive leaves and fruits *Olea europaea* cDNA | –2.96 | 0.00 | Amine metabolic process, oxidation-reduction process, |
| FN998464 | FN998464 FN998464 *Olea europaea* flower *Olea europaea* cDNA clone c2-2-H3 | –2.96 | 0.00 | Wax biosynthetic process, cutin biosynthetic process, lateral root formation, defense response to insect, long-chain fatty acid metabolic process, defense response to fungus |
| GO244013 | GO244013 OEAA-070810_Plate3i05.b1 cDNA library from Olive leaves and fruits *Olea europaea* cDNA | –2.95 | 0.00 | Unknown |
| GO243043 | GO243043 OEAA-070810_Plate10o07.b1 cDNA library from Olive leaves and fruits *Olea europaea* cDNA | –2.95 | 0.00 | Calcium ion transport, cellular divalent inorganic cation homeostasis, monovalent inorganic cation transport, response to abiotic stimulus, response to chemical stimulus, response to stres, transmembrane transport, |
| GO243848 | GO243848 OEAA-070810_Plate3b07.b1 cDNA library from Olive leaves and fruits *Olea europaea* cDNA | –2.95 | 0.00 | Type I hypersensitivity |
| GO245459 | GO245459 OEAA-070810_Plate7g07.b1 cDNA library from Olive leaves and fruits *Olea europaea* cDNA | –2.94 | 0.01 | Unknown |
| GO243678 | GO243678 OEAA-070810_Plate2j17.b1 cDNA library from Olive leaves and fruits *Olea europaea* cDNA | –2.94 | 0.00 | Embryo development ending in seed dormancy, epidermis morphogenesis, cutin biosynthetic process, localization, trichome morphogenesis |
| GO243553 | GO243553 OEAA-070810_Plate2e07.b1 cDNA library from Olive leaves and fruits *Olea europaea* cDNA | –2.93 | 0.00 | Anthocyanin biosynthetic process, response to karrikin, response to UV-B, |
| GO243363 | GO243363 OEAA-070810_Plate1m05.b1 cDNA library from Olive leaves and fruits *Olea europaea* cDNA | –2.93 | 0.00 | Fatty acid metabolic process, oxidation-reduction process, |
| GO245436 | GO245436 OEAA-070810_Plate7f06.b1 cDNA library from Olive leaves and fruits *Olea europaea* cDNA | –2.91 | 0.00 | Unknown |
| GO243323 | GO243323 OEAA-070810_Plate1k12.b1 cDNA library from Olive leaves and fruits *Olea europaea* cDNA | –2.91 | 0.00 | Unknown |
| GO244858 | GO244858 OEAA-070810_Plate5m11.b1 cDNA library from Olive leaves and fruits *Olea europaea* cDNA | –2.91 | 0.00 | Unknown |
| GO243940 | GO243940 OEAA-070810_Plate3f03.b1 cDNA library from Olive leaves and fruits *Olea europaea* cDNA | –2.91 | 0.00 | Unknown |
| GO243402 | GO243402 OEAA-070810_Plate1n20.b1 cDNA library from Olive leaves and fruits *Olea europaea* cDNA | –2.91 | 0.00 | Unknown |
| FL683688 | FL683688 A_P09_H05_0414F_p3 *Olea europaea* cv. Leccino fruitlet *Olea europaea* cDNA | –2.90 | 0.01 | Oxidation-reduction process, response to oxidative stress |
| GO243584 | GO243584 OEAA-070810_Plate2f16.b1 cDNA library from Olive leaves and fruits *Olea europaea* cDNA | –2.90 | 0.00 | Wax biosynthetic process, response to salt stress, ATP catabolic process, fatty acid catabolic process, response to abscisic acid stimulus, response to karrikin |
| GO245690 | GO245690 OEAA-070810_Plate8a02.b1 cDNA library from Olive leaves and fruits *Olea europaea* cDNA | –2.90 | 0.00 | Water transport, transmembrane transport, response to abscisic acid stimulus |
| FL684377 | FL684377 B_M06_G03_0414F_p6 *Olea europaea* cv. Leccino fruitlet *Olea europaea* cDNA | –2.90 | 0.00 | Unknown |
| GO245128 | GO245128 OEAA-070810_Plate6i01.b1 cDNA library from Olive leaves and fruits *Olea europaea* cDNA | –2.88 | 0.00 | Response to water deprivation, response to cold, response to salt stress, oxidation-reduction process |
| GO243302 | GO243302 OEAA-070810_Plate1j15.b1 cDNA library from Olive leaves and fruits *Olea europaea* cDNA | –2.87 | 0.00 | Unknown |
| FL683923 | FL683923 A_N05_G03_0414F_p3 *Olea europaea* cv. Leccino fruitlet *Olea europaea* cDNA | –2.86 | 0.01 | Oxidation-reduction process, response to oxidative stress |
| GO246041 | GO246041 OEAA-070810_Plate8p02.b1 cDNA library from Olive leaves and fruits *Olea europaea* cDNA | –2.84 | 0.01 | N-terminal protein myristoylation, regulation of ATPase activity, protein folding, response to hydrogen peroxide, positive regulation of flower development, response to salt stress, response to high light intensity, response to heat |
| GO245033 | GO245033 OEAA-070810_Plate6d24.b1 cDNA library from Olive leaves and fruits *Olea europaea* cDNA | –2.82 | 0.01 | Fatty acid metabolic process, oxidation-reduction process |
| GO244875 | GO244875 OEAA-070810_Plate5n05.b1 cDNA library from Olive leaves and fruits *Olea europaea* cDNA | –2.82 | 0.00 | Unknown |
| GO245373 | GO245373 OEAA-070810_Plate7c14.b1 cDNA library from Olive leaves and fruits *Olea europaea* cDNA | –2.80 | 0.00 | Calcium ion transport, cellular divalent inorganic cation homeostasis, monovalent inorganic cation transport, response to abiotic stimulus, response to chemical stimulus, response to stres, transmembrane transport, |
| GO245362 | GO245362 OEAA-070810_Plate7c03.b1 cDNA library from Olive leaves and fruits *Olea europaea* cDNA | –2.79 | 0.00 | Terpenoid biosynthetic process |
| FL683840 | FL683840 A_G24_D12_0414F_p2 *Olea europaea* cv. Leccino fruitlet *Olea europaea* cDNA | –2.79 | 0.00 | Oxidation-reduction process, response to oxidative stress |
| GO245184 | GO245184 OEAA-070810_Plate6k10.b1 cDNA library from Olive leaves and fruits *Olea europaea* cDNA | –2.78 | 0.01 | Lipid metabolic process |
| GO245186 | GO245186 OEAA-070810_Plate6k12.b1 cDNA library from Olive leaves and fruits *Olea europaea* cDNA | –2.77 | 0.01 | MAPK cascade |
| FL683903 | FL683903 A_M05_G03_0414F_p1 *Olea europaea* cv. Leccino fruitlet *Olea europaea* cDNA | –2.76 | 0.01 | Defense response to bacterium, secondary cell wall biogenesis, defense response to fungus, cellulose biosynthetic process, cellular cell wall organization |
| FL684040 | FL684040 B_A08_A04_0414F_p6 *Olea europaea* cv. Leccino fruitlet *Olea europaea* cDNA | –2.76 | 0.00 | Unknown |
| GO243430 | GO243430 OEAA-070810_Plate1o24.b1 cDNA library from Olive leaves and fruits *Olea europaea* cDNA | –2.76 | 0.00 | Unknown |
| GO244454 | GO244454 OEAA-070810_Plate4k20.b1 cDNA library from Olive leaves and fruits *Olea europaea* cDNA | –2.76 | 0.00 | Unknown |
| FL683494 | FL683494 D_F02_C01_0414F_p12 *Olea europaea* cv. Leccino fruitlet *Olea europaea* cDNA | –2.75 | 0.01 | Unknown |
| FL683673 | FL683673 A_O06_H03_0414F_p2 *Olea europaea* cv. Leccino fruitlet *Olea europaea* cDNA | –2.74 | 0.01 | Unknown |
| GO244160 | GO244160 OEAA-070810_Plate3o08.b1 cDNA library from Olive leaves and fruits *Olea europaea* cDNA | –2.73 | 0.01 | Protein folding, response to high light intensity, response to arsenic-containing substance, response to hydrogen peroxide, response to heat, defense response to bacterium, incompatible interaction |
| GO245996 | GO245996 OEAA-070810_Plate8n03.b1 cDNA library from Olive leaves and fruits *Olea europaea* cDNA | –2.72 | 0.00 | Unknown |
| GO243145 | GO243145 OEAA-070810_Plate1c16.b1 cDNA library from Olive leaves and fruits *Olea europaea* cDNA | –2.72 | 0.00 | Metabolic process |
| GO244493 | GO244493 OEAA-070810_Plate4m12.b1 cDNA library from Olive leaves and fruits *Olea europaea* cDNA | –2.71 | 0.00 | Unknown |
| eugene3.00101184 | CER6, G2, POP1, CUT1 \| CUT1 (CUTICULAR 1); acyltransferase | –2.71 | 0.02 | Fatty acid biosynthetic process, wax biosynthetic process, response to llight stimulus, cell tip growth, response to far red light, chrophyll metabolic process, response to UV light, very-long chain fatty acid metabolic process, response to high light intensity, aromatic amino acid family metabolic process, oxidoreduction coenzyme metabolic process, rsponse to cold, embryo development ending in seed dormancy, ovule development, jasmonic acid biosynthetic process, regulation of hormone levels |
| gw1.III.2548.1 | protein kinase family protein | –2.71 | 0.01 | Protein phosphorylation, transferase activity |
| GO246127 | GO246127 OEAA-070810_Plate9c17.b1 cDNA library from Olive leaves and fruits *Olea europaea* cDNA | –2.70 | 0.00 | Unknown |
| FL683771 | FL683771 B_E04_C02_0414F_p6 *Olea europaea* cv. Leccino fruitlet *Olea europaea* cDNA | –2.68 | 0.00 | Unknown |
| FL683674 | FL683674 A_G15_D08_0414C_p1 *Olea europaea* cv. Leccino fruitlet *Olea europaea* cDNA | –2.68 | 0.01 | Oxidation-reduction process, response to oxidative stress |
| FL684179 | FL684179 A_A08_A04_0414F_p2 *Olea europaea* cv. Leccino fruitlet *Olea europaea* cDNA | –2.67 | 0.02 | Oxidation-reduction process, response to oxidative stress |
| GO243464 | GO243464 OEAA-070810_Plate2a11.b1 cDNA library from Olive leaves and fruits *Olea europaea* cDNA | –2.65 | 0.00 | Response to stress |
| FL683743 | FL683743 A_I14_E07_0414F_p2 *Olea europaea* cv. Leccino fruitlet *Olea europaea* cDNA | –2.65 | 0.01 | Oxidation-reduction process, response to oxidative stress |
| GO244563 | GO244563 OEAA-070810_Plate4p12.b1 cDNA library from Olive leaves and fruits *Olea europaea* cDNA | –2.64 | 0.01 | Unknown |
| GO244392 | GO244392 OEAA-070810_Plate4i03.b1 cDNA library from Olive leaves and fruits *Olea europaea* cDNA | –2.64 | 0.00 | Unknown |
| FL683672 | FL683672 A_P13_H07_0414F_p3 *Olea europaea* cv. Leccino fruitlet *Olea europaea* cDNA | –2.64 | 0.00 | Oxidation-reduction process, response to oxidative stress |
| GO246304 | GO246304 OEAA-070810_Plate9k06.b1 cDNA library from Olive leaves and fruits *Olea europaea* cDNA | –2.64 | 0.00 | Unknown |
| FL683591 | FL683591 D_A06_A03_0414F_p14 *Olea europaea* cv. Leccino fruitlet *Olea europaea* cDNA | –2.63 | 0.02 | Unknown |
| FL683442 | FL683442 B_G10_D05_0414F_p6 *Olea europaea* cv. Leccino fruitlet *Olea europaea* cDNA | –2.63 | 0.00 | Unknown |
| GO245097 | GO245097 OEAA-070810_Plate6g16.b1 cDNA library from Olive leaves and fruits *Olea europaea* cDNA | –2.62 | 0.00 | Oxidation-reduction process |
| GO245766 | GO245766 OEAA-070810_Plate8d09.b1 cDNA library from Olive leaves and fruits *Olea europaea* cDNA | –2.62 | 0.00 | Transmembrane transport, response to abiotic stimulus, monovalent inorganic cation transport, response to chemical stimulus, calcium ion transport, cellular divalent inorganic cation homeostasis, response to stress |
| FL683795 | FL683795 B_C09_B05_0414F_p5 *Olea europaea* cv. Leccino fruitlet *Olea europaea* cDNA | –2.61 | 0.01 | Terpene biosynthetic process, oxidation-reduction process |
| GO243434 | GO243434 OEAA-070810_Plate1p04.b1 cDNA library from Olive leaves and fruits *Olea europaea* cDNA | –2.61 | 0.01 | Unknown |
| FL684178 | FL684178 A_F23_C12_0414F_p3 *Olea europaea* cv. Leccino fruitlet *Olea europaea* cDNA | –2.61 | 0.01 | Oxidation-reduction process |
| FL684182 | FL684182 D_M10_G05_0414F_p14 *Olea europaea* cv. Leccino fruitlet *Olea europaea* cDNA | –2.61 | 0.02 | Flavonoid biosynthetic process, oxidation-reduction process |
| FL683981 | FL683981 A_H19_D10_0414F_p3 *Olea europaea* cv. Leccino fruitlet *Olea europaea* cDNA | –2.61 | 0.01 | Monoterpenoid biosynthetic process, oxidation-reduction process |
| GO243445 | GO243445 OEAA-070810_Plate1p15.b1 cDNA library from Olive leaves and fruits *Olea europaea* cDNA | –2.61 | 0.01 | Cutin biosynthetic process, phosphatidylglycerol biosynthetic process, regulation of meristem growth, |
| GO246071 | GO246071 OEAA-070810_Plate9a09.b1 cDNA library from Olive leaves and fruits *Olea europaea* cDNA | –2.61 | 0.02 | Transmembrane transport, response to abiotic stimulus, monovalent inorganic cation transport, response to chemical stimulus, calcium ion transport, cellular divalent inorganic cation homeostasis, response to stress |
| GO242810 | GO242810 OEAA-070810_Plate10e12.b1 cDNA library from Olive leaves and fruits *Olea europaea* cDNA | –2.61 | 0.00 | Unknown |
| GO243666 | GO243666 OEAA-070810_Plate2j05.b1 cDNA library from Olive leaves and fruits *Olea europaea* cDNA | –2.60 | 0.00 | Tetracyclic triterpenoid biosynthetic process, pentacyclic triterpenoid biosynthetic process, tricyclic triterpenoid biosynthetic process, thalianol metabolic process, root development |
| gw1.1883.5.1 | protein kinase family protein | –2.60 | 0.00 | Protein phosphorylation, transferase activity |
| FL684124 | FL684124 B_O16_H08_0414F_p6 *Olea europaea* cv. Leccino fruitlet *Olea europaea* cDNA | –2.60 | 0.01 | Oxidation-reduction process |
| FL683559 | FL683559 A_M08_G04_0414F_p2 *Olea europaea* cv. Leccino fruitlet *Olea europaea* cDNA | –2.60 | 0.00 | Response to nematode, oxidation-reduction process |
| GO244822 | GO244822 OEAA-070810_Plate5k22.b1 cDNA library from Olive leaves and fruits *Olea europaea* cDNA | –2.59 | 0.00 | Unknown |
| FL683728 | FL683728 D_F04_C02_0414F_p12 *Olea europaea* cv. Leccino fruitlet *Olea europaea* cDNA | –2.59 | 0.01 | Oxidation-reduction process |
| GO245488 | GO245488 OEAA-070810_Plate7h13.b1 cDNA library from Olive leaves and fruits *Olea europaea* cDNA | –2.59 | 0.00 | Defense response to fungus, incompatible interaction, salicylic acid metabolic process, systemic acquired resistance, salicylic acid mediated signaling pathway, |
| FL683741 | FL683741 A_K13_F07_0414C_p1 *Olea europaea* cv. Leccino fruitlet *Olea europaea* cDNA | –2.59 | 0.00 | Unknown |
| GO243140 | GO243140 OEAA-070810_Plate1c11.b1 cDNA library from Olive leaves and fruits *Olea europaea* cDNA | –2.58 | 0.00 | Unknown |
| GO244016 | GO244016 OEAA-070810_Plate3i08.b1 cDNA library from Olive leaves and fruits *Olea europaea* cDNA | –2.58 | 0.01 | Defense response to virus |
| GO244609 | GO244609 OEAA-070810_Plate5b13.b1 cDNA library from Olive leaves and fruits *Olea europaea* cDNA | –2.57 | 0.00 | Cutin biosynthetic process, phosphatidylglycerol biosynthetic process, regulation of meristem growth, |
| GO244064 | GO244064 OEAA-070810_Plate3k08.b1 cDNA library from Olive leaves and fruits *Olea europaea* cDNA | –2.56 | 0.00 | Unknown |
| GO245605 | GO245605 OEAA-070810_Plate7m13.b1 cDNA library from Olive leaves and fruits *Olea europaea* cDNA | –2.56 | 0.00 | Metabolic process |
| GO246374 | GO246374 OEAA-070810_Plate9n09.b1 cDNA library from Olive leaves and fruits *Olea europaea* cDNA | –2.55 | 0.02 | Transmembrane transport, response to abiotic stimulus, monovalent inorganic cation transport, response to chemical stimulus, calcium ion transport, cellular divalent inorganic cation homeostasis, response to stress |
| GO244205 | GO244205 OEAA-070810_Plate4a06.b1 cDNA library from Olive leaves and fruits *Olea europaea* cDNA | –2.55 | 0.00 | Unknown |
| GO243546 | GO243546 OEAA-070810_Plate2d24.b1 cDNA library from Olive leaves and fruits *Olea europaea* cDNA | –2.55 | 0.00 | Unknown |
| GO245383 | GO245383 OEAA-070810_Plate7c24.b1 cDNA library from Olive leaves and fruits *Olea europaea* cDNA | –2.55 | 0.00 | Unknown |
| grail3.0111003801 | calmodulin-binding family protein | –2.54 | 0.02 | Chromatin silencing by small RNA, RNA interference, microtubule cytoskeleton organization, DNA methylation, methylation-dependent chromatin silencing, regulation of DNA replication, chromatin silencing, histone H3-K9 methylation, cell proliferation, cytokinesis by cell plate formation, gene silencing by RNA, petal formation, regulation of cell cycle, DNA replication initiation, sepal formation |
| GO245902 | GO245902 OEAA-070810_Plate8j03.b1 cDNA library from Olive leaves and fruits *Olea europaea* cDNA | –2.53 | 0.00 | Transmembrane transport, response to abiotic stimulus, monovalent inorganic cation transport, response to chemical stimulus, calcium ion transport, cellular divalent inorganic cation homeostasis, response to stress |
| GO243051 | GO243051 OEAA-070810_Plate10o15.b1 cDNA library from Olive leaves and fruits *Olea europaea* cDNA | –2.53 | 0.00 | Calcium ion transport, cellular divalent inorganic cation homeostasis, monovalent inorganic cation transport, response to abiotic stimulus, response to chemical stimulus, response to stres, transmembrane transport, |
| GO244118 | GO244118 OEAA-070810_Plate3m14.b1 cDNA library from Olive leaves and fruits *Olea europaea* cDNA | –2.53 | 0.01 | Unknown |
| GO244186 | GO244186 OEAA-070810_Plate3p11.b1 cDNA library from Olive leaves and fruits *Olea europaea* cDNA | –2.53 | 0.00 | Response to cycloheximide, cellular response to phosphate starvation, protein phosphorylation, response to light stimulus |
| GO243040 | GO243040 OEAA-070810_Plate10o04.b1 cDNA library from Olive leaves and fruits *Olea europaea* cDNA | –2.53 | 0.00 | Transmembrane transport, response to abiotic stimulus, monovalent inorganic cation transport, response to chemical stimulus, calcium ion transport, cellular divalent inorganic cation homeostasis, response to stress |
| GO245316 | GO245316 OEAA-070810_Plate7a04.b1 cDNA library from Olive leaves and fruits *Olea europaea* cDNA | –2.52 | 0.00 | Transmembrane transport, response to abiotic stimulus, monovalent inorganic cation transport, response to chemical stimulus, calcium ion transport, cellular divalent inorganic cation homeostasis, response to stress |
| GO246408 | GO246408 OEAA-070810_Plate9o19.b1 cDNA library from Olive leaves and fruits *Olea europaea* cDNA | –2.52 | 0.02 | Transmembrane transport, response to abiotic stimulus, monovalent inorganic cation transport, response to chemical stimulus, calcium ion transport, cellular divalent inorganic cation homeostasis, response to stress |
| GO244589 | GO244589 OEAA-070810_Plate5a16.b1 cDNA library from Olive leaves and fruits *Olea europaea* cDNA | –2.52 | 0.00 | Regulation of plant-type hypersensitive response, positive regulation of flavonoid biosynthetic process, lipid transport, protein targeting to membrane |
| GO244336 | GO244336 OEAA-070810_Plate4f18.b1 cDNA library from Olive leaves and fruits *Olea europaea* cDNA | –2.52 | 0.00 | Unknown |
| GO244011 | GO244011 OEAA-070810_Plate3i03.b1 cDNA library from Olive leaves and fruits *Olea europaea* cDNA | –2.52 | 0.00 | Protein phosphorylation, signal transduction |
| FN998000 | FN998000 FN998000 *Olea europaea* flower *Olea europaea* cDNA clone c1-5-B1 | –2.50 | 0.00 | Transmembrane transport, hydrogen peroxide catabolic process, Golgi organization, vacuole organization |
| GO243868 | GO243868 OEAA-070810_Plate3c03.b1 cDNA library from Olive leaves and fruits *Olea europaea* cDNA | –2.50 | 0.00 | Unknown |
| GO245983 | GO245983 OEAA-070810_Plate8m14.b1 cDNA library from Olive leaves and fruits *Olea europaea* cDNA | –2.50 | 0.00 | Proteolysis |
| GO245255 | GO245255 OEAA-070810_Plate6n12.b1 cDNA library from Olive leaves and fruits *Olea europaea* cDNA | –2.50 | 0.00 | Regulation of glucosinolate biosynthetic process |
| GO243690 | GO243690 OEAA-070810_Plate2k05.b1 cDNA library from Olive leaves and fruits *Olea europaea* cDNA | –2.50 | 0.00 | Response to L-ascorbic acid, plant-type cell wall cellulose metabolic process, cell wall pectin metabolic process, proteolysis, response to light stimulus |
| GO244737 | GO244737 OEAA-070810_Plate5h06.b1 cDNA library from Olive leaves and fruits *Olea europaea* cDNA | –2.50 | 0.00 | Unknown |
| GO244038 | GO244038 OEAA-070810_Plate3j06.b1 cDNA library from Olive leaves and fruits *Olea europaea* cDNA | –2.50 | 0.00 | Unknown |
| GO244103 | GO244103 OEAA-070810_Plate3l23.b1 cDNA library from Olive leaves and fruits *Olea europaea* cDNA | –2.49 | 0.01 | Unknown |
| GO243038 | GO243038 OEAA-070810_Plate10o02.b1 cDNA library from Olive leaves and fruits *Olea europaea* cDNA | –2.49 | 0.00 | Calcium ion transport, cellular divalent inorganic cation homeostasis, monovalent inorganic cation transport, response to abiotic stimulus, response to chemical stimulus, response to stres, transmembrane transport, |
| GO243771 | GO243771 OEAA-070810_Plate2n24.b1 cDNA library from Olive leaves and fruits *Olea europaea* cDNA | –2.48 | 0.00 | Lipid metabolic process |
| GO243421 | GO243421 OEAA-070810_Plate1o15.b1 cDNA library from Olive leaves and fruits *Olea europaea* cDNA | –2.47 | 0.01 | Defense response, microtubule-based movement, response to biotic stimulus, |
| gw1.VIII.780.1 | EXS family protein / ERD1/XPR1/SYG1 family protein | –2.47 | 0.00 | Phosphate ion transport, negative regulation of transcription-DNA dependent, cellular response to phosphate starvation, galactolipid biosynthetic process |
| GO245198 | GO245198 OEAA-070810_Plate6l01.b1 cDNA library from Olive leaves and fruits *Olea europaea* cDNA | –2.47 | 0.00 | Regulation of cellular process |
| GO243324 | GO243324 OEAA-070810_Plate1k13.b1 cDNA library from Olive leaves and fruits *Olea europaea* cDNA | –2.46 | 0.00 | Cellular response to nitrogen starvation |
| GO244976 | GO244976 OEAA-070810_Plate6b14.b1 cDNA library from Olive leaves and fruits *Olea europaea* cDNA | –2.46 | 0.01 | Alcohol metabolic process, stamen development, petal development, ovule development, oxidation-reduction process |
| estExt_fgenesh4_pg.C_LG_VIII0467 | ATEXP4, ATHEXP ALPHA 1.6, ATEXPA4 \| ATEXPA4 (ARABIDOPSIS THALIANA EXPANSIN A4) | –2.46 | 0.03 | Syncytium formation, cell-wall organizationcell growth, cell wall loosening |
| GO244213 | GO244213 OEAA-070810_Plate4a14.b1 cDNA library from Olive leaves and fruits *Olea europaea* cDNA | –2.46 | 0.00 | Unknown |
| GO246134 | GO246134 OEAA-070810_Plate9c24.b1 cDNA library from Olive leaves and fruits *Olea europaea* cDNA | –2.45 | 0.00 | Transmembrane transport, response to abiotic stimulus, monovalent inorganic cation transport, response to chemical stimulus, calcium ion transport, cellular divalent inorganic cation homeostasis, response to stress |
| GO244267 | GO244267 OEAA-070810_Plate4c21.b1 cDNA library from Olive leaves and fruits *Olea europaea* cDNA | –2.45 | 0.00 | Unknown |
| GO245724 | GO245724 OEAA-070810_Plate8b15.b1 cDNA library from Olive leaves and fruits *Olea europaea* cDNA | –2.45 | 0.00 | Transmembrane transport, response to abiotic stimulus, monovalent inorganic cation transport, response to chemical stimulus, calcium ion transport, cellular divalent inorganic cation homeostasis, response to stress |
| GO244040 | GO244040 OEAA-070810_Plate3j08.b1 cDNA library from Olive leaves and fruits *Olea europaea* cDNA | –2.44 | 0.00 | Unknown |
| GO243683 | GO243683 OEAA-070810_Plate2j22.b1 cDNA library from Olive leaves and fruits *Olea europaea* cDNA | –2.43 | 0.01 | Unknown |
| GO243962 | GO243962 OEAA-070810_Plate3g01.b1 cDNA library from Olive leaves and fruits *Olea europaea* cDNA | –2.43 | 0.01 | Alcohol metabolic process, response to cold, ER to Golgi vesicle-mediated transport, oxidation-reduction process |
| GO243016 | GO243016 OEAA-070810_Plate10n04.b1 cDNA library from Olive leaves and fruits *Olea europaea* cDNA | –2.43 | 0.00 | Transmembrane transport, response to abiotic stimulus, monovalent inorganic cation transport, response to chemical stimulus, calcium ion transport, cellular divalent inorganic cation homeostasis, response to stress |
| GO244325 | GO244325 OEAA-070810_Plate4f07.b1 cDNA library from Olive leaves and fruits *Olea europaea* cDNA | –2.43 | 0.00 | Unknown |
| GO244889 | GO244889 OEAA-070810_Plate5n19.b1 cDNA library from Olive leaves and fruits *Olea europaea* cDNA | –2.43 | 0.00 | Proteolysis |
| GO243685 | GO243685 OEAA-070810_Plate2j24.b1 cDNA library from Olive leaves and fruits *Olea europaea* cDNA | –2.42 | 0.01 | Brassinosteroid biosynthetic process, cellular response to iron ion starvation, hyperosmotic salinity response, iron ion transport, nitrate transport, oxidation-reduction process, response to cold, response to desiccation, response to nitrate, response to oxidative stres, trichoblast differentiation |
| GO244702 | GO244702 OEAA-070810_Plate5f15.b1 cDNA library from Olive leaves and fruits *Olea europaea* cDNA | –2.42 | 0.00 | Calcium ion transport, cellular cation homeostasis, Golgi organization, methylammonium transmembrane transport, response to salt stres, urea transmembrane transport, water transport, |
| gw1.309.24.1 | protein kinase family protein | –2.42 | 0.03 | Protein phosphorylation, transferase activity |
| FL684270 | FL684270 A_I17_E09_0414C_p1 *Olea europaea* cv. Leccino fruitlet *Olea europaea* cDNA | –2.42 | 0.00 | Terpenoid biosynthetic process |
| GO243065 | GO243065 OEAA-070810_Plate10p05.b1 cDNA library from Olive leaves and fruits *Olea europaea* cDNA | –2.42 | 0.00 | Transmembrane transport, response to abiotic stimulus, monovalent inorganic cation transport, response to chemical stimulus, calcium ion transport, cellular divalent inorganic cation homeostasis, response to stress |
| FL683889 | FL683889 A_C17_B09_0414C_p1 *Olea europaea* cv. Leccino fruitlet *Olea europaea* cDNA | –2.42 | 0.00 | Unknown |
| GO245485 | GO245485 OEAA-070810_Plate7h10.b1 cDNA library from Olive leaves and fruits *Olea europaea* cDNA | –2.41 | 0.00 | Unknown |
| GO245145 | GO245145 OEAA-070810_Plate6i18.b1 cDNA library from Olive leaves and fruits *Olea europaea* cDNA | –2.40 | 0.00 | Cell wall biogenesis, trichome morphogenesis |
| gw1.XIII.2085.1 | protein kinase family protein | –2.40 | 0.01 | Protein phosphorylation, transferase activity |
| fgenesh4_pg.C_LG_X001844 | ATEXP4, ATHEXP ALPHA 1.6, ATEXPA4 \| ATEXPA4 (ARABIDOPSIS THALIANA EXPANSIN A4) | –2.40 | 0.00 | Syncytium formation, unidimensional cell growth, plant-type cell wall loosening, plant-type cell wall loosening, syncytium formation, unidimensional cell growth, plant-type cell wall modification involved in multidimensional cell growth, plant-type cell wall organization |
| gw1.XIX.1830.1 | hydrolase, alpha/beta fold family protein | –2.40 | 0.02 | Unknown |
| GO245584 | GO245584 OEAA-070810_Plate7l16.b1 cDNA library from Olive leaves and fruits *Olea europaea* cDNA | –2.40 | 0.00 | Unknown |
| GO244349 | GO244349 OEAA-070810_Plate4g07.b1 cDNA library from Olive leaves and fruits *Olea europaea* cDNA | –2.40 | 0.01 | Unknown |
| GO243919 | GO243919 OEAA-070810_Plate3e06.b1 cDNA library from Olive leaves and fruits *Olea europaea* cDNA | –2.39 | 0.00 | Unknown |
| GO246108 | GO246108 OEAA-070810_Plate9b22.b1 cDNA library from Olive leaves and fruits *Olea europaea* cDNA | –2.39 | 0.00 | Calcium ion transport, cellular divalent inorganic cation homeostasis, monovalent inorganic cation transport, response to abiotic stimulus, response to chemical stimulus, response to stres, transmembrane transport, |
| GO246350 | GO246350 OEAA-070810_Plate9m08.b1 cDNA library from Olive leaves and fruits *Olea europaea* cDNA | –2.39 | 0.00 | Cell adhesion |
| GO243632 | GO243632 OEAA-070810_Plate2h18.b1 cDNA library from Olive leaves and fruits *Olea europaea* cDNA | –2.39 | 0.00 | Cutin biosynthetic process, double fertilization forming a zygote and endosperm, fatty acid oxidation, flower development, pollen tube development, vernalization response, |
| GO244330 | GO244330 OEAA-070810_Plate4f12.b1 cDNA library from Olive leaves and fruits *Olea europaea* cDNA | –2.39 | 0.00 | Unknown |
| GO243069 | GO243069 OEAA-070810_Plate10p09.b1 cDNA library from Olive leaves and fruits *Olea europaea* cDNA | –2.37 | 0.00 | Transmembrane transport, response to abiotic stimulus, monovalent inorganic cation transport, response to chemical stimulus, calcium ion transport, cellular divalent inorganic cation homeostasis, response to stress |
| FL683608 | FL683608 A_H07_D04_0414F_p3 *Olea europaea* cv. Leccino fruitlet *Olea europaea* cDNA | –2.36 | 0.02 | Oxidation-reduction process, response to oxidative stress |
| GO243149 | GO243149 OEAA-070810_Plate1c20.b1 cDNA library from Olive leaves and fruits *Olea europaea* cDNA | –2.36 | 0.03 | Unknown |
| GO245040 | GO245040 OEAA-070810_Plate6e07.b1 cDNA library from Olive leaves and fruits *Olea europaea* cDNA | –2.36 | 0.00 | Multidimensional cell growth, root hair elongation, polysaccharide biosynthetic process, cell tip growth, cell wall organization, auxin polar transport, regulation of cell size, anthocyanin accumulation in tissues in response to UV light, cysteine biosynthetic process, pattern specification process |
| GO244184 | GO244184 OEAA-070810_Plate3p09.b1 cDNA library from Olive leaves and fruits *Olea europaea* cDNA | –2.36 | 0.01 | Unknown |
| GO243574 | GO243574 OEAA-070810_Plate2f06.b1 cDNA library from Olive leaves and fruits *Olea europaea* cDNA | –2.36 | 0.01 | Unknown |
| GO246111 | GO246111 OEAA-070810_Plate9c01.b1 cDNA library from Olive leaves and fruits *Olea europaea* cDNA | –2.35 | 0.00 | Transmembrane transport, response to abiotic stimulus, monovalent inorganic cation transport, response to chemical stimulus, calcium ion transport, cellular divalent inorganic cation homeostasis, response to stress |
| GO243500 | GO243500 OEAA-070810_Plate2b24.b1 cDNA library from Olive leaves and fruits *Olea europaea* cDNA | –2.35 | 0.00 | Unknown |
| GO245561 | GO245561 OEAA-070810_Plate7k15.b1 cDNA library from Olive leaves and fruits *Olea europaea* cDNA | –2.35 | 0.00 | Unknown |
| GO245399 | GO245399 OEAA-070810_Plate7d16.b1 cDNA library from Olive leaves and fruits *Olea europaea* cDNA | –2.35 | 0.01 | Xylan biosynthetic process, glucuronoxylan metabolic process |
| GO243082 | GO243082 OEAA-070810_Plate10p22.b1 cDNA library from Olive leaves and fruits *Olea europaea* cDNA | –2.35 | 0.00 | Transmembrane transport, response to abiotic stimulus, monovalent inorganic cation transport, response to chemical stimulus, calcium ion transport, cellular divalent inorganic cation homeostasis, response to stress |
| GO244446 | GO244446 OEAA-070810_Plate4k11.b1 cDNA library from Olive leaves and fruits *Olea europaea* cDNA | –2.34 | 0.00 | Unknown |
| GO246429 | GO246429 OEAA-070810_Plate9p17.b1 cDNA library from Olive leaves and fruits *Olea europaea* cDNA | –2.33 | 0.02 | Unknown |
| GO243638 | GO243638 OEAA-070810_Plate2h24.b1 cDNA library from Olive leaves and fruits *Olea europaea* cDNA | –2.33 | 0.00 | Unknown |
| GO242745 | GO242745 OEAA-070810_Plate10b19.b1 cDNA library from Olive leaves and fruits *Olea europaea* cDNA | –2.32 | 0.00 | Calcium ion transport, cellular divalent inorganic cation homeostasis, monovalent inorganic cation transport, response to abiotic stimulus, response to chemical stimulus, response to stres, transmembrane transport, |
| FL683558 | FL683558 A_M17_G09_0414F_p1 *Olea europaea* cv. Leccino fruitlet *Olea europaea* cDNA | –2.32 | 0.00 | Carbon dioxide transport, glycolysis, Golgi organization, hyperosmotic response, regulation of protein localization, response to cadmium ion, response to salt stres, response to temperature stimulus, response to water deprivation, transmembrane transport, water transport, |
| GO242827 | GO242827 OEAA-070810_Plate10f05.b1 cDNA library from Olive leaves and fruits *Olea europaea* cDNA | –2.32 | 0.00 | Transmembrane transport, response to abiotic stimulus, monovalent inorganic cation transport, response to chemical stimulus, calcium ion transport, cellular divalent inorganic cation homeostasis, response to stress |
| GO245355 | GO245355 OEAA-070810_Plate7b20.b1 cDNA library from Olive leaves and fruits *Olea europaea* cDNA | –2.31 | 0.00 | Transmembrane transport, response to abiotic stimulus, monovalent inorganic cation transport, response to chemical stimulus, calcium ion transport, cellular divalent inorganic cation homeostasis, response to stress |
| gw1.378.10.1 | EXS family protein / ERD1/XPR1/SYG1 family protein | –2.31 | 0.00 | Phosphate ion transport, negative regulation of transcription-DNA dependent, cellular response to phosphate starvation, galactolipid biosynthetic process |
| gw1.41.74.1 | protein kinase family protein | –2.31 | 0.00 | Protein phosphorylation, transferase activity |
| GO245493 | GO245493 OEAA-070810_Plate7h18.b1 cDNA library from Olive leaves and fruits *Olea europaea* cDNA | –2.31 | 0.00 | Unknown |
| GO244544 | GO244544 OEAA-070810_Plate4o17.b1 cDNA library from Olive leaves and fruits *Olea europaea* cDNA | –2.29 | 0.00 | Tetracyclic triterpenoid biosynthetic process, pentacyclic triterpenoid biosynthetic process, tricyclic triterpenoid biosynthetic process, thalianol metabolic process, root development |
| FL683832 | FL683832 A_O03_H02_0414C_p1 *Olea europaea* cv. Leccino fruitlet *Olea europaea* cDNA | –2.29 | 0.01 | Stomatal complex morphogenesis, auxin mediated signaling pathway, regulation of meristem growth, transmembrane receptor protein tyrosine kinase signaling pathway, protein phosphorylation, brassinosteroid mediated signaling pathway, leaf vascular tissue pattern formation, phloem transport, response to molecule of bacterial origin, stamen development |
| GO246343 | GO246343 OEAA-070810_Plate9l24.b1 cDNA library from Olive leaves and fruits *Olea europaea* cDNA | –2.28 | 0.01 | Transmembrane transport, response to abiotic stimulus, monovalent inorganic cation transport, response to chemical stimulus, calcium ion transport, cellular divalent inorganic cation homeostasis, response to stress |
| GO244946 | GO244946 OEAA-070810_Plate6a07.b1 cDNA library from Olive leaves and fruits *Olea europaea* cDNA | –2.28 | 0.00 | Divalent metal ion transport, cellular cation homeostasis |
| GO243516 | GO243516 OEAA-070810_Plate2c18.b1 cDNA library from Olive leaves and fruits *Olea europaea* cDNA | –2.28 | 0.00 | Unknown |
| eugene3.00180818 | IAA33 \| IAA33 (indoleacetic acid-induced protein 33); transcription factor | –2.28 | 0.03 | Response to auxin stimulus, regulation of transcription |
| GO242706 | GO242706 OEAA-070810_Plate10a04.b1 cDNA library from Olive leaves and fruits *Olea europaea* cDNA | –2.28 | 0.00 | Unknown |
| GO245932 | GO245932 OEAA-070810_Plate8k09.b1 cDNA library from Olive leaves and fruits *Olea europaea* cDNA | –2.27 | 0.00 | Unknown |
| GO246284 | GO246284 OEAA-070810_Plate9j07.b1 cDNA library from Olive leaves and fruits *Olea europaea* cDNA | –2.27 | 0.01 | Unknown |
| GO246152 | GO246152 OEAA-070810_Plate9d18.b1 cDNA library from Olive leaves and fruits *Olea europaea* cDNA | –2.27 | 0.00 | Transmembrane transport, response to abiotic stimulus, monovalent inorganic cation transport, response to chemical stimulus, calcium ion transport, cellular divalent inorganic cation homeostasis, response to stress |
| FN998481 | FN998481 FN998481 *Olea europaea* flower *Olea europaea* cDNA clone c2-3-B1 | –2.27 | 0.02 | Metabolic process |
| GO245281 | GO245281 OEAA-070810_Plate6o15.b1 cDNA library from Olive leaves and fruits *Olea europaea* cDNA | –2.27 | 0.00 | Lipid metabolic process |
| fgenesh4_pm.C_LG_I000876 | protein kinase, putative | –2.27 | 0.02 | Microtubule nucleation, protein phosphorylation, transferase activity |
| GO246141 | GO246141 OEAA-070810_Plate9d07.b1 cDNA library from Olive leaves and fruits *Olea europaea* cDNA | –2.27 | 0.02 | Metabolic process |
| estExt_Genewise1_v1.C_LG_IX1501 | DNA binding | –2.26 | 0.00 | Endoplasmic reticulum unfolded protein response, response to xenobiotic stimulus, regulation of transcription |
| grail3.0001007601 | similar to unknown protein [Arabidopsis thaliana] (TAIR:AT5G22310.1) | –2.26 | 0.02 | Unknown |
| GO243108 | GO243108 OEAA-070810_Plate1b02.b1 cDNA library from Olive leaves and fruits *Olea europaea* cDNA | –2.26 | 0.00 | Proteolysis |
| fgenesh4_pg.C_scaffold40000084 | FAD-binding domain-containing protein | –2.25 | 0.04 | Oxidation-reduction process |
| gw1.135.61.1 | protein kinase family protein | –2.25 | 0.01 | Protein phosphorylation, transferase activity |
| GO246184 | GO246184 OEAA-070810_Plate9f02.b1 cDNA library from Olive leaves and fruits *Olea europaea* cDNA | –2.24 | 0.01 | Calcium ion transport, cellular divalent inorganic cation homeostasis, monovalent inorganic cation transport, response to abiotic stimulus, response to chemical stimulus, response to stres, transmembrane transport, |
| FL684309 | FL684309 B_O15_H08_0414F_p5 *Olea europaea* cv. Leccino fruitlet *Olea europaea* cDNA | –2.24 | 0.01 | Unknown |
| GO243378 | GO243378 OEAA-070810_Plate1m20.b1 cDNA library from Olive leaves and fruits *Olea europaea* cDNA | –2.24 | 0.00 | Carbohydrate metabolic process, para-aminobenzoic acid metabolic process, response to salicylic acid stimulus |
| GO245656 | GO245656 OEAA-070810_Plate7o16.b1 cDNA library from Olive leaves and fruits *Olea europaea* cDNA | –2.24 | 0.01 | Transmembrane transport, response to abiotic stimulus, monovalent inorganic cation transport, response to chemical stimulus, calcium ion transport, cellular divalent inorganic cation homeostasis, response to stress |
| GO243192 | GO243192 OEAA-070810_Plate1e20.b1 cDNA library from Olive leaves and fruits *Olea europaea* cDNA | –2.23 | 0.00 | Proteolysis |
| FL684191 | FL684191 D_K13_F07_0414F_p13 *Olea europaea* cv. Leccino fruitlet *Olea europaea* cDNA | –2.22 | 0.00 | Cysteine biosynthetic process |
| GO245788 | GO245788 OEAA-070810_Plate8e07.b1 cDNA library from Olive leaves and fruits *Olea europaea* cDNA | –2.22 | 0.00 | Oxidation-reduction process |
| gw1.XVI.2989.1 | protein kinase family protein | –2.22 | 0.02 | Protein phosphorylation, transferase activity |
| GO244100 | GO244100 OEAA-070810_Plate3l20.b1 cDNA library from Olive leaves and fruits *Olea europaea* cDNA | –2.21 | 0.00 | Unknown |
| GO243852 | GO243852 OEAA-070810_Plate3b11.b1 cDNA library from Olive leaves and fruits *Olea europaea* cDNA | –2.21 | 0.00 | Unknown |
| GO243268 | GO243268 OEAA-070810_Plate1i01.b1 cDNA library from Olive leaves and fruits *Olea europaea* cDNA | –2.21 | 0.02 | Unknown |
| GO243390 | GO243390 OEAA-070810_Plate1n08.b1 cDNA library from Olive leaves and fruits *Olea europaea* cDNA | –2.21 | 0.01 | Methylation |
| GO242755 | GO242755 OEAA-070810_Plate10c05.b1 cDNA library from Olive leaves and fruits *Olea europaea* cDNA | –2.21 | 0.02 | Transport |
| GO245103 | GO245103 OEAA-070810_Plate6g23.b1 cDNA library from Olive leaves and fruits *Olea europaea* cDNA | –2.21 | 0.01 | Unknown |
| FL684271 | FL684271 C_B18_A09_0414F_p8 *Olea europaea* cv. Leccino fruitlet *Olea europaea* cDNA | –2.20 | 0.01 | Oxidation-reduction process |
| gw1.XV.570.1 | protein kinase family protein | –2.20 | 0.01 | Protein phosphorylation, transferase activity |
| GO243061 | GO243061 OEAA-070810_Plate10p01.b1 cDNA library from Olive leaves and fruits *Olea europaea* cDNA | –2.20 | 0.01 | Transmembrane transport, response to abiotic stimulus, monovalent inorganic cation transport, response to chemical stimulus, calcium ion transport, cellular divalent inorganic cation homeostasis, response to stress |
| FL684093 | FL684093 C_P23_H12_0414F_p7 *Olea europaea* cv. Leccino fruitlet *Olea europaea* cDNA | –2.19 | 0.00 | Response to water deprivation, protein folding, response to salt stress, heat acclimation, flower development, protein stabilization, cellular response to calcium ion, leaf development, response to arsenic-containing substance, response to high light intensity, response to hydrogen peroxide, defense response to bacterium, incompatible interaction |
| GO246009 | GO246009 OEAA-070810_Plate8n16.b1 cDNA library from Olive leaves and fruits *Olea europaea* cDNA | –2.19 | 0.01 | Calcium ion transport, cellular divalent inorganic cation homeostasis, monovalent inorganic cation transport, response to chemical stimulus, response to stres, transmembrane transport, |
| GO244398 | GO244398 OEAA-070810_Plate4i09.b1 cDNA library from Olive leaves and fruits *Olea europaea* cDNA | –2.19 | 0.00 | Lipid metabolic process |
| GO245431 | GO245431 OEAA-070810_Plate7f01.b1 cDNA library from Olive leaves and fruits *Olea europaea* cDNA | –2.19 | 0.00 | Transmembrane transport, response to abiotic stimulus, monovalent inorganic cation transport, response to chemical stimulus, calcium ion transport, cellular divalent inorganic cation homeostasis, response to stress |
| GO244530 | GO244530 OEAA-070810_Plate4o03.b1 cDNA library from Olive leaves and fruits *Olea europaea* cDNA | –2.18 | 0.00 | Cellular response to phosphate starvation, response to misfolded protein, fatty acid beta-oxidation, galactolipid biosynthetic process, proteasomal ubiquitin-dependent protein catabolic process, cellular response to water deprivation, toxin catabolic process, proteasome core complex assembly, negative regulation of transcription, DNA-dependent, protein ubiquitination, postreplication repair |
| GO246175 | GO246175 OEAA-070810_Plate9e17.b1 cDNA library from Olive leaves and fruits *Olea europaea* cDNA | –2.18 | 0.01 | Calcium ion transport, cellular divalent inorganic cation homeostasis, monovalent inorganic cation transport, response to abiotic stimulus, response to chemical stimulus, response to stres, transmembrane transport, |
| GO243286 | GO243286 OEAA-070810_Plate1i20.b1 cDNA library from Olive leaves and fruits *Olea europaea* cDNA | –2.18 | 0.00 | Pentose-phosphate shunt, glucosinolate metabolic process, unsaturated fatty acid biosynthetic process, starch biosynthetic process, photosystem II assembly, isopentenyl diphosphate biosynthetic process, mevalonate-independent pathway, vitamin metabolic process, glycine catabolic process, lipoate metabolic process, chlorophyll biosynthetic process, coenzyme biosynthetic process, leaf morphogenesis, PSII associated light-harvesting complex II catabolic process, glycolysis, jasmonic acid biosynthetic process, cell differentiation, response to cadmium ion, cysteine biosynthetic process, response to abscisic acid stimulus, plastid organization, positive regulation of transcription, DNA-dependent, aromatic amino acid family biosynthetic process, rRNA processing |
| GO244243 | GO244243 OEAA-070810_Plate4b21.b1 cDNA library from Olive leaves and fruits *Olea europaea* cDNA | –2.18 | 0.00 | Unknown |
| FN998187 | FN998187 FN998187 *Olea europaea* flower *Olea europaea* cDNA clone c1-7-B10 | –2.17 | 0.01 | Unknown |
| GO245712 | GO245712 OEAA-070810_Plate8b01.b1 cDNA library from Olive leaves and fruits *Olea europaea* cDNA | –2.17 | 0.00 | Calcium ion transport, cellular divalent inorganic cation homeostasis, monovalent inorganic cation transport, response to abiotic stimulus, response to chemical stimulus, response to stres, transmembrane transport, |
| gw1.XIII.3068.1 | protein kinase family protein | –2.17 | 0.02 | Protein phosphorylation, transferase activity |
| estExt_fgenesh4_pg.C_LG_V1618 | similar to unknown protein [Arabidopsis thaliana] (TAIR:AT2G37210.1) | –2.17 | 0.00 | Unknown |
| GO245524 | GO245524 OEAA-070810_Plate7j02.b1 cDNA library from Olive leaves and fruits *Olea europaea* cDNA | –2.17 | 0.01 | Unknown |
| GO243874 | GO243874 OEAA-070810_Plate3c09.b1 cDNA library from Olive leaves and fruits *Olea europaea* cDNA | –2.16 | 0.01 | Response to chitin, oxidation-reduction process |
| GO244791 | GO244791 OEAA-070810_Plate5j14.b1 cDNA library from Olive leaves and fruits *Olea europaea* cDNA | –2.16 | 0.01 | Unknown |
| GO245338 | GO245338 OEAA-070810_Plate7b03.b1 cDNA library from Olive leaves and fruits *Olea europaea* cDNA | –2.16 | 0.00 | Unknown |
| GO246104 | GO246104 OEAA-070810_Plate9b18.b1 cDNA library from Olive leaves and fruits *Olea europaea* cDNA | –2.16 | 0.00 | Transmembrane transport, response to abiotic stimulus, monovalent inorganic cation transport, response to chemical stimulus, calcium ion transport, cellular divalent inorganic cation homeostasis, response to stress |
| GO243581 | GO243581 OEAA-070810_Plate2f13.b1 cDNA library from Olive leaves and fruits *Olea europaea* cDNA | –2.16 | 0.00 | Unknown |
| GO242887 | GO242887 OEAA-070810_Plate10h18.b1 cDNA library from Olive leaves and fruits *Olea europaea* cDNA | –2.15 | 0.00 | Transmembrane transport, response to abiotic stimulus, monovalent inorganic cation transport, response to chemical stimulus, calcium ion transport, cellular divalent inorganic cation homeostasis, response to stress |
| GO244054 | GO244054 OEAA-070810_Plate3j22.b1 cDNA library from Olive leaves and fruits *Olea europaea* cDNA | –2.15 | 0.00 | Unknown |
| GO244729 | GO244729 OEAA-070810_Plate5g22.b1 cDNA library from Olive leaves and fruits *Olea europaea* cDNA | –2.15 | 0.00 | Cell wall biogenesis, cysteine biosynthetic process, trichome morphogenesis, |
| GO244028 | GO244028 OEAA-070810_Plate3i20.b1 cDNA library from Olive leaves and fruits *Olea europaea* cDNA | –2.15 | 0.00 | Fatty acid biosynthetic process,oxidation-reduction process |
| GO245518 | GO245518 OEAA-070810_Plate7i20.b1 cDNA library from Olive leaves and fruits *Olea europaea* cDNA | –2.15 | 0.00 | Regulation of secondary cell wall biogenesis, regulation of transcription, DNA-dependent, regulation of transcription, DNA-dependent, response to abscisic acid stimulus, |
| GO243925 | GO243925 OEAA-070810_Plate3e12.b1 cDNA library from Olive leaves and fruits *Olea europaea* cDNA | –2.15 | 0.00 | Unknown |
| FL683960 | FL683960 D_J15_E08_0414F_p11 *Olea europaea* cv. Leccino fruitlet *Olea europaea* cDNA | –2.14 | 0.00 | Unknown |
| GO242772 | GO242772 OEAA-070810_Plate10c22.b1 cDNA library from Olive leaves and fruits *Olea europaea* cDNA | –2.14 | 0.00 | Calcium ion transport, cellular divalent inorganic cation homeostasis, monovalent inorganic cation transport, response to abiotic stimulus, response to chemical stimulus, response to stres, transmembrane transport, |
| GO245414 | GO245414 OEAA-070810_Plate7e07.b1 cDNA library from Olive leaves and fruits *Olea europaea* cDNA | –2.14 | 0.00 | Unknown |
| GO242805 | GO242805 OEAA-070810_Plate10e07.b1 cDNA library from Olive leaves and fruits *Olea europaea* cDNA | –2.13 | 0.00 | Transmembrane transport, response to abiotic stimulus, monovalent inorganic cation transport, response to chemical stimulus, calcium ion transport, cellular divalent inorganic cation homeostasis, response to stress |
| GO246053 | GO246053 OEAA-070810_Plate8p14.b1 cDNA library from Olive leaves and fruits *Olea europaea* cDNA | –2.13 | 0.01 | Starch biosynthetic process, maltose metabolic process |
| gw1.I.5279.1 | protein kinase family protein | –2.13 | 0.02 | Protein phosphorylation, transferase activity |
| estExt_fgenesh4_pg.C_LG_X0722 | beta-ketoacyl-CoA synthase, putative | –2.13 | 0.00 | Response to light stimulus, very long-chain fatty acid metabolic process, response to cold, cuticle development, fatty acid biosynthetic process, lipid biosynthetic process, metabolic process, transferase activity, oxidation-reduction process, oxidoreductase activity |
| FL684119 | FL684119 D_G07_D04_0414F_p13 *Olea europaea* cv. Leccino fruitlet *Olea europaea* cDNA | –2.13 | 0.00 | Pentose-phosphate shunt, response to salt stress, cellular carbohydrate metabolic process, glycolysis, gluconeogenesis, response to zinc ion, response to cadmium ion |
| GO245889 | GO245889 OEAA-070810_Plate8i14.b1 cDNA library from Olive leaves and fruits *Olea europaea* cDNA | –2.12 | 0.00 | Unknown |
| GO243745 | GO243745 OEAA-070810_Plate2m21.b1 cDNA library from Olive leaves and fruits *Olea europaea* cDNA | –2.12 | 0.00 | Unknown |
| GO243655 | GO243655 OEAA-070810_Plate2i17.b1 cDNA library from Olive leaves and fruits *Olea europaea* cDNA | –2.12 | 0.01 | Lipid metabolic process |
| GO242993 | GO242993 OEAA-070810_Plate10m05.b1 cDNA library from Olive leaves and fruits *Olea europaea* cDNA | –2.11 | 0.01 | Calcium ion transport, cellular divalent inorganic cation homeostasis, monovalent inorganic cation transport, response to abiotic stimulus, response to chemical stimulus, response to stres, transmembrane transport, |
| GO246093 | GO246093 OEAA-070810_Plate9b07.b1 cDNA library from Olive leaves and fruits *Olea europaea* cDNA | –2.11 | 0.00 | Calcium ion transport, cellular divalent inorganic cation homeostasis, monovalent inorganic cation transport, response to abiotic stimulus, response to chemical stimulus, response to stres, transmembrane transport, |
| GO244140 | GO244140 OEAA-070810_Plate3n12.b1 cDNA library from Olive leaves and fruits *Olea europaea* cDNA | –2.11 | 0.01 | Fatty acid metabolic process, oxidation-reduction process, |
| gw1.XI.1767.1 | protein kinase family protein | –2.11 | 0.02 | Protein phosphorylation, transferase activity |
| gw1.XVI.3261.1 | protein kinase family protein | –2.11 | 0.00 | Protein phosphorylation, transferase activity |
| GO243304 | GO243304 OEAA-070810_Plate1j17.b1 cDNA library from Olive leaves and fruits *Olea europaea* cDNA | –2.11 | 0.00 | Unknown |
| GO245865 | GO245865 OEAA-070810_Plate8h13.b1 cDNA library from Olive leaves and fruits *Olea europaea* cDNA | –2.11 | 0.01 | Cation transport, transmembrane transport |
| GO246019 | GO246019 OEAA-070810_Plate8o03.b1 cDNA library from Olive leaves and fruits *Olea europaea* cDNA | –2.10 | 0.02 | Unknown |
| GO244495 | GO244495 OEAA-070810_Plate4m14.b1 cDNA library from Olive leaves and fruits *Olea europaea* cDNA | –2.10 | 0.01 | Transcription, DNA-dependent |
| FN997936 | FN997936 FN997936 *Olea europaea* flower *Olea europaea* cDNA clone c1-4-D12 | –2.10 | 0.01 | Unknown |
| FL683577 | FL683577 D_J08_E04_0414F_p12 *Olea europaea* cv. Leccino fruitlet *Olea europaea* cDNA | –2.10 | 0.01 | Unknown |
| GO246394 | GO246394 OEAA-070810_Plate9o05.b1 cDNA library from Olive leaves and fruits *Olea europaea* cDNA | –2.10 | 0.01 | Calcium ion transport, cellular divalent inorganic cation homeostasis, monovalent inorganic cation transport, response to abiotic stimulus, response to chemical stimulus, response to stres, transmembrane transport, |
| FL684145 | FL684145 C_I19_E10_0414F_p9 *Olea europaea* cv. Leccino fruitlet *Olea europaea* cDNA | –2.10 | 0.01 | Glycolysis, lipid metabolic process, phosphorylation, response to cadmium ion, |
| GO245646 | GO245646 OEAA-070810_Plate7o06.b1 cDNA library from Olive leaves and fruits *Olea europaea* cDNA | –2.10 | 0.00 | Unknown |
| GO243543 | GO243543 OEAA-070810_Plate2d21.b1 cDNA library from Olive leaves and fruits *Olea europaea* cDNA | –2.10 | 0.00 | Unknown |
| GO244431 | GO244431 OEAA-070810_Plate4j20.b1 cDNA library from Olive leaves and fruits *Olea europaea* cDNA | –2.10 | 0.00 | Regulation of meristem growth, phosphorylation |
| GO245529 | GO245529 OEAA-070810_Plate7j07.b1 cDNA library from Olive leaves and fruits *Olea europaea* cDNA | –2.09 | 0.00 | Unknown |
| gw1.900.1.1 | ARAC7, ATROP9, RAC7, ROP9 \| ARAC7/ATROP9/RAC7/ROP9 (rho-related protein from plants 9); GTP binding | –2.09 | 0.00 | Actin filament organization, small GTPase mediated signal transduction, GTP catabolic process, signal transduction, protein transport |
| GO242964 | GO242964 OEAA-070810_Plate10k23.b1 cDNA library from Olive leaves and fruits *Olea europaea* cDNA | –2.09 | 0.05 | Calcium ion transport, cellular divalent inorganic cation homeostasis, monovalent inorganic cation transport, response to abiotic stimulus, response to chemical stimulus, response to stres, transmembrane transport, |
| grail3.0010037101 | ATOEP16-2, ATOEP16-S \| ATOEP16-2/ATOEP16-S; protein translocase | –2.09 | 0.00 | Protein transport |
| GO243205 | GO243205 OEAA-070810_Plate1f09.b1 cDNA library from Olive leaves and fruits *Olea europaea* cDNA | –2.08 | 0.00 | Methylation |
| GO243471 | GO243471 OEAA-070810_Plate2a18.b1 cDNA library from Olive leaves and fruits *Olea europaea* cDNA | –2.08 | 0.00 | Glucosinolate biosynthetic process, oxidation-reduction process |
| GO245725 | GO245725 OEAA-070810_Plate8b16.b1 cDNA library from Olive leaves and fruits *Olea europaea* cDNA | –2.08 | 0.00 | Calcium ion transport, cellular divalent inorganic cation homeostasis, monovalent inorganic cation transport, response to abiotic stimulus, response to chemical stimulus, response to stres, transmembrane transport, |
| GO245065 | GO245065 OEAA-070810_Plate6f08.b1 cDNA library from Olive leaves and fruits *Olea europaea* cDNA | –2.08 | 0.00 | Oxidation-reduction process |
| gw1.1027.1.1 | protein kinase family protein | –2.08 | 0.02 | Protein phosphorylation, transferase activity |
| GO245517 | GO245517 OEAA-070810_Plate7i19.b1 cDNA library from Olive leaves and fruits *Olea europaea* cDNA | –2.08 | 0.01 | Regulation of secondary cell wall biogenesis, regulation of transcription, DNA-dependent, response to abscisic acid stimulus, |
| GO246036 | GO246036 OEAA-070810_Plate8o21.b1 cDNA library from Olive leaves and fruits *Olea europaea* cDNA | –2.08 | 0.00 | Salicylic acid metabolic process |
| GO244411 | GO244411 OEAA-070810_Plate4i22.b1 cDNA library from Olive leaves and fruits *Olea europaea* cDNA | –2.08 | 0.01 | Unknown |
| GO244529 | GO244529 OEAA-070810_Plate4o02.b1 cDNA library from Olive leaves and fruits *Olea europaea* cDNA | –2.08 | 0.00 | Unknown |
| FL684180 | FL684180 A_E13_C07_0414C_p1 *Olea europaea* cv. Leccino fruitlet *Olea europaea* cDNA | –2.08 | 0.01 | Oxidation-reduction process, response to oxidative stress |
| GO245349 | GO245349 OEAA-070810_Plate7b14.b1 cDNA library from Olive leaves and fruits *Olea europaea* cDNA | –2.07 | 0.00 | Unknown |
| GO244768 | GO244768 OEAA-070810_Plate5i15.b1 cDNA library from Olive leaves and fruits *Olea europaea* cDNA | –2.07 | 0.02 | Unknown |
| GO245884 | GO245884 OEAA-070810_Plate8i09.b1 cDNA library from Olive leaves and fruits *Olea europaea* cDNA | –2.07 | 0.01 | Unknown |
| GO245986 | GO245986 OEAA-070810_Plate8m17.b1 cDNA library from Olive leaves and fruits *Olea europaea* cDNA | –2.07 | 0.01 | Calcium ion transport, cellular divalent inorganic cation homeostasis, monovalent inorganic cation transport, response to abiotic stimulus, response to chemical stimulus, response to stres, transmembrane transport, |
| GO244414 | GO244414 OEAA-070810_Plate4j01.b1 cDNA library from Olive leaves and fruits *Olea europaea* cDNA | –2.07 | 0.00 | Unknown |
| GO242873 | GO242873 OEAA-070810_Plate10h04.b1 cDNA library from Olive leaves and fruits *Olea europaea* cDNA | –2.07 | 0.01 | Transmembrane transport, response to abiotic stimulus, monovalent inorganic cation transport, response to chemical stimulus, calcium ion transport, cellular divalent inorganic cation homeostasis, response to stress |
| FN998086 | FN998086 FN998086 *Olea europaea* flower *Olea europaea* cDNA clone c1-6-A12 | –2.07 | 0.01 | Unknown |
| GO243735 | GO243735 OEAA-070810_Plate2m09.b1 cDNA library from Olive leaves and fruits *Olea europaea* cDNA | –2.06 | 0.00 | Unknown |
| gw1.12629.17.1 | gw1.12629.17.1: hypothetical protein SpolCp077 [Spinacia oleracea] | –2.06 | 0.00 | Unknown |
| GO243628 | GO243628 OEAA-070810_Plate2h14.b1 cDNA library from Olive leaves and fruits *Olea europaea* cDNA | –2.06 | 0.01 | Unknown |
| GO245883 | GO245883 OEAA-070810_Plate8i08.b1 cDNA library from Olive leaves and fruits *Olea europaea* cDNA | –2.05 | 0.01 | Lipid metabolic process |
| GO245380 | GO245380 OEAA-070810_Plate7c21.b1 cDNA library from Olive leaves and fruits *Olea europaea* cDNA | –2.05 | 0.00 | Unknown |
| GO242886 | GO242886 OEAA-070810_Plate10h17.b1 cDNA library from Olive leaves and fruits *Olea europaea* cDNA | –2.05 | 0.00 | Calcium ion transport, cellular divalent inorganic cation homeostasis, monovalent inorganic cation transport, response to abiotic stimulus, response to chemical stimulus, response to stres, transmembrane transport, |
| gw1.117.241.1 | protein kinase family protein | –2.05 | 0.01 | Protein phosphorylation, transferase activity |
| estExt_Genewise1_v1.C_LG_VI1605 | ATTDT, ATSDAT \| ATSDAT/ATTDT (TONOPLAST DICARBOXYLATE TRANSPORTER); malate transporter/ sodium:dicarboxylate symporter | –2.05 | 0.01 | Regulation of intracellular pH, malate transport, dicarboxylic acid transport, sodium ion transmembrane transport, sodium ion transport, transmembrane transport, sodium ion transport |
| GO244001 | GO244001 OEAA-070810_Plate3h17.b1 cDNA library from Olive leaves and fruits *Olea europaea* cDNA | –2.04 | 0.00 | Response to oxidative stress |
| GO243107 | GO243107 OEAA-070810_Plate1b01.b1 cDNA library from Olive leaves and fruits *Olea europaea* cDNA | –2.04 | 0.00 | Abscisic acid mediated signaling pathway, cellular response to water deprivation, oxidation-reduction process, response to hydrogen peroxide, toxin catabolic process |
| estExt_Genewise1_v1.C_LG_VI1322 | ATMAP70-5 \| ATMAP70-5 (microtubule-associated proteins 70-5); microtubule binding | –2.04 | 0.00 | Glucuronoxylan metabolic process, xylem and phloem pattern formation, plant-type cell wall biogenesis, cytoskeleton organization, xylan biosynthetic process |
| GO244546 | GO244546 OEAA-070810_Plate4o19.b1 cDNA library from Olive leaves and fruits *Olea europaea* cDNA | –2.04 | 0.01 | Pentose-phosphate shunt, protein phosphorylation, signal transduction, valine metabolic process, |
| eugene3.08370003 | SNF2 domain-containing protein / helicase domain-containing protein / zinc finger (C3HC4 type RING finger) family protein | –2.04 | 0.01 | Unknown |
| GO244958 | GO244958 OEAA-070810_Plate6a19.b1 cDNA library from Olive leaves and fruits *Olea europaea* cDNA | –2.04 | 0.00 | Unknown |
| GO243327 | GO243327 OEAA-070810_Plate1k16.b1 cDNA library from Olive leaves and fruits *Olea europaea* cDNA | –2.04 | 0.00 | Unknown |
| GO243075 | GO243075 OEAA-070810_Plate10p15.b1 cDNA library from Olive leaves and fruits *Olea europaea* cDNA | –2.03 | 0.02 | Transmembrane transport, response to abiotic stimulus, monovalent inorganic cation transport, response to chemical stimulus, calcium ion transport, cellular divalent inorganic cation homeostasis, response to stress |
| GO244045 | GO244045 OEAA-070810_Plate3j13.b1 cDNA library from Olive leaves and fruits *Olea europaea* cDNA | –2.03 | 0.01 | Unknown |
| FL684366 | FL684366 C_F09_C05_0414F_p7 *Olea europaea* cv. Leccino fruitlet *Olea europaea* cDNA | –2.03 | 0.00 | Unknown |
| GO244048 | GO244048 OEAA-070810_Plate3j16.b1 cDNA library from Olive leaves and fruits *Olea europaea* cDNA | –2.03 | 0.00 | Defense response to bacterium, incompatible interaction, protein folding, response to arsenic-containing substance, response to heat, response to high light intensity, response to hydrogen peroxide, |
| GO244126 | GO244126 OEAA-070810_Plate3m22.b1 cDNA library from Olive leaves and fruits *Olea europaea* cDNA | –2.03 | 0.00 | Ferredoxin metabolic process, photosynthetic electron transport chain, response to karrikin, response to light stimulus, |
| FL684161 | FL684161 A_G13_D07_0414F_p1 *Olea europaea* cv. Leccino fruitlet *Olea europaea* cDNA | –2.03 | 0.00 | Signal transduction |
| GO245966 | GO245966 OEAA-070810_Plate8l21.b1 cDNA library from Olive leaves and fruits *Olea europaea* cDNA | –2.02 | 0.00 | Unknown |
| GO242906 | GO242906 OEAA-070810_Plate10i13.b1 cDNA library from Olive leaves and fruits *Olea europaea* cDNA | –2.02 | 0.05 | Transmembrane transport, response to abiotic stimulus, monovalent inorganic cation transport, response to chemical stimulus, calcium ion transport, cellular divalent inorganic cation homeostasis, response to stress |
| gw1.261.3.1 | Kin17 DNA-binding protein-related | –2.02 | 0.01 | Microtubule cytoskeleton organization |
| estExt_fgenesh4_pm.C570006 | similar to unknown protein [Arabidopsis thaliana] (TAIR:AT1G52330.1) | –2.02 | 0.04 | Response to desiccation |
| GO246143 | GO246143 OEAA-070810_Plate9d09.b1 cDNA library from Olive leaves and fruits *Olea europaea* cDNA | –2.02 | 0.04 | Unknown |
| GO243269 | GO243269 OEAA-070810_Plate1i02.b1 cDNA library from Olive leaves and fruits *Olea europaea* cDNA | –2.01 | 0.01 | Unknown |
| GO244846 | GO244846 OEAA-070810_Plate5l23.b1 cDNA library from Olive leaves and fruits *Olea europaea* cDNA | –2.01 | 0.03 | Lipid metabolic process |
| GO244665 | GO244665 OEAA-070810_Plate5d24.b1 cDNA library from Olive leaves and fruits *Olea europaea* cDNA | –2.01 | 0.00 | Unknown |
| GO245208 | GO245208 OEAA-070810_Plate6l11.b1 cDNA library from Olive leaves and fruits *Olea europaea* cDNA | –2.01 | 0.01 | Unknown |
| GO245484 | GO245484 OEAA-070810_Plate7h09.b1 cDNA library from Olive leaves and fruits *Olea europaea* cDNA | –2.01 | 0.00 | Unknown |
| GO246079 | GO246079 OEAA-070810_Plate9a17.b1 cDNA library from Olive leaves and fruits *Olea europaea* cDNA | –2.01 | 0.00 | Calcium ion transport, cellular divalent inorganic cation homeostasis, monovalent inorganic cation transport, response to abiotic stimulus, response to chemical stimulus, response to stres, transmembrane transport, |
| GO242860 | GO242860 OEAA-070810_Plate10g15.b1 cDNA library from Olive leaves and fruits *Olea europaea* cDNA | –2.01 | 0.00 | Transmembrane transport, response to abiotic stimulus, monovalent inorganic cation transport, response to chemical stimulus, calcium ion transport, cellular divalent inorganic cation homeostasis, response to stress |
| GO243221 | GO243221 OEAA-070810_Plate1g01.b1 cDNA library from Olive leaves and fruits *Olea europaea* cDNA | –2.01 | 0.00 | Unknown |
| GO243189 | GO243189 OEAA-070810_Plate1e16.b1 cDNA library from Olive leaves and fruits *Olea europaea* cDNA | –2.00 | 0.01 | Cutin biosynthetic process, embryo development ending in seed dormancy, epidermis morphogenesis, localization, trichome morphogenesis, |
| gw1.III.1121.1 | pentatricopeptide (PPR) repeat-containing protein | –2.00 | 0.02 | Unknown |
| FN998199 | FN998199 FN998199 *Olea europaea* flower *Olea europaea* cDNA clone c1-7-C11 | –2.00 | 0.01 | Unknown |
| GO244975 | GO244975 OEAA-070810_Plate6b13.b1 cDNA library from Olive leaves and fruits *Olea europaea* cDNA | –2.00 | 0.00 | Reductive pentose-phosphate cycle, oxidation-reduction process, response to red light, response to cold, response to blue light, photorespiration, response to far red light |
| eugene3.00120398 | “ \| Symbols: TUB6 \| TUB6 (BETA-6 TUBULIN) \| chr5:3961318-3962972 REVERSE Length = 449 Score = 828 bits (2138), Expect = 0.0” | 2.00 | 0.00 | Proteasomal protein catabolic process, glycolysis, water transport, microtubule-based process, cytoskeleton organization, acetyl-CoA metabolic process, seed dormancy process, hyperosmotic response, multidimensional cell growth, cysteine biosynthetic proce |
| fgenesh4_pg.C_scaffold_676000002 | disease resistance protein (NBS-LRR class), putative | 2.01 | 0.00 | Defense response |
| gw1.I.4086.1 | ZAT10, STZ \| STZ (SALT TOLERANCE ZINC FINGER); nucleic acid binding / transcription factor/ zinc ion binding | 2.01 | 0.01 | Response to water deprivation, negative regulation of transcription, response to auxin stimulus, response to chitin, photoprotection, response to wounding, photosynthesis, response to ethylene stimulus, hyperosmotic salinity response, intracellular signalling transduction, response to oxidative stress, signal transduction, rsponse to high light intensity, response to salt stress, response to cold, rsponse to oxidative stress, response to abscisic acid stimulus, jasmonic acid biosynthetic process, rsponse to fungus, response to water deprivation, multicellular organism growth |
| eugene3.01960035 | similar to unknown protein [Arabidopsis thaliana] (TAIR:AT3G23910.1) | 2.01 | 0.00 | Unknown |
| fgenesh4_pg.C_scaffold9987000001 | LOX3 (Lipoxygenase 3); iron ion binding / lipoxygenase | 2.05 | 0.00 | Response to wounding, lipid oxidation, anther development, response to jasmonic acid stimulus, response to fungus, polen development, response to high light , instensity, defense response, anther dehiscence, jasmonic acid biosynthetic process, ethylene biosynthetic process, growth, rsponse to chitin, stamen filament development |
| estExt_Genewise1_v1.C_LG_IV4530 | transmembrane CLPTM1 family protein | 2.06 | 0.02 | Reciprocal meiotic recombination, response to gamma radiation, meiotic DNA double-strand break formation, sister chromatid cohesion, regulation of telomere maintenance, telomere maintenance in response to DNA damage, regulation of chromosome organization, multicellular organism reproduction, synapsis, meiotic chromosome segregation, protein transport, N-terminal protein myristoylation |
| grail3.0047019501 | TUB6 \| TUB6 (BETA-6 TUBULIN) | 2.06 | 0.00 | Regulation of hormone levels, response to cadmium ion, microtubule-based process, response to temperature stimulus, glycolysis, multidimensional cell growth, cell wall organization, gibberellic acid mediated signaling pathway, Golgi organization, response to salt stress, anthocyanin accumulation in tissues in response to UV light, cell tip growth, brassinosteroid biosynthetic process, hyperosmotic response, response to cold, gluconeogenesis, root hair elongation, cytoskeleton organization, sterol biosynthetic process, proteasomal protein catabolic process, cysteine biosynthetic process, seed dormancy process, acetyl-CoA metabolic process, water transport, polysaccharide biosynthetic process, response to salt stress, GTP catabolic process, microtubule-based movement, microtubule-based process, protein polymerization |
| GO244795 | GO244795 OEAA-070810_Plate5j18.b1 cDNA library from Olive leaves and fruits *Olea europaea* cDNA | 2.11 | 0.01 | Proteolysis |
| fgenesh1_pg.C_scaffold_5520000001 | protein kinase family protein | 2.12 | 0.00 | Protein phosphorylation, transferase activity |
| estExt_Genewise1_v1.C_LG_III1447 | similar to unknown protein [Arabidopsis thaliana] (TAIR:AT5G22790.1) | 2.12 | 0.00 | Unknown |
| eugene3.00021641 | eugene3.00021641: putative MYB-like transcription factor [Oryza sativa (japonica cultivar-group)] | 2.12 | 0.01 | Unknown |
| estExt_fgenesh4_pg.C_LG_XI0828 | IQD9 \| IQD9 (IQ-domain 9); calmodulin binding | 2.14 | 0.00 | Methionine biosynthetic pathway, photoperiodism, flowering |
| gw1.XIX.504.1 | PPO, TOPP2 \| TOPP2 (Type one serine/threonine protein phosphatase 2) | 2.15 | 0.02 | Vegetative to reproductive phase transition of meristem, protein dephosphorylation, hydrogen peroxide biosynthetic process, protein desumoylation |
| fgenesh4_pg.C_LG_XII000710 | fgenesh4_pg.C_LG_XII000710: RING-finger protein [Helicoverpa armigera nuclear polyhedrosis virus] | 2.16 | 0.00 | Unknown |
| gw1.VI.1838.1 | ATWRKY49, WRKY49 \| WRKY49 (WRKY DNA-binding protein 49); transcription factor | 2.17 | 0.03 | Regulation of transcription |
| estExt_Genewise1_v1.C650071 | DNA-directed RNA polymerase (RPOT2) | 2.18 | 0.00 | Transcription |
| GO245268 | GO245268 OEAA-070810_Plate6o02.b1 cDNA library from Olive leaves and fruits *Olea europaea* cDNA | 2.21 | 0.00 | Response to cold, maltose biosynthetic process, starch catabolic process |
| eugene3.01650006 | pollen Ole e 1 allergen and extensin family protein | 2.22 | 0.01 | Unknown |
| eugene3.00101099 | MATE efflux family protein | 2.23 | 0.00 | Drug transmembrane transport, transmembrane transport |
| FN998573 | FN998573 FN998573 *Olea europaea* flower *Olea europaea* cDNA clone c2-4-A9 | 2.25 | 0.00 | Unknown |
| estExt_Genewise1_v1.C_7600034 | hypothetical protein NitaMp079 [Nicotiana tabacum] | 2.28 | 0.03 | Unknown |
| eugene3.49050001 | eugene3.49050001: unnamed protein product [Rattus rattus] | 2.30 | 0.01 | Unknown |
| FL683503 | FL683503 B_E13_C07_0414F_p5 *Olea europaea* cv. Leccino fruitlet *Olea europaea* cDNA | 2.31 | 0.00 | Unknown |
| eugene3.00110007 | eugene3.00110007: putative taxane 14b-hydroxylase [Oryza sativa (japonica cultivar-group)] | 2.33 | 0.02 | Unknown |
| estExt_fgenesh4_pg.C_LG_VI1748 | proteasome maturation factor UMP1 family protein | 2.34 | 0.00 | Unknown |
| fgenesh4_pg.C_LG_VI000379 | similar to Os11g0140100 [Oryza sativa (japonica cultivar-group)] (GB:NP_001065708.1) | 2.37 | 0.00 | Unknown |
| GO243458 | GO243458 OEAA-070810_Plate2a05.b1 cDNA library from Olive leaves and fruits *Olea europaea* cDNA | 2.43 | 0.01 | Multidimensional cell growth, regulation of transcription, DNA-dependent |
| gw1.162.7.1 | similar to unknown protein [Arabidopsis thaliana] (TAIR:AT1G43722.1); similar to Transposase, IS4 [Medicago truncatula] (GB:ABE80314.1) | 2.49 | 0.01 | Hydrolase activity |
| FN997782 | FN997782 FN997782 *Olea europaea* flower *Olea europaea* cDNA clone c1-2-E3 | 2.53 | 0.01 | Response to karrikin |
| fgenesh4_pg.C_LG_X001297 | BG1 \| BG1 (BETA-1,3-GLUCANASE 1); hydrolase, hydrolyzing O-glycosyl compounds | 2.53 | 0.00 | Hydrolase activity, carbohydrate metabolic process, hydrolase activity, cell morphogenesis, Golgi vesicle transport, unknown, cell growth |
| eugene3.00141311 | eugene3.00141311: ORF [Lilium longiflorum] | 2.54 | 0.02 | Unknown |
| grail3.0001090401 | ELI3-1 \| ELI3-1 (ELICITOR-ACTIVATED GENE 3); oxidoreductase/ zinc ion binding | 2.57 | 0.04 | Plant-type hypersensitive response, response to bacterium, lignin biosynthetic process, oxidation-reduction process, transferase activity, oxidation-reduction process |
| GO243444 | GO243444 OEAA-070810_Plate1p14.b1 cDNA library from Olive leaves and fruits *Olea europaea* cDNA | 2.58 | 0.00 | Unknown |
| FL684059 | FL684059 D_J04_E02_0414F_p12 *Olea europaea* cv. Leccino fruitlet *Olea europaea* cDNA | 2.59 | 0.00 | Oxidation-reduction process |
| grail3.0032017101 | similar to dioxygenase. [ORG:Marah macrocarpus] | 2.63 | 0.02 | Oxidoreductase activity |
| eugene3.00160596 | RHF2A, CIC7E11 \| CIC7E11; protein binding / zinc ion binding | 2.68 | 0.02 | Regulation of cell cycle, signal transduction, fatty acid beta-oxidation, megagametogenesis, proteolysis involved in cellular protein catabolic process, autophagy, protein import into peroxisome matrix, microgametogenesis, systemic acquired resistance, hormone-mediated signaling pathway, salicylic acid biosynthetic process, cellular macromolecule catabolic process |
| GO245913 | GO245913 OEAA-070810_Plate8j14.b1 cDNA library from Olive leaves and fruits *Olea europaea* cDNA | 2.82 | 0.00 | Regulation of meristem growth, carbohydrate metabolic process |
| GO244789 | GO244789 OEAA-070810_Plate5j12.b1 cDNA library from Olive leaves and fruits *Olea europaea* cDNA | 2.84 | 0.00 | Unknown |
| GO243651 | GO243651 OEAA-070810_Plate2i13.b1 cDNA library from Olive leaves and fruits *Olea europaea* cDNA | 2.92 | 0.00 | Proteolysis |
| eugene3.00102522 | 60S acidic ribosomal protein P2 (RPP2A) | 3.02 | 0.00 | Response to cold, translationale regulation |
| grail3.7095000201 | grail3.7095000201: putative salivary protein [Culicoides sonorensis] | 3.03 | 0.00 | Unknown |
| gw1.I.375.1 | protein kinase family protein | 3.07 | 0.00 | Protein phosphorylation |
| estExt_fgenesh4_pg.C_LG_II2533 | tyrosine decarboxylase, putative | 3.19 | 0.00 | Carboxylic acid metabolic process, response to wounding, cellular amino acid metabolic process |
| eugene3.137050001 | GDSL-motif lipase/hydrolase family protein | 3.36 | 0.02 | Cellular response to water deprivation, lipid metabolic process, cuticle development |
| fgenesh4_pg.C_scaffold_40000175 | methyltransferase MT-A70 family protein | 3.66 | 0.00 | Nucleobase-containing compound metabolic process, methylation |
| eugene3.00170500 | similar to unknown protein [Arabidopsis thaliana] (TAIR:AT1G13740.1) | 3.69 | 0.00 | unknown, abscisic acid mediated signaling pathway, positive regulation of transcription |
| eugene3.00020895 | protein phosphatase 2C, putative / PP2C, putative | 3.78 | 0.01 | Response to molecule of bacterial origin, ethylene biosynthetic process, red light signalling pathway, cellular membrane fusion, protein dephosphorylation |
| grail3.0039017801 | AP2 domain-containing transcription factor, putative | 3.79 | 0.02 | Response to abscisic acid response, response to cold, regulation of transcription-DNA dependent, response to water deprivation |
| GO245535 | GO245535 OEAA-070810_Plate7j13.b1 cDNA library from Olive leaves and fruits *Olea europaea* cDNA | 3.79 | 0.00 | Response to karrikin |
| eugene3.86990001 | eugene3.86990001: putative non-LTR retroelement reverse transcriptase [Arabidopsis thaliana] | 4.15 | 0.00 | Unknown |
| fgenesh4_pg.C_scaffold_5695000001 | fgenesh4_pg.C_scaffold_5695000001: RAS-related protein racD | 4.15 | 0.00 | Unknown |
| eugene3.00170228 | eugene3.00170228: unnamed protein product [Arabidopsis thaliana] | 4.29 | 0.00 | Unknown |
| estExt_Genewise1_v1.C_660290 | ATBPM4 \| ATBPM4 (BTB-POZ AND MATH DOMAIN 4); protein binding | 4.30 | 0.00 | Cellular response to water deprivation, cellular response to salt stress, response to osmotic stress |
| eugene3.00290281 | UGT73B2 \| UGT73B2; UDP-glucosyltransferase/ UDP-glycosyltransferase/ flavonol 3-O-glucosyltransferase | 4.31 | 0.01 | Flavonol biosynthetic process, response to other organism, metabolic process, transferase activity, metabolic process, transferase activity |
| estExt_Genewise1_v1.C_LG_XII0166 | ATPase | 4.46 | 0.00 | ATP binding |
| eugene3.01250091 | SNF2 domain-containing protein / helicase domain-containing protein / zinc finger (C3HC4 type RING finger) family protein | 4.57 | 0.00 | Unknown |
| fgenesh4_pm.C_LG_IV000224 | leucine-rich repeat transmembrane protein kinase, putative | 4.64 | 0.00 | Methylation dependent chromatin silencing, protein phosphorylation, RNA interference, petal formation, sepal formation, transmembrane receptor protein tyrosine kinase signalling pathway |
| fgenesh4_pg.C_LG_XVII000057 | RAB GDP-dissociation inhibitor | 4.99 | 0.01 | N-terminal protein myristoylation, protein transport |
| gw1.123.49.1 | COL2 \| COL2 (CONSTANS-LIKE 2); transcription factor/ zinc ion binding | 5.08 | 0.00 | Regulation of flower development, chloroplast organization |
| eugene3.02430009 | eugene3.02430009: hypothetical protein Bcep02001879 [Burkholderia fungorum LB400] | 6.52 | 0.00 | Unknown |
| **Seq_ID** | **Description** | **OFF-M /OFF-J Fold Change** | ***P* value** | **GO Biological Process** |
| GO245304 | GO245304 OEAA-070810_Plate6p15.b1 cDNA library from Olive leaves and fruits *Olea europaea* cDNA | –3.01 | 0 | Response to karrikin, syncytium formation |
| grail3.7874000101 | grail3.7874000101: Ig epsilon chain C region | –2.85 | 0 | Unknown |
| GO244529 | GO244529 OEAA-070810_Plate4o02.b1 cDNA library from Olive leaves and fruits *Olea europaea* cDNA | –2.79 | 0 | Unknown |
| eugene3.00140935 | eugene3.00140935: putative protein kinase [Arabidopsis thaliana] | –2.72 | 0 | Unknown |
| eugene3.01420101 | DNA cross-link repair protein-related | –2.68 | 0 | Unknown |
| GO243777 | GO243777 OEAA-070810_Plate2o06.b1 cDNA library from Olive leaves and fruits *Olea europaea* cDNA | –2.67 | 0 | Type I hypersensitivity |
| FL684184 | FL684184 C_D12_B06_0414F_p8 *Olea europaea* cv. Leccino fruitlet *Olea europaea* cDNA | –2.65 | 0 | Heat acclimation, protein folding, protein unfolding, response to endoplasmic reticulum stress, response to high light intensity, response to hydrogen peroxide |
| FL683438 | FL683438 D_K16_F08_0414F_p14 *Olea europaea* cv. Leccino fruitlet *Olea europaea* cDNA | –2.64 | 0 | Unknown |
| GO245518 | GO245518 OEAA-070810_Plate7i20.b1 cDNA library from Olive leaves and fruits *Olea europaea* cDNA | –2.63 | 0 | Regulation of secondary cell wall biogenesis, regulation of transcription, DNA-dependent, response to abscisic acid stimulus |
| GO244140 | GO244140 OEAA-070810_Plate3n12.b1 cDNA library from Olive leaves and fruits *Olea europaea* cDNA | –2.62 | 0.01 | Fatty acid metabolic process, oxidation-reduction process, carbohydrate metabolic process, defense response, response to bacterium, response to cold |
| GO245517 | GO245517 OEAA-070810_Plate7i19.b1 cDNA library from Olive leaves and fruits *Olea europaea* cDNA | –2.55 | 0 | Regulation of secondary cell wall biogenesis, regulation of transcription, DNA-dependent, response to abscisic acid stimulus |
| GO244729 | GO244729 OEAA-070810_Plate5g22.b1 cDNA library from Olive leaves and fruits *Olea europaea* cDNA | –2.48 | 0 | Cell wall biogenesis, cysteine biosynthetic process, trichome morphogenesis |
| GO245145 | GO245145 OEAA-070810_Plate6i18.b1 cDNA library from Olive leaves and fruits *Olea europaea* cDNA | –2.46 | 0 | Cell wall biogenesis, trichome morphogenesis |
| GO243932 | GO243932 OEAA-070810_Plate3e19.b1 cDNA library from Olive leaves and fruits *Olea europaea* cDNA | –2.44 | 0.01 | Unknown |
| gw1.VII.2355.1 | SAG12 \| SAG12 (SENESCENCE-ASSOCIATED GENE 12) | –2.37 | 0.03 | Aging, response to ethylene stimulus, leaf senescence, proteolysis, defense response to fungus, incompatible interaction |
| grail3.0017030801 | S2P-like putative metalloprotease | –2.33 | 0.04 | Response to hydrogen peroxide, response to heat, response to high light intensity, protein folding |
| eugene3.00101184 | CER6, G2, POP1, CUT1 \| CUT1 (CUTICULAR 1) | –2.31 | 0.01 | Fatty acid biosynthetic process, transferase activity, transferring acyl groups other than amino-acyl groups |
| GO243991 | GO243991 OEAA-070810_Plate3h07.b1 cDNA library from Olive leaves and fruits *Olea europaea* cDNA | –2.31 | 0.01 | Fatty acid metabolic process, oxidation-reduction process |
| GO245492 | GO245492 OEAA-070810_Plate7h17.b1 cDNA library from Olive leaves and fruits *Olea europaea* cDNA | –2.26 | 0 | Unknown |
| gw1.9592.1.1 | CAS1 \| CAS1 (CYCLOARTENOL SYNTHASE 1) | –2.21 | 0.01 | pentacyclic triterpenoid biosynthetic process, pollen development, pentacyclic triterpenoid biosynthetic process, unsaturated fatty acid biosynthetic process, phosphatidylglycerol biosynthetic process, thylakoid membrane organization, isopentenyl diphosph |
| fgenesh4_pg.C_scaffold_44000037 | pentatricopeptide (PPR) repeat-containing protein | –2.17 | 0.01 | Unknown |
| gw1.21597.3.1 | gw1.21597.3.1: unnamed protein product [Kluyveromyces lactis] | –2.15 | 0.01 | Unknown |
| fgenesh4_pg.C_LG_V000279 | fgenesh4_pg.C_LG_V000279: ABC TRANSPORT SYSTEM ATP-BINDING PROTEIN [Wolinella succinogenes DSM 1740] | –2.14 | 0.01 | Unknown |
| gw1.II.1879.1 | pfkB-type carbohydrate kinase family protein | –2.1 | 0 | Kinase activity, D-ribose catabolic processes |
| GO243814 | GO243814 OEAA-070810_Plate2p19.b1 cDNA library from Olive leaves and fruits *Olea europaea* cDNA | –2.08 | 0 | Defense response, response to biotic stimulus |
| GO243211 | GO243211 OEAA-070810_Plate1f15.b1 cDNA library from Olive leaves and fruits *Olea europaea* cDNA | –2.08 | 0 | Defense response, response to biotic stimulus |
| FN998508 | FN998508 FN998508 *Olea europaea* flower *Olea europaea* cDNA clone c2-3-D2 | –2.08 | 0 | Mucilage extrusion from seed coat, mucilage metabolic process involved seed coat development, negative regulation of catalytic activity, proteolysis |
| eugene3.46710002 |  | –2.07 | 0 | Unknown |
| GO246183 | GO246183 OEAA-070810_Plate9f01.b1 cDNA library from Olive leaves and fruits *Olea europaea* cDNA | –2.06 | 0.01 | Unknown |
| eugene3.00161233 | binding | –2.01 | 0.04 | Unknown |
| GO244599 | GO244599 OEAA-070810_Plate5b03.b1 cDNA library from Olive leaves and fruits *Olea europaea* cDNA | –2 | 0 | Urea transmembrane transport, water transport |
| FN998333 | FN998333 FN998333 *Olea europaea* flower *Olea europaea* cDNA clone c2-1-B4 | 2 | 0.04 | Carbohydrate metabolic process |
| FN998334 | FN998334 FN998334 *Olea europaea* flower *Olea europaea* cDNA clone c2-1-B5 | 2 | 0.02 | Carbohydrate metabolic process |
| GO245994 | GO245994 OEAA-070810_Plate8n01.b1 cDNA library from Olive leaves and fruits *Olea europaea* cDNA | 2.01 | 0.03 | Jasmonic acid biosynthetic process, protein targeting to vacuole, response to wounding |
| FN998032 | FN998032 FN998032 *Olea europaea* flower *Olea europaea* cDNA clone c1-5-D6 | 2.01 | 0 | Unknown |
| FN998396 | FN998396 FN998396 *Olea europaea* flower *Olea europaea* cDNA clone c2-2-A5 | 2.01 | 0.01 | Carbohydrate metabolic process |
| FN998287 | FN998287 FN998287 *Olea europaea* flower *Olea europaea* cDNA clone c2-5-C2 | 2.02 | 0.04 | Carbohydrate metabolic process |
| FN998302 | FN998302 FN998302 *Olea europaea* flower *Olea europaea* cDNA clone c2-5-D9 | 2.03 | 0.01 | Carbohydrate metabolic process |
| GO243438 | GO243438 OEAA-070810_Plate1p08.b1 cDNA library from Olive leaves and fruits *Olea europaea* cDNA | 2.04 | 0 | Defense response to bacterium, protein autoubiquitination, regulation of transport, multidimensional cell growth, regulation of transcription, DNA-dependent |
| FN997722 | FN997722 FN997722 *Olea europaea* flower *Olea europaea* cDNA clone c1-1-F7 | 2.05 | 0.02 | Cellular membrane fusion, endosperm development, negative regulation of programmed cell death, N-terminal protein myristoylation, protein autophosphorylation, protein targeting to membrane, proteolysis, regulation of plant-type hypersensitive response, response to molecule of bacterial origin, response to oxidative stress, seed germination, stamen development, stomatal complex morphogenesis, systemic acquired resistance, transmembrane receptor protein tyrosine kinase signaling pathway |
| GO244287 | GO244287 OEAA-070810_Plate4d17.b1 cDNA library from Olive leaves and fruits *Olea europaea* cDNA | 2.05 | 0.02 | Unknown |
| GO245048 | GO245048 OEAA-070810_Plate6e15.b1 cDNA library from Olive leaves and fruits *Olea europaea* cDNA | 2.05 | 0 | Unknown |
| GO244484 | GO244484 OEAA-070810_Plate4m03.b1 cDNA library from Olive leaves and fruits *Olea europaea* cDNA | 2.05 | 0 | Isoprenoid biosynthetic process |
| FN998437 | FN998437 FN998437 *Olea europaea* flower *Olea europaea* cDNA clone c2-2-E5 | 2.06 | 0.02 | Carbohydrate metabolic process |
| GO245562 | GO245562 OEAA-070810_Plate7k16.b1 cDNA library from Olive leaves and fruits *Olea europaea* cDNA | 2.07 | 0.02 | Aromatic amino acid family biosynthetic process, cell differentiation, chlorophyll biosynthetic process, cysteine biosynthetic process, defense response to bacterium, defense response, incompatible interaction, isopentenyl diphosphate biosynthetic process, mevalonate-independent pathway, leaf morphogenesis, pentose-phosphate shunt, peptidyl-pyrromethane cofactor linkage, photosynthesis, light reaction, positive regulation of transcription, DNA-dependent, response to cold, rRNA processing, salicylic acid biosynthetic process, ubiquinone biosynthetic process |
| FN998830 | FN998830 FN998830 *Olea europaea* flower *Olea europaea* cDNA clone c2-7-H12 | 2.07 | 0.01 | Carbohydrate metabolic process |
| FN998331 | FN998331 FN998331 *Olea europaea* flower *Olea europaea* cDNA clone c2-5-H8 | 2.08 | 0.02 | Carbohydrate metabolic process |
| FN998683 | FN998683 FN998683 *Olea europaea* flower *Olea europaea* cDNA clone c2-6-C3 | 2.08 | 0.02 | Carbohydrate metabolic process |
| GO244150 | GO244150 OEAA-070810_Plate3n22.b1 cDNA library from Olive leaves and fruits *Olea europaea* cDNA | 2.11 | 0.01 | Unknown |
| FN998606 | FN998606 FN998606 *Olea europaea* flower *Olea europaea* cDNA clone c2-4-D6 | 2.13 | 0.05 | Carbohydrate metabolic process |
| FN997799 | FN997799 FN997799 *Olea europaea* flower *Olea europaea* cDNA clone c1-2-G1 | 2.14 | 0.03 | Carbohydrate metabolic process |
| FN998329 | FN998329 FN998329 *Olea europaea* flower *Olea europaea* cDNA clone c2-5-H6 | 2.14 | 0.03 | Carbohydrate metabolic process |
| eugene3.00081119 | ATXYL1/XYL1 (ALPHA-XYLOSIDASE 1) | 2.15 | 0.02 | Response to cadmium ion, starch metabolic process, cell wall organisation, xyloglucan metabolic process, cell wall biogenesis, carbohydrate metabolic process, polysacharide catabolic process, xylan catabolic process |
| gw1.II.3432.1 | MYB55 (myb domain protein 55) | 2.15 | 0 | Regulation of transcription |
| gw1.XV.1199.1 | trigger factor type chaperone family protein | 2.15 | 0.01 | Carotenoid biosynthetic process, stomatal complex morphogenesis, tyhlakoid membrane organisation, protein folding, starch biosynthetic process, protein transport, mRNA modification, maltose metabolic process, chloroplast relocation |
| grail3.0096003001 | grail3.0096003001: late embryogenesis abundant protein [Picea glauca] | 2.16 | 0.02 | Response to stress |
| GO244921 | GO244921 OEAA-070810_Plate5p04.b1 cDNA library from Olive leaves and fruits *Olea europaea* cDNA | 2.16 | 0.04 | Unknown |
| FN998263 | FN998263 FN998263 *Olea europaea* flower *Olea europaea* cDNA clone c2-5-A10 | 2.17 | 0.02 | Carbohydrate metabolic process |
| GO244440 | GO244440 OEAA-070810_Plate4k05.b1 cDNA library from Olive leaves and fruits *Olea europaea* cDNA | 2.17 | 0 | Carbohydrate metabolic process, defense response, response to bacterium, response to cold |
| FN998586 | FN998586 FN998586 *Olea europaea* flower *Olea europaea* cDNA clone c2-4-C1 | 2.2 | 0.03 | Carbohydrate metabolic process |
| fgenesh4_pg.C_scaffold_9882000001 | ATTPS1 (TREHALOSE-6-PHOSPHATE SYNTHASE) | 2.2 | 0 | Embryo development ending in seed dormancy, cell wall biogenesis, trehalose metabolic process |
| grail3.0012038601 | AFH1 (FORMIN HOMOLOGY 1) | 2.2 | 0.01 | Growth, response to UV-light, actin cytosceleton organisation, polysaccharide biosynthetic process, regulation of hormone, cell tip growth, cell wall organisation |
| FN998444 | FN998444 FN998444 *Olea europaea* flower *Olea europaea* cDNA clone c2-2-F11 | 2.21 | 0.04 | Carbohydrate metabolic process |
| GO243251 | GO243251 OEAA-070810_Plate1h07.b1 cDNA library from Olive leaves and fruits *Olea europaea* cDNA | 2.22 | 0 | Response to karrikin, response to nematode, response to zinc ion |
| GO243143 | GO243143 OEAA-070810_Plate1c14.b1 cDNA library from Olive leaves and fruits *Olea europaea* cDNA | 2.22 | 0.02 | Megative regulation of abscisic acid mediated signaling pathway, regulation of cell size, regulation of transcription, DNA-dependent, response to fungus, response to hypoxia, response to jasmonic acid stimulus, response to light stimulus, response to salicylic acid stimulus, response to sucrose stimulus, response to wounding |
| GO243266 | GO243266 OEAA-070810_Plate1h23.b1 cDNA library from Olive leaves and fruits *Olea europaea* cDNA | 2.22 | 0.01 | Unknown |
| GO244708 | GO244708 OEAA-070810_Plate5f21.b1 cDNA library from Olive leaves and fruits *Olea europaea* cDNA | 2.22 | 0.03 | Chlorophyll biosynthetic process |
| FN998380 | FN998380 FN998380 *Olea europaea* flower *Olea europaea* cDNA clone c2-1-H1 | 2.23 | 0.01 | Carbohydrate metabolic process |
| gw1.66.228.1 | WAKL1 (WALL ASSOCIATED KINASE-LIKE 1) | 2.23 | 0.01 | Protein phosphorylation |
| FN998762 | FN998762 FN998762 *Olea europaea* flower *Olea europaea* cDNA clone c2-7-B11 | 2.23 | 0.01 | Unknown |
| FN998314 | FN998314 FN998314 *Olea europaea* flower *Olea europaea* cDNA clone c2-5-F12 | 2.24 | 0.03 | Carbohydrate metabolic process |
| FN997783 | FN997783 FN997783 *Olea europaea* flower *Olea europaea* cDNA clone c1-2-E5 | 2.24 | 0.05 | Unknown |
| fgenesh4_pm.C_scaffold_164000013 | ATMRP3 (Arabidopsis thaliana multidrug resistance-associated protein 3) | 2.25 | 0 | Transmembrane transport |
| FN998785 | FN998785 FN998785 *Olea europaea* flower *Olea europaea* cDNA clone c2-7-D2 | 2.26 | 0.04 | Carbohydrate metabolic process |
| eugene3.00111276 | oxidoreductase, 2OG-Fe(II) oxygenase family protein | 2.28 | 0.01 | Secondary metabolic process, oxidation-reduction process |
| GO244193 | GO244193 OEAA-070810_Plate3p18.b1 cDNA library from Olive leaves and fruits *Olea europaea* cDNA | 2.28 | 0.02 | Unknown |
| eugene3.00011170 | hypothetical protein [Vitis vinifera] | 2.29 | 0 | Unknown |
| GO243680 | GO243680 OEAA-070810_Plate2j19.b1 cDNA library from Olive leaves and fruits *Olea europaea* cDNA | 2.3 | 0.02 | Methylglyoxal catabolic process to D-lactate, N-terminal protein amino acid modification, proteolysis |
| GO243658 | GO243658 OEAA-070810_Plate2i21.b1 cDNA library from Olive leaves and fruits *Olea europaea* cDNA | 2.3 | 0 | Glutamate metabolic process, nitrate transport, regulation of anion channel activity, response to cadmium ion, response to nitrate, root hair elongation, transition metal ion transport, |
| FL684269 | FL684269 A_O07_H04_0414F_p1 *Olea europaea* cv. Leccino fruitlet *Olea europaea* cDNA | 2.31 | 0.01 | Unknown |
| FN998404 | FN998404 FN998404 *Olea europaea* flower *Olea europaea* cDNA clone c2-2-B3 | 2.31 | 0.01 | Carbohydrate metabolic process |
| FN998832 | FN998832 FN998832 *Olea europaea* flower *Olea europaea* cDNA clone c2-7-H3 | 2.32 | 0.01 | Carbohydrate metabolic process |
| FN998550 | FN998550 FN998550 *Olea europaea* flower *Olea europaea* cDNA clone c2-3-G9 | 2.33 | 0 | Carbohydrate metabolic process |
| GO245948 | GO245948 OEAA-070810_Plate8l02.b1 cDNA library from Olive leaves and fruits *Olea europaea* cDNA | 2.33 | 0.04 | Unknown |
| FL684259 | FL684259 C_F20_C10_0414F_p8 *Olea europaea* cv. Leccino fruitlet *Olea europaea* cDNA | 2.33 | 0.04 | Phosphorylation |
| GO245678 | GO245678 OEAA-070810_Plate7p14.b1 cDNA library from Olive leaves and fruits *Olea europaea* cDNA | 2.36 | 0.01 | Oxidation-reduction process, response to oxidative stress, response to salt stress, trichoblast differentiation |
| gw1.IV.4047.1 | 30S ribosomal protein, putative | 2.36 | 0.01 | Metabolic process, translation |
| GO244346 | GO244346 OEAA-070810_Plate4g04.b1 cDNA library from Olive leaves and fruits *Olea europaea* cDNA | 2.37 | 0.01 | Unknown |
| FN998798 | FN998798 FN998798 *Olea europaea* flower *Olea europaea* cDNA clone c2-7-E4 | 2.39 | 0 | Carbohydrate metabolic process |
| FN998522 | FN998522 FN998522 *Olea europaea* flower *Olea europaea* cDNA clone c2-3-E4 | 2.43 | 0.01 | Carbohydrate metabolic process |
| GO244837 | GO244837 OEAA-070810_Plate5l13.b1 cDNA library from Olive leaves and fruits *Olea europaea* cDNA | 2.43 | 0.05 | DNA-dependent DNA replication initiation, lagging strand elongation |
| FN998318 | FN998318 FN998318 *Olea europaea* flower *Olea europaea* cDNA clone c2-5-F8 | 2.43 | 0.04 | Carbohydrate metabolic process |
| FL684398 | FL684398 D_D16_B08_0414F_p12 *Olea europaea* cv. Leccino fruitlet *Olea europaea* cDNA | 2.47 | 0 | Unknown |
| GO243458 | GO243458 OEAA-070810_Plate2a05.b1 cDNA library from Olive leaves and fruits *Olea europaea* cDNA | 2.47 | 0 | Multidimensional cell growth, regulation of transcription, DNA-dependent |
| GO245657 | GO245657 OEAA-070810_Plate7o17.b1 cDNA library from Olive leaves and fruits *Olea europaea* cDNA | 2.48 | 0.02 | Cellular process, embryo development ending in seed dormancy, protein metabolic process |
| GO243501 | GO243501 OEAA-070810_Plate2c01.b1 cDNA library from Olive leaves and fruits *Olea europaea* cDNA | 2.48 | 0 | Cellular response to iron ion starvation, cellulose biosynthetic process, copper ion transmembrane transport, Golgi vesicle transport, gravitropism, iron ion transmembrane transport, iron ion transmembrane transport, oxidation-reduction process, protein targeting to vacuole, response to nematode, response to zinc ion, water transport, zinc ion transmembrane transport |
| FN998407 | FN998407 FN998407 *Olea europaea* flower *Olea europaea* cDNA clone c2-2-B6 | 2.48 | 0.04 | Carbohydrate metabolic process |
| FL683809 | FL683809 A_I22_E11_0414F_p2 *Olea europaea* cv. Leccino fruitlet *Olea europaea* cDNA | 2.49 | 0 | Unknown |
| fgenesh4_pg.C_scaffold_18930000001 | acidic endochitinase (CHIB1) | 2.49 | 0.01 | Response to cold, response to salt sress, response to water deprivation, response to wounding, protein desumoylation, vegetative to reproductive phase transition, carbohydrate metabolic process |
| FN997987 | FN997987 FN997987 *Olea europaea* flower *Olea europaea* cDNA clone c1-4-H9 | 2.49 | 0.01 | Activation of MAPKK activity, MAPK cascade, response to cadmium ion, response to cold, response to salt stress, response to wounding |
| FN998554 | FN998554 FN998554 *Olea europaea* flower *Olea europaea* cDNA clone c2-3-H12 | 2.49 | 0.01 | Carbohydrate metabolic process |
| fgenesh4_pg.C_scaffold_17272000001 | ATMCPB1, MCP1B, AMC1, LOL3 \| LOL3 (LSD ONE LIKE 3) | 2.49 | 0.01 | Proteolysis, regulation of hypersensitive response, negative regulation of defense response, protein targeting to membrane, salicylic acid biosynthetic process |
| gw1.XIX.158.1 | disease resistance protein (CC-NBS-LRR class) | 2.5 | 0.01 | Defense response, N-terminal protein myristoylation |
| FN998670 | FN998670 FN998670 *Olea europaea* flower *Olea europaea* cDNA clone c2-6-B10 | 2.5 | 0.02 | Carbohydrate metabolic process |
| FN998537 | FN998537 FN998537 *Olea europaea* flower *Olea europaea* cDNA clone c2-3-F8 | 2.5 | 0.02 | Carbohydrate metabolic process |
| gw1.I.7034.1 | thioredoxin family protein | 2.52 | 0.01 | Chloroplast organisation, t-RNA metabolic process, redox homeostasis, plastid organisation, positive regulation of transcription |
| FN998378 | FN998378 FN998378 *Olea europaea* flower *Olea europaea* cDNA clone c2-1-G8 | 2.54 | 0.02 | Carbohydrate metabolic process |
| FN998614 | FN998614 FN998614 *Olea europaea* flower *Olea europaea* cDNA clone c2-4-E2 | 2.55 | 0.04 | Carbohydrate metabolic process |
| GO244712 | GO244712 OEAA-070810_Plate5g01.b1 cDNA library from Olive leaves and fruits *Olea europaea* cDNA | 2.56 | 0.03 | Unknown |
| FN997716 | FN997716 FN997716 *Olea europaea* flower *Olea europaea* cDNA clone c1-1-F10 | 2.57 | 0.01 | Unknown |
| GO243444 | GO243444 OEAA-070810_Plate1p14.b1 cDNA library from Olive leaves and fruits *Olea europaea* cDNA | 2.57 | 0 | Unknown |
| gw1.XII.30.1 | phosphoethanolamine N-methyltransferase 2, putative (NMT2) | 2.59 | 0.04 | Metabolic process, maltose metabolic process, starch biosynthetic process, phosphatidylcholine biosynthetic process, positive regulation of catalytic activity, polyamine catabolic process |
| FN998351 | FN998351 FN998351 *Olea europaea* flower *Olea europaea* cDNA clone c2-1-D9 | 2.6 | 0.01 | Carbohydrate metabolic process |
| FN998301 | FN998301 FN998301 *Olea europaea* flower *Olea europaea* cDNA clone c2-5-D8 | 2.61 | 0.01 | Carbohydrate metabolic process |
| FN998658 | FN998658 FN998658 *Olea europaea* flower *Olea europaea* cDNA clone c2-6-A1 | 2.61 | 0.02 | Carbohydrate metabolic process |
| FN998761 | FN998761 FN998761 *Olea europaea* flower *Olea europaea* cDNA clone c2-7-B10 | 2.62 | 0.01 | Carbohydrate metabolic process |
| FL683658 | FL683658 A_C08_B04_0414F_p2 *Olea europaea* cv. Leccino fruitlet *Olea europaea* cDNA | 2.63 | 0.02 | Unknown |
| FN998328 | FN998328 FN998328 *Olea europaea* flower *Olea europaea* cDNA clone c2-5-H5 | 2.63 | 0.01 | Carbohydrate metabolic process |
| FN998398 | FN998398 FN998398 *Olea europaea* flower *Olea europaea* cDNA clone c2-2-A7 | 2.64 | 0.02 | Carbohydrate metabolic process |
| GO243399 | GO243399 OEAA-070810_Plate1n17.b1 cDNA library from Olive leaves and fruits *Olea europaea* cDNA | 2.64 | 0 | Biosynthetic process |
| FN998438 | FN998438 FN998438 *Olea europaea* flower *Olea europaea* cDNA clone c2-2-E6 | 2.66 | 0.02 | Carbohydrate metabolic process |
| FN998394 | FN998394 FN998394 *Olea europaea* flower *Olea europaea* cDNA clone c2-2-A3 | 2.66 | 0.02 | Carbohydrate metabolic process |
| GO245337 | GO245337 OEAA-070810_Plate7b02.b1 cDNA library from Olive leaves and fruits *Olea europaea* cDNA | 2.66 | 0.03 | Oxidation-reduction process |
| FN998742 | FN998742 FN998742 *Olea europaea* flower *Olea europaea* cDNA clone c2-6-H3 | 2.67 | 0.01 | Carbohydrate metabolic process |
| FN998386 | FN998386 FN998386 *Olea europaea* flower *Olea europaea* cDNA clone c2-1-H5 | 2.67 | 0.02 | Carbohydrate metabolic process |
| GO242845 | GO242845 OEAA-070810_Plate10f24.b1 cDNA library from Olive leaves and fruits *Olea europaea* cDNA | 2.67 | 0.02 | Unknown |
| FN998350 | FN998350 FN998350 *Olea europaea* flower *Olea europaea* cDNA clone c2-1-D7 | 2.67 | 0 | Carbohydrate metabolic process |
| FN998361 | FN998361 FN998361 *Olea europaea* flower *Olea europaea* cDNA clone c2-1-E9 | 2.68 | 0.03 | Carbohydrate metabolic process |
| FN998578 | FN998578 FN998578 *Olea europaea* flower *Olea europaea* cDNA clone c2-4-B2 | 2.68 | 0.03 | Carbohydrate metabolic process |
| FL684071 | FL684071 A_O23_H12_0414C_p1 *Olea europaea* cv. Leccino fruitlet *Olea europaea* cDNA | 2.69 | 0.02 | Unknown |
| FN998775 | FN998775 FN998775 *Olea europaea* flower *Olea europaea* cDNA clone c2-7-C3 | 2.69 | 0.01 | Carbohydrate metabolic process |
| FN998036 | FN998036 FN998036 *Olea europaea* flower *Olea europaea* cDNA clone c1-5-E1 | 2.69 | 0.03 | ATP hydrolysis coupled proton transport, Golgi organization, defense response to bacterium, plant-type cell wall biogenesis, response to cold, response to salt stress |
| gw1.VI.2898.1 | protease inhibitor/seed storage/lipid transfer protein (LTP) family protein | 2.71 | 0.01 | Lipid transport |
| FN998462 | FN998462 FN998462 *Olea europaea* flower *Olea europaea* cDNA clone c2-2-H11 | 2.71 | 0.01 | Carbohydrate metabolic process |
| FN998326 | FN998326 FN998326 *Olea europaea* flower *Olea europaea* cDNA clone c2-5-H3 | 2.71 | 0.02 | Carbohydrate metabolic process |
| GO245328 | GO245328 OEAA-070810_Plate7a17.b1 cDNA library from Olive leaves and fruits *Olea europaea* cDNA | 2.71 | 0 | Defense response by callose deposition, response to chitin, response to mechanical stimulus, response to wounding, salicylic acid biosynthetic process, systemic acquired resistance |
| FN998419 | FN998419 FN998419 *Olea europaea* flower *Olea europaea* cDNA clone c2-2-C6 | 2.72 | 0.01 | Carbohydrate metabolic process |
| FN998416 | FN998416 FN998416 *Olea europaea* flower *Olea europaea* cDNA clone c2-2-C3 | 2.72 | 0.01 | Carbohydrate metabolic process |
| FN998617 | FN998617 FN998617 *Olea europaea* flower *Olea europaea* cDNA clone c2-4-E5 | 2.72 | 0.02 | Carbohydrate metabolic process |
| FN997779 | FN997779 FN997779 *Olea europaea* flower *Olea europaea* cDNA clone c1-2-E11 | 2.74 | 0.04 | Unknown |
| FN998711 | FN998711 FN998711 *Olea europaea* flower *Olea europaea* cDNA clone c2-6-E7 | 2.74 | 0.01 | Carbohydrate metabolic process |
| FN998365 | FN998365 FN998365 *Olea europaea* flower *Olea europaea* cDNA clone c2-1-F12 | 2.75 | 0.02 | Carbohydrate metabolic process |
| GO243981 | GO243981 OEAA-070810_Plate3g21.b1 cDNA library from Olive leaves and fruits *Olea europaea* cDNA | 2.75 | 0.04 | Unknown |
| FN998640 | FN998640 FN998640 *Olea europaea* flower *Olea europaea* cDNA clone c2-4-G4 | 2.76 | 0.03 | Carbohydrate metabolic process |
| FN998281 | FN998281 FN998281 *Olea europaea* flower *Olea europaea* cDNA clone c2-5-B7 | 2.77 | 0.01 | Carbohydrate metabolic process |
| eugene3.01650006 | pollen Ole e 1 allergen and extensin family protein | 2.77 | 0 | Unknown |
| fgenesh4_pg.C_scaffold_17737000001 | serine/threonine protein kinase family protein | 2.78 | 0.03 | Protein phosphorylation, response to bacterium |
| FN998235 | FN998235 FN998235 *Olea europaea* flower *Olea europaea* cDNA clone c1-7-F3 | 2.78 | 0.02 | ATP hydrolysis coupled proton transport, defense response to bacterium, Golgi organization, plant-type cell wall biogenesis, response to cold, response to salt stress |
| FN998834 | FN998834 FN998834 *Olea europaea* flower *Olea europaea* cDNA clone c2-7-H5 | 2.8 | 0.01 | Carbohydrate metabolic process |
| FN998274 | FN998274 FN998274 *Olea europaea* flower *Olea europaea* cDNA clone c2-5-B10 | 2.8 | 0.02 | Carbohydrate metabolic process |
| FN998315 | FN998315 FN998315 *Olea europaea* flower *Olea europaea* cDNA clone c2-5-F3 | 2.82 | 0 | Unknown |
| GO243101 | GO243101 OEAA-070810_Plate1a19.b1 cDNA library from Olive leaves and fruits *Olea europaea* cDNA | 2.83 | 0.01 | Microtubule-based movement, regulation of defense response, starch metabolic process |
| FN998485 | FN998485 FN998485 *Olea europaea* flower *Olea europaea* cDNA clone c2-3-B2 | 2.85 | 0.01 | Carbohydrate metabolic process |
| FN998362 | FN998362 FN998362 *Olea europaea* flower *Olea europaea* cDNA clone c2-1-F1 | 2.85 | 0.02 | Carbohydrate metabolic process |
| FN998717 | FN998717 FN998717 *Olea europaea* flower *Olea europaea* cDNA clone c2-6-F12 | 2.85 | 0.02 | Carbohydrate metabolic process |
| FN998529 | FN998529 FN998529 *Olea europaea* flower *Olea europaea* cDNA clone c2-3-F11 | 2.85 | 0 | Carbohydrate metabolic process |
| FN998452 | FN998452 FN998452 *Olea europaea* flower *Olea europaea* cDNA clone c2-2-F9 | 2.86 | 0.04 | Carbohydrate metabolic process |
| FN998576 | FN998576 FN998576 *Olea europaea* flower *Olea europaea* cDNA clone c2-4-B11 | 2.86 | 0.01 | Carbohydrate metabolic process |
| eugene3.00140920 | TT4, ATCHS, CHS \| CHS (CHALCONE SYNTHASE) | 2.89 | 0.02 | Response to wounding, response to jasmonic acid response, response to UV-B, response to gravity, flavonoid biosynthetic process, response to oxidative stress, response to auxin stimulus |
| estExt_fgenesh4_pg.C_LG_II2533 | tyrosine decarboxylase, putative | 2.89 | 0.01 | Carboxylic acid metabolic process, response to wounding, amino acid metabolic process |
| gw1.XI.1469.1 | similar to unnamed protein product [Ostreococcus tauri] (GB:CAL56420.1) | 2.91 | 0.02 | Protein folding, response to water deprivation, response to salt stress, response to cold |
| FN998298 | FN998298 FN998298 *Olea europaea* flower *Olea europaea* cDNA clone c2-5-D5 | 2.92 | 0.03 | Carbohydrate metabolic process |
| GO244795 | GO244795 OEAA-070810_Plate5j18.b1 cDNA library from Olive leaves and fruits *Olea europaea* cDNA | 2.92 | 0 | Proteolysis |
| FL683503 | FL683503 B_E13_C07_0414F_p5 *Olea europaea* cv. Leccino fruitlet *Olea europaea* cDNA | 2.94 | 0 | Unknown |
| FN998355 | FN998355 FN998355 *Olea europaea* flower *Olea europaea* cDNA clone c2-1-E2 | 2.94 | 0.02 | Carbohydrate metabolic process |
| FN998436 | FN998436 FN998436 *Olea europaea* flower *Olea europaea* cDNA clone c2-2-E4 | 2.99 | 0 | Carbohydrate metabolic process |
| FN998647 | FN998647 FN998647 *Olea europaea* flower *Olea europaea* cDNA clone c2-4-H10 | 3.01 | 0.02 | Carbohydrate metabolic process |
| FN998344 | FN998344 FN998344 *Olea europaea* flower *Olea europaea* cDNA clone c2-1-D12 | 3.01 | 0.03 | Carbohydrate metabolic process |
| FN998488 | FN998488 FN998488 *Olea europaea* flower *Olea europaea* cDNA clone c2-3-B5 | 3.02 | 0.02 | Carbohydrate metabolic process |
| FN998740 | FN998740 FN998740 *Olea europaea* flower *Olea europaea* cDNA clone c2-6-H12 | 3.03 | 0.02 | Carbohydrate metabolic process |
| GO244021 | GO244021 OEAA-070810_Plate3i13.b1 cDNA library from Olive leaves and fruits *Olea europaea* cDNA | 3.06 | 0 | Unknown |
| FN998814 | FN998814 FN998814 *Olea europaea* flower *Olea europaea* cDNA clone c2-7-F8 | 3.06 | 0.02 | Carbohydrate metabolic process |
| FN998655 | FN998655 FN998655 *Olea europaea* flower *Olea europaea* cDNA clone c2-4-H7 | 3.06 | 0.02 | Carbohydrate metabolic process |
| GO245187 | GO245187 OEAA-070810_Plate6k13.b1 cDNA library from Olive leaves and fruits *Olea europaea* cDNA | 3.07 | 0.02 | Unknown |
| FN998746 | FN998746 FN998746 *Olea europaea* flower *Olea europaea* cDNA clone c2-6-H7 | 3.07 | 0.02 | Carbohydrate metabolic process |
| FN998786 | FN998786 FN998786 *Olea europaea* flower *Olea europaea* cDNA clone c2-7-D3 | 3.11 | 0.02 | Carbohydrate metabolic process |
| gw1.558.6.1 | integrase [Populus trichocarpa] | 3.11 | 0.02 | Unknown |
| FN998641 | FN998641 FN998641 *Olea europaea* flower *Olea europaea* cDNA clone c2-4-G5 | 3.13 | 0.02 | Carbohydrate metabolic process |
| FN998262 | FN998262 FN998262 *Olea europaea* flower *Olea europaea* cDNA clone c2-5-A1 | 3.14 | 0.02 | Carbohydrate metabolic process |
| FN998310 | FN998310 FN998310 *Olea europaea* flower *Olea europaea* cDNA clone c2-5-E8 | 3.15 | 0.02 | Carbohydrate metabolic process |
| FN998748 | FN998748 FN998748 *Olea europaea* flower *Olea europaea* cDNA clone c2-6-H9 | 3.16 | 0.02 | Carbohydrate metabolic process |
| FN997851 | FN997851 FN997851 *Olea europaea* flower *Olea europaea* cDNA clone c1-3-D11 | 3.16 | 0.02 | Carbohydrate metabolic process |
| FN998643 | FN998643 FN998643 *Olea europaea* flower *Olea europaea* cDNA clone c2-4-G7 | 3.16 | 0.02 | Carbohydrate metabolic process |
| FN998454 | FN998454 FN998454 *Olea europaea* flower *Olea europaea* cDNA clone c2-2-G2 | 3.16 | 0.03 | Carbohydrate metabolic process |
| FN998387 | FN998387 FN998387 *Olea europaea* flower *Olea europaea* cDNA clone c2-1-H6 | 3.16 | 0.01 | Carbohydrate metabolic process |
| FN998591 | FN998591 FN998591 *Olea europaea* flower *Olea europaea* cDNA clone c2-4-C3 | 3.22 | 0.04 | Carbohydrate metabolic process |
| FN998276 | FN998276 FN998276 *Olea europaea* flower *Olea europaea* cDNA clone c2-5-B2 | 3.22 | 0.02 | Carbohydrate metabolic process |
| FN998573 | FN998573 FN998573 *Olea europaea* flower *Olea europaea* cDNA clone c2-4-A9 | 3.22 | 0.04 | Unknown |
| FN998545 | FN998545 FN998545 *Olea europaea* flower *Olea europaea* cDNA clone c2-3-G4 | 3.25 | 0.02 | Carbohydrate metabolic process |
| FN998470 | FN998470 FN998470 *Olea europaea* flower *Olea europaea* cDNA clone c2-3-A1 | 3.25 | 0.01 | Carbohydrate metabolic process |
| FN998353 | FN998353 FN998353 *Olea europaea* flower *Olea europaea* cDNA clone c2-1-E10 | 3.26 | 0.02 | Carbohydrate metabolic process |
| FN998701 | FN998701 FN998701 *Olea europaea* flower *Olea europaea* cDNA clone c2-6-D9 | 3.29 | 0.04 | Carbohydrate metabolic process |
| FN998323 | FN998323 FN998323 *Olea europaea* flower *Olea europaea* cDNA clone c2-5-H11 | 3.3 | 0.03 | Carbohydrate metabolic process |
| FN998789 | FN998789 FN998789 *Olea europaea* flower *Olea europaea* cDNA clone c2-7-D6 | 3.3 | 0.01 | Carbohydrate metabolic process |
| FN998441 | FN998441 FN998441 *Olea europaea* flower *Olea europaea* cDNA clone c2-2-E9 | 3.34 | 0.03 | Carbohydrate metabolic process |
| GO243710 | GO243710 OEAA-070810_Plate2l05.b1 cDNA library from Olive leaves and fruits *Olea europaea* cDNA | 3.34 | 0.05 | Metabolic process |
| FN998472 | FN998472 FN998472 *Olea europaea* flower *Olea europaea* cDNA clone c2-3-A11 | 3.4 | 0.01 | Carbohydrate metabolic process |
| FN998415 | FN998415 FN998415 *Olea europaea* flower *Olea europaea* cDNA clone c2-2-C2 | 3.41 | 0.04 | Unknown |
| FN997873 | FN997873 FN997873 *Olea europaea* flower *Olea europaea* cDNA clone c1-3-F10 | 3.42 | 0.02 | Carbohydrate metabolic process |
| GO244789 | GO244789 OEAA-070810_Plate5j12.b1 cDNA library from Olive leaves and fruits *Olea europaea* cDNA | 3.45 | 0.02 | Unknown |
| FN997782 | FN997782 FN997782 *Olea europaea* flower *Olea europaea* cDNA clone c1-2-E3 | 3.59 | 0.01 | Response to karrikin |
| FN997886 | FN997886 FN997886 *Olea europaea* flower *Olea europaea* cDNA clone c1-3-G5 | 3.6 | 0 | Carbohydrate metabolic process |
| FN998631 | FN998631 FN998631 *Olea europaea* flower *Olea europaea* cDNA clone c2-4-F7 | 3.62 | 0 | Carbohydrate metabolic process |
| FN998502 | FN998502 FN998502 *Olea europaea* flower *Olea europaea* cDNA clone c2-3-C8 | 3.63 | 0 | Carbohydrate metabolic process |
| FN998669 | FN998669 FN998669 *Olea europaea* flower *Olea europaea* cDNA clone c2-6-B1 | 3.65 | 0.02 | Carbohydrate metabolic process |
| GO245644 | GO245644 OEAA-070810_Plate7o04.b1 cDNA library from Olive leaves and fruits *Olea europaea* cDNA | 3.68 | 0.01 | Alkaloid metabolic process, systemic acquired resistance, salicylic acid mediated signaling pathway |
| FN998369 | FN998369 FN998369 *Olea europaea* flower *Olea europaea* cDNA clone c2-1-F8 | 3.69 | 0 | Unknown |
| FN998538 | FN998538 FN998538 *Olea europaea* flower *Olea europaea* cDNA clone c2-3-F9 | 3.7 | 0.03 | Carbohydrate metabolic process |
| FL684059 | FL684059 D_J04_E02_0414F_p12 *Olea europaea* cv. Leccino fruitlet *Olea europaea* cDNA | 3.73 | 0.01 | Oxidation-reduction process |
| GO245800 | GO245800 OEAA-070810_Plate8e19.b1 cDNA library from Olive leaves and fruits *Olea europaea* cDNA | 3.86 | 0.01 | Calcium ion transport, Golgi organization, phenylpropanoid metabolic process, polyamine catabolic process, response to cadmium ion, response to salt stress, S-adenosylmethionine biosynthetic process |
| GO245535 | GO245535 OEAA-070810_Plate7j13.b1 cDNA library from Olive leaves and fruits *Olea europaea* cDNA | 4.36 | 0.01 | Response to karrikin |
| GO243695 | GO243695 OEAA-070810_Plate2k11.b1 cDNA library from Olive leaves and fruits *Olea europaea* cDNA | 4.39 | 0.02 | Unknown |
| GO245017 | GO245017 OEAA-070810_Plate6d08.b1 cDNA library from Olive leaves and fruits *Olea europaea* cDNA | 4.51 | 0.01 | Lipid transport |
| FL684126 | FL684126 D_B23_A12_0414F_p11 *Olea europaea* cv. Leccino fruitlet *Olea europaea* cDNA | 4.66 | 0.01 | Flavonoid biosynthetic process |
| GO243651 | GO243651 OEAA-070810_Plate2i13.b1 cDNA library from Olive leaves and fruits *Olea europaea* cDNA | 4.72 | 0.01 | Proteolysis |
| GO245913 | GO245913 OEAA-070810_Plate8j14.b1 cDNA library from Olive leaves and fruits *Olea europaea* cDNA | 4.92 | 0 | Carbohydrate metabolic process, regulation of meristem growth |
| FL683787 | FL683787 D_E21_C11_0414F_p13 *Olea europaea* cv. Leccino fruitlet *Olea europaea* cDNA | 5.42 | 0.02 | Unknown |
