# Supplementary material for: Nutrition Metabolism Plays an Important Role in the Alternate Bearing of the Olive Tree (Olea europaea L.)
Source: PLoS One. 2013 Mar 28;8(3):e59876. doi: 10.1371/journal.pone.0059876 (PMC3610735; doi:10.1371/journal.pone.0059876)
Supplement: Table S3 — Differentially expressed transcripts between mature and juvenile leaves, irrespective to the bearing year. Fold changes were given in log2-based numbers. (−) indicates down-regulation. (DOCX) [file pone.0059876.s003.docx]

**Table S2.** Differentially expressed transcripts between mature and juvenile leaves, irrespective to the bearing year. Fold changes were given in log2-based numbers. (-) indicates down-regulation.

| **Seq_ID** | **Description** | **ON-M /ON-J-fold change** | ***P* value** | **OFF-M /OFF-J-fold change** | ***P* value** | **GO biological process** |
| --- | --- | --- | --- | --- | --- | --- |
| GO243991 | GO243991 OEAA-070810_Plate3h07.b1 cDNA library from Olive leaves and fruits *Olea europaea* cDNA | –6.21 | 0 | –2.31 | 0.01 | Fatty acid metabolic process, oxidation-reduction process |
| GO243211 | GO243211 OEAA-070810_Plate1f15.b1 cDNA library from Olive leaves and fruits *Olea europaea* cDNA | –5.85 | 0 | –2.08 | 0 | Response to biotic stimulus |
| GO243814 | GO243814 OEAA-070810_Plate2p19.b1 cDNA library from Olive leaves and fruits *Olea europaea* cDNA | –5.84 | 0 | –2.08 | 0 | Defense response, response to biotic stimulus |
| GO244140 | GO244140 OEAA-070810_Plate3n12.b1 cDNA library from Olive leaves and fruits *Olea europaea* cDNA | –5.55 | 0 | –2.62 | 0.01 | Fatty acid metabolic process, oxidation-reduction process |
| GO244529 | GO244529 OEAA-070810_Plate4o02.b1 cDNA library from Olive leaves and fruits *Olea europaea* cDNA | –5.51 | 0 | –2.79 | 0 | Unknown |
| GO245304 | GO245304 OEAA-070810_Plate6p15.b1 cDNA library from Olive leaves and fruits *Olea europaea* cDNA | –5.35 | 0 | –3.01 | 0 | Response to karrikin, syncytium formation |
| GO245145 | GO245145 OEAA-070810_Plate6i18.b1 cDNA library from Olive leaves and fruits *Olea europaea* cDNA | –5.29 | 0 | –2.46 | 0 | Cell wall biogenesis, trichome morphogenesis |
| GO244729 | GO244729 OEAA-070810_Plate5g22.b1 cDNA library from Olive leaves and fruits *Olea europaea* cDNA | –5.01 | 0 | –2.48 | 0 | Cell wall biogenesis, cysteine biosynthetic process, trichome morphogenesis |
| GO245518 | GO245518 OEAA-070810_Plate7i20.b1 cDNA library from Olive leaves and fruits *Olea europaea* cDNA | –4.83 | 0 | –2.63 | 0 | Regulation of secondary cell wall biogenesis, regulation of transcription, DNA-dependent, response to abscisic acid stimulus |
| GO245492 | GO245492 OEAA-070810_Plate7h17.b1 cDNA library from Olive leaves and fruits *Olea europaea* cDNA | –4.72 | 0 | –2.26 | 0 | Unknown |
| GO243777 | GO243777 OEAA-070810_Plate2o06.b1 cDNA library from Olive leaves and fruits *Olea europaea* cDNA | –4.68 | 0 | –2.67 | 0 | Type I hypersensitivity |
| GO243932 | GO243932 OEAA-070810_Plate3e19.b1 cDNA library from Olive leaves and fruits *Olea europaea* cDNA | –4.66 | 0 | –2.44 | 0.01 | Unknown |
| GO245517 | GO245517 OEAA-070810_Plate7i19.b1 cDNA library from Olive leaves and fruits *Olea europaea* cDNA | –4.49 | 0 | –2.55 | 0 | Regulation of secondary cell wall biogenesis, regulation of transcription, DNA-dependent, response to abscisic acid stimulus |
| GO243710 | GO243710 OEAA-070810_Plate2l05.b1 cDNA library from Olive leaves and fruits *Olea europaea* cDNA | –3.91 | 0 | 3.34 | 0.05 | Metabolic process |
| FN998508 | FN998508 FN998508 *Olea europaea* flower *Olea europaea* cDNA clone c2-3-D2 | –3.88 | 0 | –2.08 | 0 | Mucilage extrusion from seed coat, mucilage metabolic process involved seed coat development, negative regulation of catalytic activity, proteolysis |
| GO244599 | GO244599 OEAA-070810_Plate5b03.b1 cDNA library from Olive leaves and fruits *Olea europaea* cDNA | –3.61 | 0 | –2 | 0 | Urea transmembrane transport, water transport |
| FL684184 | FL684184 C_D12_B06_0414F_p8 *Olea europaea* cv. Leccino fruitlet *Olea europaea* cDNA | –3.47 | 0 | –2.65 | 0 | Heat acclimation, protein folding, protein unfolding, response to endoplasmic reticulum stress, response to high light intensity, response to hydrogen peroxide |
| FL683438 | FL683438 D_K16_F08_0414F_p14 *Olea europaea* cv. Leccino fruitlet *Olea europaea* cDNA | –3.43 | 0 | –2.64 | 0 | Unknown |
| GO246183 | GO246183 OEAA-070810_Plate9f01.b1 cDNA library from Olive leaves and fruits *Olea europaea* cDNA | –3.2 | 0.03 | –2.06 | 0.01 | Unknown |
| GO244287 | GO244287 OEAA-070810_Plate4d17.b1 cDNA library from Olive leaves and fruits *Olea europaea* cDNA | –2.72 | 0 | 2.05 | 0.02 | Unknown |
| eugene3.00101184 | similar to ECERIFERUM 6; similar to ECERIFERUM 60; similar to CUTICULAR 1; similar to POLLEN-PISTIL INCOMPATIBILITY 1 | –2.71 | 0.02 | –2.31 | 0.01 | Metabolic process, fatty acid biosynthetic process, lipid biosynthetic process |
| FL684126 | FL684126 D_B23_A12_0414F_p11 *Olea europaea* cv. Leccino fruitlet *Olea europaea* cDNA | –2.24 | 0 | 4.66 | 0.01 | Flavonoid biosynthetic process |
| GO243399 | GO243399 OEAA-070810_Plate1n17.b1 cDNA library from Olive leaves and fruits *Olea europaea* cDNA | –2.15 | 0.01 | 2.64 | 0 | Biosynthetic process |
| GO244795 | GO244795 OEAA-070810_Plate5j18.b1 cDNA library from Olive leaves and fruits *Olea europaea* cDNA | 2.11 | 0.01 | 2.92 | 0 | Proteolysis |
| eugene3.01650006 | pollen Ole e 1 allergen and extensin family protein | 2.22 | 0.01 | 2.77 | 0 | Circadian rhythm |
| FN998573 | FN998573 FN998573 *Olea europaea* flower *Olea europaea* cDNA clone c2-4-A9 | 2.25 | 0 | 3.22 | 0.04 | Unknown |
| FL683503 | FL683503 B_E13_C07_0414F_p5 *Olea europaea* cv. Leccino fruitlet *Olea europaea* cDNA | 2.31 | 0 | 2.94 | 0 | Unknown |
| GO243458 | GO243458 OEAA-070810_Plate2a05.b1 cDNA library from Olive leaves and fruits *Olea europaea* cDNA | 2.43 | 0.01 | 2.47 | 0 | Multidimensional cell growth, regulation of transcription, DNA-dependent |
| FN997782 | FN997782 FN997782 *Olea europaea* flower *Olea europaea* cDNA clone c1-2-E3 | 2.53 | 0.01 | 3.59 | 0.01 | Response to karrikin |
| GO243444 | GO243444 OEAA-070810_Plate1p14.b1 cDNA library from Olive leaves and fruits *Olea europaea* cDNA | 2.58 | 0 | 2.57 | 0 | Unknown |
| FL684059 | FL684059 D_J04_E02_0414F_p12 *Olea europaea* cv. Leccino fruitlet *Olea europaea* cDNA | 2.59 | 0 | 3.73 | 0.01 | Oxidation-reduction process |
| GO245913 | GO245913 OEAA-070810_Plate8j14.b1 cDNA library from Olive leaves and fruits *Olea europaea* cDNA | 2.82 | 0 | 4.92 | 0 | Carbohydrate metabolic process, regulation of meristem growth |
| GO244789 | GO244789 OEAA-070810_Plate5j12.b1 cDNA library from Olive leaves and fruits *Olea europaea* cDNA | 2.84 | 0 | 3.45 | 0.02 | Unknown |
| GO243651 | GO243651 OEAA-070810_Plate2i13.b1 cDNA library from Olive leaves and fruits *Olea europaea* cDNA | 2.92 | 0 | 4.72 | 0.01 | Proteolysis |
| estExt_fgenesh4_pg.C_LG_II2533 | tyrosine decarboxylase | 3.19 | 0 | 2.89 | 0.01 | Tyrosine decarboxylase activity |
| GO245535 | GO245535 OEAA-070810_Plate7j13.b1 cDNA library from Olive leaves and fruits *Olea europaea* cDNA | 3.79 | 0 | 4.36 | 0.01 | Response to karrikin |
