# Supplementary material for: Nutrition Metabolism Plays an Important Role in the Alternate Bearing of the Olive Tree (Olea europaea L.)
Source: PLoS One. 2013 Mar 28;8(3):e59876. doi: 10.1371/journal.pone.0059876 (PMC3610735; doi:10.1371/journal.pone.0059876)
Supplement: Table S4 — Differentially expressed transcripts between the “on” and “off” years. Fold changes were given in log2-based numbers. (−) indicates down-regulation. (DOCX) [file pone.0059876.s004.docx]

**Table S3.** Differentially expressed transcripts between the “on” and “off” years. Fold changes were given in log2-based numbers. (-) indicates down-regulation.

| **Seq_ID** | **Description** | **ON-M /OFF-M-fold change** | ***P* value** | **GO biological process** |
| --- | --- | --- | --- | --- |
| FL684392_1 | FL684392 A_K15_F08_0414F_p1 *Olea europaea* cv. Leccino fruitlet *Olea europaea* cDNA | –6.42 | 0.00 | Unknown |
| FL683857_1 | FL683857 A_C23_B12_0414C_p1 *Olea europaea* cv. Leccino fruitlet *Olea europaea* cDNA | –6.15 | 0.00 | Cellular amino acid metabolic process |
| GO245805_1 | GO245805 OEAA-070810_Plate8e24.b1 cDNA library from Olive leaves and fruits *Olea europaea* cDNA | –6.03 | 0.00 | Cellular amino acid metabolic process |
| FL683748_1 | FL683748 A_M22_G11_0414F_p2 *Olea europaea* cv. Leccino fruitlet *Olea europaea* cDNA | –5.91 | 0.00 | Cellular amino acid metabolic process |
| FL683833_1 | FL683833 A_K01_F01_0414C_p1 *Olea europaea* cv. Leccino fruitlet *Olea europaea* cDNA | –5.85 | 0.00 | Cellular amino acid metabolic process |
| FL683787_1 | FL683787 D_E21_C11_0414F_p13 *Olea europaea* cv. Leccino fruitlet *Olea europaea* cDNA | –5.41 | 0.00 | Unknown |
| GO243808_1 | GO243808 OEAA-070810_Plate2p13.b1 cDNA library from Olive leaves and fruits *Olea europaea* cDNA | –5.23 | 0.00 | Cellular amino acid metabolic process |
| FL684126_1 | FL684126 D_B23_A12_0414F_p11 *Olea europaea* cv. Leccino fruitlet *Olea europaea* cDNA | –4.76 | 0.00 | Flavonoid biosynthetic process |
| FL684185_1 | FL684185 B_N02_G01_0414F_p4 *Olea europaea* cv. Leccino fruitlet *Olea europaea* cDNA | –4.58 | 0.00 | Nucleoside metabolic process, protein ubiquitination |
| GO243710_1 | GO243710 OEAA-070810_Plate2l05.b1 cDNA library from Olive leaves and fruits *Olea europaea* cDNA | –4.48 | 0.00 | Metabolic process |
| GO245050_1 | GO245050 OEAA-070810_Plate6e17.b1 cDNA library from Olive leaves and fruits *Olea europaea* cDNA | –4.36 | 0.00 | Cellular amino acid metabolic process |
| GO245017_1 | GO245017 OEAA-070810_Plate6d08.b1 cDNA library from Olive leaves and fruits *Olea europaea* cDNA | –4.21 | 0.00 | Lipid transport |
| FL683725_1 | FL683725 A_I08_E04_0414F_p2 *Olea europaea* cv. Leccino fruitlet *Olea europaea* cDNA | –4.19 | 0.00 | Chlorophyll biosynthetic process, terpenoid biosynthetic process |
| GO243394_1 | GO243394 OEAA-070810_Plate1n12.b1 cDNA library from Olive leaves and fruits *Olea europaea* cDNA | –4.13 | 0.00 | Unknown |
| FL683395_1 | FL683395 C_O09_H05_0414F_p9 *Olea europaea* cv. Leccino fruitlet *Olea europaea* cDNA | –4.04 | 0.00 | Unknown |
| GO245613_1 | GO245613 OEAA-070810_Plate7m21.b1 cDNA library from Olive leaves and fruits *Olea europaea* cDNA | –3.86 | 0.00 | Metabolic process |
| GO243938_1 | GO243938 OEAA-070810_Plate3f01.b1 cDNA library from Olive leaves and fruits *Olea europaea* cDNA | –3.85 | 0.00 | Unknown |
| GO244502_1 | GO244502 OEAA-070810_Plate4m21.b1 cDNA library from Olive leaves and fruits *Olea europaea* cDNA | –3.81 | 0.00 | Cellular amino acid metabolic process |
| GO243399_1 | GO243399 OEAA-070810_Plate1n17.b1 cDNA library from Olive leaves and fruits *Olea europaea* cDNA | –3.81 | 0.00 | Biosynthetic process |
| GO243240_1 | GO243240 OEAA-070810_Plate1g20.b1 cDNA library from Olive leaves and fruits *Olea europaea* cDNA | –3.78 | 0.00 | Lipid metabolic process |
| GO245023_1 | GO245023 OEAA-070810_Plate6d14.b1 cDNA library from Olive leaves and fruits *Olea europaea* cDNA | –3.69 | 0.00 | Cuticle development, drug transmembrane transport, ATP catabolic process |
| GO244287_1 | GO244287 OEAA-070810_Plate4d17.b1 cDNA library from Olive leaves and fruits *Olea europaea* cDNA | –3.68 | 0.00 | Unknown |
| GO243712_1 | GO243712 OEAA-070810_Plate2l07.b1 cDNA library from Olive leaves and fruits *Olea europaea* cDNA | –3.60 | 0.00 | Plant-type cell wall organization |
| GO243695_1 | GO243695 OEAA-070810_Plate2k11.b1 cDNA library from Olive leaves and fruits *Olea europaea* cDNA | –3.59 | 0.00 | Unknown |
| FL683544_1 | FL683544 B_G18_D09_0414C_p2 *Olea europaea* cv. Leccino fruitlet *Olea europaea* cDNA | –3.49 | 0.00 | Unknown |
| GO244165_1 | GO244165 OEAA-070810_Plate3o13.b1 cDNA library from Olive leaves and fruits *Olea europaea* cDNA | –3.43 | 0.00 | Response to abscisic acid stimulus, transmembrane transport, water transport |
| grail3.0111003801 | calmodulin-binding family protein | –3.43 | 0.00 | Unknown |
| GO243211_1 | GO243211 OEAA-070810_Plate1f15.b1 cDNA library from Olive leaves and fruits *Olea europaea* cDNA | –3.41 | 0.00 | Defense response, response to biotic stimulus |
| GO243814_1 | GO243814 OEAA-070810_Plate2p19.b1 cDNA library from Olive leaves and fruits *Olea europaea* cDNA | –3.37 | 0.00 | Defense response, response to biotic stimulus, response to biotic stimulus |
| GO243991_1 | GO243991 OEAA-070810_Plate3h07.b1 cDNA library from Olive leaves and fruits *Olea europaea* cDNA | –3.35 | 0.00 | Fatty acid metabolic process, oxidation-reduction process |
| GO243114_1 | GO243114 OEAA-070810_Plate1b09.b1 cDNA library from Olive leaves and fruits *Olea europaea* cDNA | –3.31 | 0.00 | Flavonol biosynthetic process, oxidation-reduction process, response to karrikin, response to light stimulus |
| GO244094_1 | GO244094 OEAA-070810_Plate3l14.b1 cDNA library from Olive leaves and fruits *Olea europaea* cDNA | –3.24 | 0.00 | Fatty acid metabolic process, oxidation-reduction process |
| GO244351_1 | GO244351 OEAA-070810_Plate4g09.b1 cDNA library from Olive leaves and fruits *Olea europaea* cDNA | –3.21 | 0.00 | Metabolic process |
| GO245020_1 | GO245020 OEAA-070810_Plate6d11.b1 cDNA library from Olive leaves and fruits *Olea europaea* cDNA | –3.14 | 0.00 | Unknown |
| GO246421_1 | GO246421 OEAA-070810_Plate9p09.b1 cDNA library from Olive leaves and fruits *Olea europaea* cDNA | –3.09 | 0.00 | Defense response to fungus, incompatible interaction, salicylic acid metabolic process, systemic acquired resistance, salicylic acid mediated signalling pathway |
| GO244677_1 | GO244677 OEAA-070810_Plate5e13.b1 cDNA library from Olive leaves and fruits *Olea europaea* cDNA | –3.03 | 0.00 | Response to wounding, steroid metabolic process, xylem and phloem pattern formation |
| GO246070_1 | GO246070 OEAA-070810_Plate9a08.b1 cDNA library from Olive leaves and fruits *Olea europaea* cDNA | –3.01 | 0.00 | Unknown |
| GO244999_1 | GO244999 OEAA-070810_Plate6c13.b1 cDNA library from Olive leaves and fruits *Olea europaea* cDNA | –2.99 | 0.00 | Unknown |
| GO244836_1 | GO244836 OEAA-070810_Plate5l12.b1 cDNA library from Olive leaves and fruits *Olea europaea* cDNA | –2.99 | 0.00 | Unknown |
| GO243842_1 | GO243842 OEAA-070810_Plate3b01.b1 cDNA library from Olive leaves and fruits *Olea europaea* cDNA | –2.97 | 0.00 | Oxidation-reduction process, amine metabolic process |
| GO243358_1 | GO243358 OEAA-070810_Plate1l24.b1 cDNA library from Olive leaves and fruits *Olea europaea* cDNA | –2.96 | 0.00 | Oxidation-reduction process, amine metabolic process, amine metabolic process |
| GO243848_1 | GO243848 OEAA-070810_Plate3b07.b1 cDNA library from Olive leaves and fruits *Olea europaea* cDNA | –2.95 | 0.00 | Type I hypersensitivity |
| GO243043_1 | GO243043 OEAA-070810_Plate10o07.b1 cDNA library from Olive leaves and fruits *Olea europaea* cDNA | –2.95 | 0.00 | Cellular divalent inorganic cation homeostasis, monovalent inorganic cation transport, response to abiotic stimulus, response to chemical stimulus, response to stress, transmembrane transport, calcium ion transport |
| GO243363_1 | GO243363 OEAA-070810_Plate1m05.b1 cDNA library from Olive leaves and fruits *Olea europaea* cDNA | –2.93 | 0.00 | Fatty acid metabolic process, oxidation-reduction process, oxidation-reduction process |
| GO243553_1 | GO243553 OEAA-070810_Plate2e07.b1 cDNA library from Olive leaves and fruits *Olea europaea* cDNA | –2.93 | 0.00 | Response to karrikin, response to sucrose stimulus, response to UV-B, anthocyanin biosynthetic process |
| GO243402_1 | GO243402 OEAA-070810_Plate1n20.b1 cDNA library from Olive leaves and fruits *Olea europaea* cDNA | –2.91 | 0.00 | Unknown |
| fgenesh4_pg.C_scaffold_17737000001 | serine/threonine protein kinase family protein | –2.83 | 0.02 | Protein phosphorylation, transferase activity |
| GO244875_1 | GO244875 OEAA-070810_Plate5n05.b1 cDNA library from Olive leaves and fruits *Olea europaea* cDNA | –2.82 | 0.00 | Unknown |
| GO245033_1 | GO245033 OEAA-070810_Plate6d24.b1 cDNA library from Olive leaves and fruits *Olea europaea* cDNA | –2.82 | 0.01 | Fatty acid metabolic process, oxidation-reduction process |
| GO245373_1 | GO245373 OEAA-070810_Plate7c14.b1 cDNA library from Olive leaves and fruits *Olea europaea* cDNA | –2.80 | 0.00 | Cellular divalent inorganic cation homeostasis, monovalent inorganic cation transport, response to abiotic stimulus, response to chemical stimulus, response to stress, transmembrane transport, calcium ion transport |
| GO245362_1 | GO245362 OEAA-070810_Plate7c03.b1 cDNA library from Olive leaves and fruits *Olea europaea* cDNA | –2.79 | 0.00 | Terpenoid biosynthetic process |
| GO244454_1 | GO244454 OEAA-070810_Plate4k20.b1 cDNA library from Olive leaves and fruits *Olea europaea* cDNA | –2.76 | 0.00 | Unknown |
| FL684040_1 | FL684040 B_A08_A04_0414F_p6 *Olea europaea* cv. Leccino fruitlet *Olea europaea* cDNA | –2.76 | 0.00 | Unknown |
| GO243430_1 | GO243430 OEAA-070810_Plate1o24.b1 cDNA library from Olive leaves and fruits *Olea europaea* cDNA | –2.76 | 0.00 | Unknown |
| GO243145_1 | GO243145 OEAA-070810_Plate1c16.b1 cDNA library from Olive leaves and fruits *Olea europaea* cDNA | –2.72 | 0.00 | Metabolic process |
| FL684259_1 | FL684259 C_F20_C10_0414F_p8 *Olea europaea* cv. Leccino fruitlet *Olea europaea* cDNA | –2.71 | 0.00 | Phosphorylation |
| GO246127_1 | GO246127 OEAA-070810_Plate9c17.b1 cDNA library from Olive leaves and fruits *Olea europaea* cDNA | –2.70 | 0.01 | Unknown |
| FN997779_1 | FN997779 FN997779 *Olea europaea* flower *Olea europaea* cDNA clone c1-2-E11 | –2.64 | 0.00 | Unknown |
| GO244392_1 | GO244392 OEAA-070810_Plate4i03.b1 cDNA library from Olive leaves and fruits *Olea europaea* cDNA | –2.64 | 0.00 | Unknown |
| GO243445_1 | GO243445 OEAA-070810_Plate1p15.b1 cDNA library from Olive leaves and fruits *Olea europaea* cDNA | –2.61 | 0.01 | Cutin biosynthetic process, phosphatidylglycerol biosynthetic process, regulation of meristem growth |
| GO245488_1 | GO245488 OEAA-070810_Plate7h13.b1 cDNA library from Olive leaves and fruits *Olea europaea* cDNA | –2.59 | 0.00 | Defense response to fungus, incompatible interaction, salicylic acid metabolic process, systemic acquired resistance, salicylic acid mediated signalling pathway, salicylic acid metabolic process, systemic acquired resistance, salicylic acid mediated signaling pathway |
| GO244708_1 | GO244708 OEAA-070810_Plate5f21.b1 cDNA library from Olive leaves and fruits *Olea europaea* cDNA | –2.59 | 0.00 | Chlorophyll biosynthetic process |
| GO243140_1 | GO243140 OEAA-070810_Plate1c11.b1 cDNA library from Olive leaves and fruits *Olea europaea* cDNA | –2.58 | 0.00 | Unknown |
| GO244609_1 | GO244609 OEAA-070810_Plate5b13.b1 cDNA library from Olive leaves and fruits *Olea europaea* cDNA | –2.57 | 0.00 | Cutin biosynthetic process, phosphatidylglycerol biosynthetic process, regulation of meristem growth, phosphatidylglycerol biosynthetic process, regulation of meristem growth |
| GO245605_1 | GO245605 OEAA-070810_Plate7m13.b1 cDNA library from Olive leaves and fruits *Olea europaea* cDNA | –2.56 | 0.00 | Metabolic process |
| GO242798_1 | GO242798 OEAA-070810_Plate10d24.b1 cDNA library from Olive leaves and fruits *Olea europaea* cDNA | –2.55 | 0.00 | Carbohydrate metabolic process |
| GO245994_1 | GO245994 OEAA-070810_Plate8n01.b1 cDNA library from Olive leaves and fruits *Olea europaea* cDNA | –2.55 | 0.00 | Jasmonic acid biosynthetic process, protein targeting to vacuole, response to wounding |
| GO243051_1 | GO243051 OEAA-070810_Plate10o15.b1 cDNA library from Olive leaves and fruits *Olea europaea* cDNA | –2.53 | 0.00 | Monovalent inorganic cation transport, response to abiotic stimulus, response to chemical stimulus, response to stress, transmembrane transport, calcium ion transport, cellular divalent inorganic cation homeostasis |
| GO246332_1 | GO246332 OEAA-070810_Plate9l11.b1 cDNA library from Olive leaves and fruits *Olea europaea* cDNA | –2.53 | 0.00 | Unknown |
| GO245255_1 | GO245255 OEAA-070810_Plate6n12.b1 cDNA library from Olive leaves and fruits *Olea europaea* cDNA | –2.50 | 0.00 | Regulation of glucosinolate biosynthetic process |
| GO245983_1 | GO245983 OEAA-070810_Plate8m14.b1 cDNA library from Olive leaves and fruits *Olea europaea* cDNA | –2.50 | 0.00 | Proteolysis |
| GO243038_1 | GO243038 OEAA-070810_Plate10o02.b1 cDNA library from Olive leaves and fruits *Olea europaea* cDNA | –2.49 | 0.00 | Monovalent inorganic cation transport, response to abiotic stimulus, response to chemical stimulus, response to stress, transmembrane transport, calcium ion transport, cellular divalent inorganic cation homeostasis |
| GO243771_1 | GO243771 OEAA-070810_Plate2n24.b1 cDNA library from Olive leaves and fruits *Olea europaea* cDNA | –2.48 | 0.00 | Lipid metabolic process |
| GO245374_1 | GO245374 OEAA-070810_Plate7c15.b1 cDNA library from Olive leaves and fruits *Olea europaea* cDNA | –2.48 | 0.00 | Unknown |
| gw1.558.6.1 | integrase [Populus trichocarpa] | –2.48 | 0.00 | Unknown |
| GO243421_1 | GO243421 OEAA-070810_Plate1o15.b1 cDNA library from Olive leaves and fruits *Olea europaea* cDNA | –2.47 | 0.01 | Defense response, microtubule-based movement, response to biotic stimulus |
| GO245198_1 | GO245198 OEAA-070810_Plate6l01.b1 cDNA library from Olive leaves and fruits *Olea europaea* cDNA | –2.47 | 0.00 | Regulation of cellular process |
| GO244998_1 | GO244998 OEAA-070810_Plate6c12.b1 cDNA library from Olive leaves and fruits *Olea europaea* cDNA | –2.46 | 0.00 | Unknown |
| GO244267_1 | GO244267 OEAA-070810_Plate4c21.b1 cDNA library from Olive leaves and fruits *Olea europaea* cDNA | –2.45 | 0.00 | Unknown |
| gw1.IV.4047.1 | 30S ribosomal protein, putative | –2.45 | 0.01 | Primary metabolic process |
| GO243101_1 | GO243101 OEAA-070810_Plate1a19.b1 cDNA library from Olive leaves and fruits *Olea europaea* cDNA | –2.45 | 0.00 | Microtubule-based movement, regulation of defense response, starch metabolic process, circadian rhythm |
| GO244040_1 | GO244040 OEAA-070810_Plate3j08.b1 cDNA library from Olive leaves and fruits *Olea europaea* cDNA | –2.44 | 0.00 | Unknown |
| eugene3.00140920 | TT4, ATCHS, CHS \| CHS (CHALCONE SYNTHASE) | –2.43 | 0.00 | Auxin polar transport, biosynthetic process, metabolic process, transferase activity |
| GO243685_1 | GO243685 OEAA-070810_Plate2j24.b1 cDNA library from Olive leaves and fruits *Olea europaea* cDNA | –2.42 | 0.01 | Hyperosmotic salinity response, iron ion transport, nitrate transport, oxidation-reduction process, xylem and phloem pattern formation, brassinosteroid biosynthetic process, cellular response to iron ion starvation |
| FL684270_1 | FL684270 A_I17_E09_0414C_p1 *Olea europaea* cv. Leccino fruitlet *Olea europaea* cDNA | –2.42 | 0.00 | Terpenoid biosynthetic process |
| GO244702_1 | GO244702 OEAA-070810_Plate5f15.b1 cDNA library from Olive leaves and fruits *Olea europaea* cDNA | –2.42 | 0.00 | Golgi organization, methylammonium transmembrane transport, response to salt stress, urea transmembrane transport, water transport, calcium ion transport, cellular cation homeostasis |
| GO245584_1 | GO245584 OEAA-070810_Plate7l16.b1 cDNA library from Olive leaves and fruits *Olea europaea* cDNA | –2.40 | 0.00 | Unknown |
| GO245145_1 | GO245145 OEAA-070810_Plate6i18.b1 cDNA library from Olive leaves and fruits *Olea europaea* cDNA | –2.40 | 0.00 | Trichome morphogenesis, cell wall biogenesis |
| gw1.XIX.1830.1 | hydrolase, alpha/beta fold family protein | –2.40 | 0.00 | Unknown |
| GO243632_1 | GO243632 OEAA-070810_Plate2h18.b1 cDNA library from Olive leaves and fruits *Olea europaea* cDNA | –2.39 | 0.00 | Fatty acid oxidation, flower development, pollen tube development, vernalization response, cutin biosynthetic process, double fertilization forming a zygote and endosperm |
| GO246350_1 | GO246350 OEAA-070810_Plate9m08.b1 cDNA library from Olive leaves and fruits *Olea europaea* cDNA | –2.39 | 0.00 | Cell adhesion |
| GO246108_1 | GO246108 OEAA-070810_Plate9b22.b1 cDNA library from Olive leaves and fruits *Olea europaea* cDNA | –2.39 | 0.00 | Monovalent inorganic cation transport, response to abiotic stimulus, response to chemical stimulus, response to stress, transmembrane transport, calcium ion transport, cellular divalent inorganic cation homeostasis |
| GO243337_1 | GO243337 OEAA-070810_Plate1l02.b1 cDNA library from Olive leaves and fruits *Olea europaea* cDNA | –2.38 | 0.02 | Lipid metabolic process, oxidation-reduction process, |
| estExt_fgenesh4_pg.C_LG_V1618 | similar to unknown protein [Arabidopsis thaliana] (TAIR:AT2G37210.1) | –2.38 | 0.01 | Unknown |
| GO243427_1 | GO243427 OEAA-070810_Plate1o21.b1 cDNA library from Olive leaves and fruits *Olea europaea* cDNA | –2.36 | 0.00 | Unknown |
| GO244184_1 | GO244184 OEAA-070810_Plate3p09.b1 cDNA library from Olive leaves and fruits *Olea europaea* cDNA | –2.36 | 0.01 | Unknown |
| GO245561_1 | GO245561 OEAA-070810_Plate7k15.b1 cDNA library from Olive leaves and fruits *Olea europaea* cDNA | –2.35 | 0.00 | Unknown |
| GO243500_1 | GO243500 OEAA-070810_Plate2b24.b1 cDNA library from Olive leaves and fruits *Olea europaea* cDNA | –2.35 | 0.00 | Unknown |
| GO242745_1 | GO242745 OEAA-070810_Plate10b19.b1 cDNA library from Olive leaves and fruits *Olea europaea* cDNA | –2.32 | 0.00 | Monovalent inorganic cation transport, response to abiotic stimulus, response to chemical stimulus, response to stress, transmembrane transport, calcium ion transport, cellular divalent inorganic cation homeostasis |
| eugene3.00110442 | eugene3.00110442: hypothetical protein PFB0460c [Plasmodium falciparum 3D7] | –2.32 | 0.02 | Unknown |
| FL683558_1 | FL683558 A_M17_G09_0414F_p1 *Olea europaea* cv. Leccino fruitlet *Olea europaea* cDNA | –2.32 | 0.00 | Glycolysis, golgi organization, hyperosmotic response, regulation of protein localization, response to cadmium ion, response to salt stress, response to temperature stimulus, response to water deprivation, transmembrane transport, water transport, carbon dioxide transport |
| fgenesh4_pg.C_LG_XIV000630 | DNAJ heat shock N-terminal domain-containing protein | –2.28 | 0.02 | Protein binding |
| GO245281_1 | GO245281 OEAA-070810_Plate6o15.b1 cDNA library from Olive leaves and fruits *Olea europaea* cDNA | –2.27 | 0.00 | Lipid metabolic process |
| GO246184_1 | GO246184 OEAA-070810_Plate9f02.b1 cDNA library from Olive leaves and fruits *Olea europaea* cDNA | –2.24 | 0.01 | Monovalent inorganic cation transport, response to abiotic stimulus, response to chemical stimulus, response to stress, transmembrane transport, calcium ion transport, cellular divalent inorganic cation homeostasis |
| eugene3.00040001 | SKS4 \| SKS4 (SKU5 Similar 4) | –2.22 | 0.00 | Oxidation-reduction process |
| GO245788_1 | GO245788 OEAA-070810_Plate8e07.b1 cDNA library from Olive leaves and fruits *Olea europaea* cDNA | –2.22 | 0.00 | Oxidation-reduction process |
| FN998444_1 | FN998444 FN998444 *Olea europaea* flower *Olea europaea* cDNA clone c2-2-F11 | –2.21 | 0.04 | Carbohydrate metabolic process |
| GO242755_1 | GO242755 OEAA-070810_Plate10c05.b1 cDNA library from Olive leaves and fruits *Olea europaea* cDNA | –2.21 | 0.02 | Transport |
| GO243390_1 | GO243390 OEAA-070810_Plate1n08.b1 cDNA library from Olive leaves and fruits *Olea europaea* cDNA | –2.21 | 0.01 | Methylation |
| GO244398_1 | GO244398 OEAA-070810_Plate4i09.b1 cDNA library from Olive leaves and fruits *Olea europaea* cDNA | –2.19 | 0.00 | Lipid metabolic process |
| GO246009_1 | GO246009 OEAA-070810_Plate8n16.b1 cDNA library from Olive leaves and fruits *Olea europaea* cDNA | –2.19 | 0.01 | Monovalent inorganic cation transport, response to abiotic stimulus, response to chemical stimulus, response to stress, transmembrane transport, calcium ion transport, cellular divalent inorganic cation homeostasis |
| GO246175_1 | GO246175 OEAA-070810_Plate9e17.b1 cDNA library from Olive leaves and fruits *Olea europaea* cDNA | –2.18 | 0.01 | Monovalent inorganic cation transport, response to abiotic stimulus, response to chemical stimulus, response to stress, transmembrane transport, calcium ion transport, cellular divalent inorganic cation homeostasis |
| FL684112_1 | FL684112 A_M13_G07_0414F_p1 *Olea europaea* cv. Leccino fruitlet *Olea europaea* cDNA | –2.18 | 0.00 | Terpenoid biosynthetic process |
| GO245948_1 | GO245948 OEAA-070810_Plate8l02.b1 cDNA library from Olive leaves and fruits *Olea europaea* cDNA | –2.17 | 0.01 | Unknown |
| GO245712_1 | GO245712 OEAA-070810_Plate8b01.b1 cDNA library from Olive leaves and fruits *Olea europaea* cDNA | –2.17 | 0.00 | Monovalent inorganic cation transport, response to abiotic stimulus, calcium ion transport, cellular divalent inorganic cation homeostasis, response to chemical stimulus, response to stres, transmembrane transport |
| GO245518_1 | GO245518 OEAA-070810_Plate7i20.b1 cDNA library from Olive leaves and fruits *Olea europaea* cDNA | –2.15 | 0.00 | Regulation of secondary cell wall biogenesis, regulation of transcription, DNA-dependent, response to abscisic acid stimulus |
| estExt_fgenesh4_pg.C_LG_XII1027 | ICE1 \| ICE1 (INDUCER OF CBF EXPRESSION 1) | –2.15 | 0.01 | Transcription regulator activity, regulation of cellular transcription |
| GO243925_1 | GO243925 OEAA-070810_Plate3e12.b1 cDNA library from Olive leaves and fruits *Olea europaea* cDNA | –2.15 | 0.00 | Unknown |
| GO244028_1 | GO244028 OEAA-070810_Plate3i20.b1 cDNA library from Olive leaves and fruits *Olea europaea* cDNA | –2.15 | 0.00 | Oxidation-reduction process, fatty acid biosynthetic process |
| GO244729_1 | GO244729 OEAA-070810_Plate5g22.b1 cDNA library from Olive leaves and fruits *Olea europaea* cDNA | –2.15 | 0.00 | Trichome morphogenesis, cell wall biogenesis, cysteine biosynthetic process |
| GO242772_1 | GO242772 OEAA-070810_Plate10c22.b1 cDNA library from Olive leaves and fruits *Olea europaea* cDNA | –2.14 | 0.00 | Monovalent inorganic cation transport, response to abiotic stimulus, response to chemical stimulus, response to stress, calcium ion transport, cellular divalent inorganic cation homeostasis, transmembrane transport |
| GO246093_1 | GO246093 OEAA-070810_Plate9b07.b1 cDNA library from Olive leaves and fruits *Olea europaea* cDNA | –2.11 | 0.00 | Monovalent inorganic cation transport, response to abiotic stimulus, response to chemical stimulus, response to stress, transmembrane transport, calcium ion transport, cellular divalent inorganic cation homeostasis |
| GO242993_1 | GO242993 OEAA-070810_Plate10m05.b1 cDNA library from Olive leaves and fruits *Olea europaea* cDNA | –2.11 | 0.01 | Monovalent inorganic cation transport, response to abiotic stimulus, response to chemical stimulus, response to stress, transmembrane transport, calcium ion transport, cellular divalent inorganic cation homeostasis, |
| GO244140_1 | GO244140 OEAA-070810_Plate3n12.b1 cDNA library from Olive leaves and fruits *Olea europaea* cDNA | –2.11 | 0.01 | Oxidation-reduction process, fatty acid metabolic process |
| GO243304_1 | GO243304 OEAA-070810_Plate1j17.b1 cDNA library from Olive leaves and fruits *Olea europaea* cDNA | –2.11 | 0.00 | Unknown |
| GO245646_1 | GO245646 OEAA-070810_Plate7o06.b1 cDNA library from Olive leaves and fruits *Olea europaea* cDNA | –2.10 | 0.00 | Unknown |
| GO246394_1 | GO246394 OEAA-070810_Plate9o05.b1 cDNA library from Olive leaves and fruits *Olea europaea* cDNA | –2.10 | 0.01 | Monovalent inorganic cation transport, response to abiotic stimulus, response to chemical stimulus, response to stres, transmembrane transport, calcium ion transport, cellular divalent inorganic cation homeostasis |
| FL684145_1 | FL684145 C_I19_E10_0414F_p9 *Olea europaea* cv. Leccino fruitlet *Olea europaea* cDNA | –2.10 | 0.01 | Phosphorylation, response to cadmium ion, glycolysis, lipid metabolic process |
| FN998690_1 | FN998690 FN998690 *Olea europaea* flower *Olea europaea* cDNA clone c2-6-D1 | –2.10 | 0.01 | Oxidation-reduction process, proteolysis, response to ethylene stimulus, aging, defense response to fungus, incompatible interaction |
| gw1.XIX.158.1 | disease resistance protein (CC-NBS-LRR class), putative | –2.10 | 0.00 | Defense response |
| GO245314_1 | GO245314 OEAA-070810_Plate7a02.b1 cDNA library from Olive leaves and fruits *Olea europaea* cDNA | –2.09 | 0.00 | Unknown |
| GO242964_1 | GO242964 OEAA-070810_Plate10k23.b1 cDNA library from Olive leaves and fruits *Olea europaea* cDNA | –2.09 | 0.05 | Monovalent inorganic cation transport, response to abiotic stimulus, response to chemical stimulus, response to stress, transmembrane transport, calcium ion transport, cellular divalent inorganic cation homeostasis |
| GO246036_1 | GO246036 OEAA-070810_Plate8o21.b1 cDNA library from Olive leaves and fruits *Olea europaea* cDNA | –2.08 | 0.00 | Salicylic acid metabolic process |
| GO245065_1 | GO245065 OEAA-070810_Plate6f08.b1 cDNA library from Olive leaves and fruits *Olea europaea* cDNA | –2.08 | 0.00 | Oxidation-reduction process |
| GO245725_1 | GO245725 OEAA-070810_Plate8b16.b1 cDNA library from Olive leaves and fruits *Olea europaea* cDNA | –2.08 | 0.00 | Response to abiotic stimulus, response to chemical stimulus, response to stress, transmembrane transport, calcium ion transport, cellular divalent inorganic cation homeostasis, monovalent inorganic cation transport |
| GO244529_1 | GO244529 OEAA-070810_Plate4o02.b1 cDNA library from Olive leaves and fruits *Olea europaea* cDNA | –2.08 | 0.00 | Unknown |
| GO244411_1 | GO244411 OEAA-070810_Plate4i22.b1 cDNA library from Olive leaves and fruits *Olea europaea* cDNA | –2.08 | 0.01 | Unknown |
| GO245517_1 | GO245517 OEAA-070810_Plate7i19.b1 cDNA library from Olive leaves and fruits *Olea europaea* cDNA | –2.08 | 0.01 | Regulation of secondary cell wall biogenesis, regulation of transcription, DNA-dependent, response to abscisic acid stimulus |
| GO245986_1 | GO245986 OEAA-070810_Plate8m17.b1 cDNA library from Olive leaves and fruits *Olea europaea* cDNA | –2.07 | 0.01 | Response to abiotic stimulus, response to chemical stimulus, response to stress, transmembrane transport, calcium ion transport, cellular divalent inorganic cation homeostasis, monovalent inorganic cation transport |
| FN998032_1 | FN998032 FN998032 *Olea europaea* flower *Olea europaea* cDNA clone c1-5-D6 | –2.06 | 0.00 | Unknown |
| GO245840_1 | GO245840 OEAA-070810_Plate8g11.b1 cDNA library from Olive leaves and fruits *Olea europaea* cDNA | –2.06 | 0.01 | Unknown |
| FL683532_1 | FL683532 B_A20_A10_0414C_p2 *Olea europaea* cv. Leccino fruitlet *Olea europaea* cDNA | –2.06 | 0.01 | Unknown |
| GO242886_1 | GO242886 OEAA-070810_Plate10h17.b1 cDNA library from Olive leaves and fruits *Olea europaea* cDNA | –2.05 | 0.00 | Response to abiotic stimulus, response to chemical stimulus, response to stress, transmembrane transport, calcium ion transport, cellular divalent inorganic cation homeostasis, monovalent inorganic cation transport |
| eugene3.02140018 | HhH-GPD base excision DNA repair family protein | –2.04 | 0.05 | Base-excision repair, DNA repair |
| GO244546_1 | GO244546 OEAA-070810_Plate4o19.b1 cDNA library from Olive leaves and fruits *Olea europaea* cDNA | –2.04 | 0.01 | Pentose-phosphate shunt, protein phosphorylation, signal transduction, valine metabolic process |
| GO243107_1 | GO243107 OEAA-070810_Plate1b01.b1 cDNA library from Olive leaves and fruits *Olea europaea* cDNA | –2.04 | 0.00 | Oxidation-reduction process, abscisic acid mediated signalling pathway, cellular response to water deprivation, response to hydrogen peroxide, toxin catabolic process |
| GO245678_1 | GO245678 OEAA-070810_Plate7p14.b1 cDNA library from Olive leaves and fruits *Olea europaea* cDNA | –2.04 | 0.00 | Oxidation-reduction process, response to oxidative stress, response to salt stres, trichoblast differentiation, |
| GO245514_1 | GO245514 OEAA-070810_Plate7i16.b1 cDNA library from Olive leaves and fruits *Olea europaea* cDNA | –2.04 | 0.01 | Unknown |
| GO244958_1 | GO244958 OEAA-070810_Plate6a19.b1 cDNA library from Olive leaves and fruits *Olea europaea* cDNA | –2.04 | 0.00 | Unknown |
| GO244126_1 | GO244126 OEAA-070810_Plate3m22.b1 cDNA library from Olive leaves and fruits *Olea europaea* cDNA | –2.03 | 0.00 | Photosynthetic electron transport chain, response to karrikin, response to light stimulus, ferredoxin metabolic process |
| eugene3.00051033 | RPS18C \| RPS18C (S18 RIBOSOMAL PROTEIN) | –2.03 | 0.00 | Translation |
| GO244048_1 | GO244048 OEAA-070810_Plate3j16.b1 cDNA library from Olive leaves and fruits *Olea europaea* cDNA | –2.03 | 0.00 | Protein folding, response to arsenic-containing substance, response to heat, response to high light intensity, response to hydrogen peroxide, defense response to bacterium, incompatible interaction |
| GO245177_1 | GO245177 OEAA-070810_Plate6k02.b1 cDNA library from Olive leaves and fruits *Olea europaea* cDNA | –2.01 | 0.01 | Unknown |
| CK087201_1 | CK087201 Olest08 Differential Display olive fragments *Olea europaea* cDNA | –2.01 | 0.00 | Unknown |
| gw1.1883.5.1 | protein kinase family protein | –2.01 | 0.00 | Protein phosphorylation, transferase activity |
| GO246079_1 | GO246079 OEAA-070810_Plate9a17.b1 cDNA library from Olive leaves and fruits *Olea europaea* cDNA | –2.01 | 0.00 | Response to abiotic stimulus, response to chemical stimulus, response to stress, transmembrane transport, calcium ion transport, cellular divalent inorganic cation homeostasis, monovalent inorganic cation transport |
| FN998199_1 | FN998199 FN998199 *Olea europaea* flower *Olea europaea* cDNA clone c1-7-C11 | –2.00 | 0.01 | Unknown |
| gw1.II.3432.1 | MYB55 (myb domain protein 55) | –2.00 | 0.00 | Regulation of transcription |
| GO243189_1 | gi\|242395302\|gb\|GO243189.1\|GO243189 OEAA-070810_Plate1e16.b1 cDNA library from Olive leaves and fruits *Olea europaea* cDNA, mRNA sequence | –2.00 | 0.01 | Embryo development ending in seed dormancy, cutin biosynthetic process, epidermis morphogenesis, localization, trichome morphogenesis |
| fgenesh4_pg.C_scaffold_281000008 | fgenesh4_pg.C_scaffold_281000008: hypothetical protein [Arabidopsis thaliana] | 2.00 | 0.00 | Unknown |
| eugene3.00140454 | similar to fiber protein Fb11 [Gossypium barbadense] (GB:AAN77150.1) | 2.01 | 0.00 | Unknown |
| fgenesh4_pg.C_scaffold_1315000001 | Identical to SGT1 protein homolog At5g65490 [Arabidopsis Thaliana] | 2.02 | 0.00 | Unknown |
| fgenesh1_pg.C_LG_XVIII001240 | fgenesh1_pg.C_LG_XVIII001240: unknown protein [Oryza sativa (japonica cultivar-group)] | 2.03 | 0.01 | Unknown |
| eugene3.00060638 | myosin heavy chain-related | 2.03 | 0.00 | D-xylose metabolic process, glucose catabolic process |
| fgenesh4_pg.C_scaffold_5944000001 | SPX (SYG1/Pho81/XPR1) domain-containing protein | 2.05 | 0.00 | Cellular response to phosphate starvation, galactolipid biosynthetic process, negative regulation of transcription, phosphate ion transport |
| eugene3.36010001 | extracellular dermal glycoprotein, putative / EDGP, putative | 2.05 | 0.00 | Proteolysis, response to salt stress |
| gw1.40.142.1 | ATJ, ATJ3 \| ATJ3 (Arabidopsis thaliana DnaJ homologue 3) | 2.10 | 0.00 | N-terminal protein myristoylation, photoperiodism, positive regulation of flower development, protein folding, protein folding, regulation of ATPase activity, protein folding, response to salt stress |
| estExt_fgenesh4_pg.C_LG_VIII1198 | similar to Os03g0713200 [Oryza sativa (japonica cultivar-group)] | 2.11 | 0.00 | Unknown |
| eugene3.00161257 | eugene3.00161257: hypothetical protein glr0432 [Gloeobacter violaceus PCC 7421] | 2.11 | 0.00 | Unknown |
| eugene3.01420101 | DNA cross-link repair protein-related | 2.12 | 0.00 | Unknown |
| fgenesh4_pg.C_scaffold_3342000001 | fgenesh4_pg.C_scaffold_3342000001: 60I2G03 [Populus deltoides] | 2.13 | 0.00 | Unknown |
| fgenesh4_pg.C_scaffold_4111000001 | fgenesh4_pg.C_scaffold_4111000001: Hypothetical protein F07B10.4 [Caenorhabditis elegans] | 2.14 | 0.00 | Unknown |
| eugene3.02080002 | similar to unnamed protein product [Ostreococcus tauri] (GB:CAL54676.1) | 2.14 | 0.02 | Carotenoid biosynthetic process, cell differentiation, leaf morphogenesis, pentose-phosphate shunt, thylakoid membrane organization |
| GO242816_1 | GO242816 OEAA-070810_Plate10e18.b1 cDNA library from Olive leaves and fruits *Olea europaea* cDNA | 2.18 | 0.03 | Response to abiotic stimulus, response to chemical stimulus, response to stress, transmembrane transport, calcium ion transport, cellular divalent inorganic cation homeostasis, monovalent inorganic cation transport |
| fgenesh4_pg.C_LG_I000158 | fgenesh4_pg.C_LG_I000158: F14J16.29 [Arabidopsis thaliana] | 2.18 | 0.00 | Unknown |
| fgenesh4_pg.C_LG_XII001090 | fgenesh4_pg.C_LG_XII001090: B1096D03.4 [Oryza sativa (japonica cultivar-group)] | 2.20 | 0.00 | Unknown |
| estExt_fgenesh4_pm.C_LG_I0430 | SNF7 family protein | 2.22 | 0.00 | Protein transport |
| fgenesh1_pg.C_scaffold_97000024 | hypothetical protein [Vitis vinifera] | 2.23 | 0.00 | Unknown |
| eugene3.49050001 | eugene3.49050001: unnamed protein product [Rattus rattus] | 2.24 | 0.03 | Unknown |
| gw1.212.39.1 | disease resistance protein (TIR-NBS-LRR class), putative | 2.26 | 0.01 | Defense response, signal transduction |
| fgenesh4_pg.C_scaffold_19987000001 | LOX3 \| LOX3 (Lipoxygenase 3) | 2.28 | 0.00 | Anther dehiscence, anther development, defense response, ethylene biosynthetic process, growth, response to chitin, response to fungus, response to high light intensity, response to jasmonic acid stimulus, response to wounding, stamen filament development |
| estExt_fgenesh4_pg.C_LG_XI0828 | IQD9 \| IQD9 (IQ-domain 9) | 2.28 | 0.00 | Methionine biosynthetic process, photoperiodism, RNA splicing |
| gw1.I.6534.1 | PSY \| PSY (PHYTOENE SYNTHASE) | 2.29 | 0.00 | Biosynthetic process, transferase activity |
| fgenesh4_pg.C_LG_VIII000311 | fgenesh4_pg.C_LG_VIII000311: hypothetical protein XP_397607 [Apis mellifera] | 2.29 | 0.01 | Unknown |
| eugene3.25290001 |  | 2.32 | 0.04 | Unknown |
| gw1.IX.1138.1 | HPR (Hydroxypyruvate reductase) | 2.32 | 0.02 | Metabolic process, oxidation-reduction process, oxidoreductase activity |
| fgenesh4_pg.C_scaffold_29000280 | fgenesh4_pg.C_scaffold_29000280: hypothetical protein PB001304.00.0 [Plasmodium berghei] | 2.33 | 0.00 | Unknown |
| eugene3.00110978 | ULT2 \| ULT2 (ULTRAPETALA 2) | 2.34 | 0.01 | Unknown |
| fgenesh4_pg.C_LG_VI001586 | fgenesh4_pg.C_LG_VI001586: NAM (no apical meristem)-like protein [Arabidopsis thaliana] | 2.36 | 0.01 | Unknown |
| fgenesh4_pg.C_LG_X001297 | BG1 \| BG1 (BETA-1,3-GLUCANASE 1) | 2.37 | 0.01 | Carbohydrate metabolic process, hydrolase activity |
| fgenesh4_pg.C_scaffold_274000003 | leucine-rich repeat family protein / protein kinase family protein | 2.37 | 0.01 | Protein phosphorylation, transferase activity |
| estExt_fgenesh4_pg.C_LG_VI1748 | proteasome maturation factor UMP1 family protein | 2.41 | 0.00 | Unknown |
| grail3.2490000101 | extracellular dermal glycoprotein, putative / EDGP, putative | 2.42 | 0.00 | Proteolysis, response to salt stres |
| eugene3.128660001 | AMP-dependent synthetase and ligase family protein | 2.48 | 0.00 | Metabolic process |
| eugene3.00011108 | eugene3.00011108: putative aldo/keto reductase [Oryza sativa (japonica cultivar-group)] | 2.49 | 0.00 | Unknown |
| estExt_Genewise1_v1.C_7600034 | hypothetical protein NitaMp079 [Nicotiana tabacum] | 2.50 | 0.00 | Unknown |
| fgenesh4_pg.C_LG_VI000379 | similar to Os11g0140100 [Oryza sativa (japonica cultivar-group)] | 2.55 | 0.00 | Unknown |
| eugene3.00102522 | 60S acidic ribosomal protein P2 (RPP2A) | 2.60 | 0.01 | Translational elongation |
| eugene3.00160596 | RHF2A, CIC7E11 \| CIC7E11 | 2.63 | 0.02 | Autophagy, cellular macromolecule catabolic process, fatty acid beta-oxidation, hormone-mediated signaling pathway, mega gametogenesis, micro gametogenesis, protein import into peroxisome matrix, proteolysis involved in cellular protein catabolic process, regulation of cell cycle, salicylic acid biosynthetic process, signal transduction, systemic acquired resistance |
| fgenesh4_pg.C_LG_II000240 | fgenesh4_pg.C_LG_II000240: regulator of chromosome condensation (RCC1) family protein [Arabidopsis thaliana] | 2.65 | 0.00 | Unknown |
| gw1.VII.1831.1 | PED3, CTS, PXA1 \| PXA1 (PEROXISOMAL ABC TRANSPORTER 1) | 2.68 | 0.01 | Transport, transmembrane transport, ATPase activity |
| gw1.XIV.3648.1 | Mo25 family protein | 2.68 | 0.01 | Unknown |
| gw1.XIII.74.1 | S-locus lectin protein kinase family protein | 2.80 | 0.00 | Recognition of polen, protein phosphorylation, transferase activity |
| gw1.VI.1127.1 | kinesin motor protein-related | 2.94 | 0.01 | DNA repair, microtubule-based movement |
| grail3.7095000201 | grail3.7095000201: putative salivary protein [Culicoides sonorensis] | 3.03 | 0.00 | Unknown |
| gw1.I.375.1 | protein kinase family protein | 3.15 | 0.00 | Protein phosphorylation, transferase activity |
| eugene3.00110007 | eugene3.00110007: putative taxane 14b-hydroxylase [Oryza sativa (japonica cultivar-group)] | 3.30 | 0.01 | Unknown |
| gw1.168.103.1 | CYP72A11 \| CYP72A11 (cytochrome P450, family 72, subfamily A, polypeptide 11) | 3.38 | 0.00 | Oxidation-reduction process, oxidoreductase activity |
| eugene3.00020895 | protein phosphatase 2C, putative / PP2C, putative | 3.41 | 0.00 | Cellular membrane fusion, ethylene biosynthetic process, protein dephosphorylation, red light signaling pathway, response to molecule of bacterial origin |
| fgenesh4_pg.C_LG_XIV001375 | similar to unknown protein [Arabidopsis thaliana] (TAIR:AT2G31840.1) | 3.42 | 0.01 | Chloroplast organization, regulation of transcription |
| fgenesh1_pg.C_scaffold_3865000001 | fgenesh1_pg.C_scaffold_3865000001: hypothetical protein [Oryza sativa (japonica cultivar-group)] | 3.50 | 0.00 | Unknown |
| GO244009_1 | GO244009 OEAA-070810_Plate3i01.b1 cDNA library from Olive leaves and fruits *Olea europaea* cDNA | 3.58 | 0.00 | Oxidation-reduction process, photorespiration, reductive pentose-phosphate cycle, |
| grail3.0039017801 | AP2 domain-containing transcription factor, putative | 3.61 | 0.00 | Regulation of transcription |
| gw1.181.32.1 | similar to unknown protein [Arabidopsis thaliana] (TAIR:AT3G15310.1) | 3.71 | 0.02 | Unknown |
| eugene3.00170500 | similar to unknown protein [Arabidopsis thaliana] (TAIR:AT1G13740.1) | 3.80 | 0.00 | Response to abscisic acid stimulus |
| eugene3.07630001 | NAP1 | 3.85 | 0.00 | Nucleosome assembly |
| fgenesh4_pg.C_scaffold_40000175 | methyltransferase MT-A70 family protein | 3.90 | 0.00 | Methylation, nucleobase-containing compound metabolic process, other cellular processes |
| fgenesh4_pg.C_scaffold_5695000001 | fgenesh4_pg.C_scaffold_5695000001: RAS-related protein racD | 3.93 | 0.01 | Unknown |
| eugene3.00290281 | UGT73B2 \| UGT73B2 | 4.24 | 0.01 | Metabolic process, transferase activity |
| estExt_Genewise1_v1.C_660290 | ATBPM4 \| ATBPM4 (BTB-POZ AND MATH DOMAIN 4) | 4.44 | 0.01 | Protein binding |
| eugene3.00170228 | eugene3.00170228: unnamed protein product [Arabidopsis thaliana] | 4.56 | 0.00 | Unknown |
| estExt_Genewise1_v1.C_LG_XII0166 | ATPase | 4.59 | 0.00 | ATP binding |
| eugene3.00410220 | histidyl-tRNA synthetase, putative / histidine--tRNA ligase, putative | 4.67 | 0.00 | Histidyl-tRNA aminoacylation, tRNA aminoacylation for protein translation, other metabolic processes |
| gw1.614.2.1 | gw1.614.2.1: hypothetical protein [Vitis vinifera] | 4.68 | 0.01 | Unknown |
| eugene3.86990001 | eugene3.86990001: putative non-LTR retroelement reverse transcriptase [Arabidopsis thaliana] | 4.73 | 0.00 | Unknown |
| gw1.123.49.1 | COL2 \| COL2 (CONSTANS-LIKE 2) | 4.76 | 0.00 | Unknown |
| fgenesh4_pm.C_LG_IV000224 | leucine-rich repeat transmembrane protein kinase, putative | 4.85 | 0.00 | Protein phosphorylation, transferase activity |
| eugene3.01250091 | SNF2 domain-containing protein / helicase domain-containing protein / zinc finger (C3HC4 type RING finger) family protein | 5.02 | 0.00 | ATP binding, DNA binding, helicase activity |
| fgenesh4_pg.C_LG_XVII000057 | RAB GDP-dissociation inhibitor | 5.11 | 0.00 | Protein transport |
| eugene3.02430009 | eugene3.02430009: hypothetical protein Bcep02001879 [Burkholderia fungorum LB400] | 6.60 | 0.00 | Unknown |
| **Seq_ID** | **Description** | **ON-J /OFF-J-fold change** | ***P* value** | **GO biological process** |
| grail3.7874000101 | grail3.7874000101: Ig epsilon chain C region | –2.95 | 0.01 | Unknown |
| fgenesh4_pg.C_LG_V000279 | fgenesh4_pg.C_LG_V000279: ABC TRANSPORT SYSTEM ATP-BINDING PROTEIN | –2.64 | 0.01 | Unknown |
| gw1.9592.1.1 | CAS1 (CYCLOARTENOL SYNTHASE 1) | –2.51 | 0.04 | Chlorophyll biosynthetic process, isopentenyl diphosphate biosynthetic process, mevalonate-independent pathway, unsaturated fatty acid biosynthetic process, pentacyclic triterpenoid biosynthetic process, carotenoid biosynthetic process, thylakoid membrane |
| gw1.VII.2355.1 | SAG12 (SENESCENCE-ASSOCIATED GENE 12) | –2.5 | 0.02 | Aging, response to ethylene stimulus, leaf senescence, proteolysis, defense response to fungus, incompatible interaction |
| grail3.0005062601 | transmembrane protein | –2.34 | 0.01 | Citrate transport, transmembrane transport |
| eugene3.22740001 | surfeit locus protein 5 family protein | –2.1 | 0.03 | Regulation of transcription from RNA polymerase II promoter |
| fgenesh4_pg.C_LG_III000148 | fgenesh4_pg.C_LG_III000148: probable cadmium-transporting ATPase | –2.06 | 0.04 | Unknown |
| gw1.II.1879.1 | pfkB-type carbohydrate kinase family protein | –2.01 | 0 | D-ribose catabolic process |
| GO244336_1 | GO244336 OEAA-070810_Plate4f18.b1 cDNA library from Olive leaves and fruits *Olea europaea* cDNA | 2.03 | 0.04 | Unknown |
| FL683585_1 | FL683585 D_K14_F07_0414F_p14 *Olea europaea* cv. Leccino fruitlet *Olea europaea* cDNA | 2.08 | 0.04 | Response to jasmonic acid stimulus, oxidation-reduction process, response to fungus, response to wounding, jasmonic acid biosynthetic process |
| GO243434_1 | GO243434 OEAA-070810_Plate1p04.b1 cDNA library from Olive leaves and fruits *Olea europaea* cDNA | 2.12 | 0.03 | Unknown |
| FL684126_1 | FL684126 D_B23_A12_0414F_p11 *Olea europaea* cv. Leccino fruitlet *Olea europaea* cDNA | 2.13 | 0.04 | Flavonoid biosynthetic process |
| estExt_fgenesh4_pm.C_1570006 | similar to unknown protein [Arabidopsis thaliana] (TAIR:AT1G52330.1) | 2.16 | 0.03 | Response to desiccation |
| GO243532_1 | GO243532 OEAA-070810_Plate2d10.b1 cDNA library from Olive leaves and fruits *Olea europaea* cDNA | 2.35 | 0.04 | Unknown |
| fgenesh4_pg.C_scaffold_140000084 | FAD-binding domain-containing protein | 2.42 | 0.04 | Oxidoreductase activity |
| FL683540_1 | FL683540 C_E05_C03_0414F_p9 *Olea europaea* cv. Leccino fruitlet *Olea europaea* cDNA | 3.19 | 0.04 | Cellulose biosynthetic process, cellular cell wall organization |
