# Supplementary material for: Nutrition Metabolism Plays an Important Role in the Alternate Bearing of the Olive Tree (Olea europaea L.)
Source: PLoS One. 2013 Mar 28;8(3):e59876. doi: 10.1371/journal.pone.0059876 (PMC3610735; doi:10.1371/journal.pone.0059876)
Supplement: Table S5 — The raw qRT-PCR data. (DOCX) [file pone.0059876.s005.docx]

**Table S5.** Raw data of qRT-PCR.

|  | **18S** | | **GO245535** | | **GO243651** | | **GO245913** | | **GO245304** | | **GO244140** | | **FL684126** | | **GO243710** | | **GO244999** | | **FL684399** | |
| --- | --- | --- | --- | --- | --- | --- | --- | --- | --- | --- | --- | --- | --- | --- | --- | --- | --- | --- | --- | --- |
| **Samples** | **C_T_** | **Average** | **C_T_** | **Average** | **C_T_** | **Average** | **C_T_** | **Average** | **C_T_** | **Average** | **C_T_** | **Average** | **C_T_** | **Average** | **C_T_** | **Average** | **C_T_** | **Average** | **C_T_** | **Average** |
| UF | 24.9 | 24.8 | 29.6 | 29.4 | 24.0 | 23.7 | 33.9 | 33.7 | 23.3 | 23.8 | 23.5 | 23.7 | 31.1 | 31.0 | 33.6 | 32.0 | 26.8 | 27.2 | 33.7 | 33.3 |
| UF | 24.8 |  | 29.3 |  | 23.9 |  | 32.8 |  | 24.0 |  | 23.5 |  | 30.6 |  | 31.7 |  | 27.2 |  | 33.3 |  |
| UF | 24.8 |  | 29.3 |  | 23.3 |  | 34.3 |  | 24.2 |  | 24.1 |  | 31.4 |  | 30.6 |  | 27.5 |  | 32.9 |  |
| OM | 24.8 | 24.7 | 32.5 | 33.1 | 22.7 | 22.9 | 39.6 | 38.1 | 31.7 | 31.7 | 29.9 | 30.0 | 21.2 | 21.6 | 35.6 | 34.3 | 27.2 | 26.9 | 25.0 | 24.7 |
| OM | 24.7 |  | 33.3 |  | 22.8 |  | 38.6 |  | 31.8 |  | 29.9 |  | 21.9 |  | 33.6 |  | 26.8 |  | 24.7 |  |
| OM | 24.7 |  | 33.6 |  | 23.3 |  | 36.1 |  | 31.7 |  | 30.3 |  | 21.6 |  | 33.8 |  | 26.6 |  | 24.6 |  |
| ON-M | 24.6 | 24.6 | 24.5 | 24.7 | 21.7 | 22.4 | 34.0 | 33.9 | 33.0 | 33.1 | 33.4 | 33.4 | 30.5 | 30.8 | 33.4 | 33.4 | 26.7 | 25.8 | 35.3 | 35.3 |
| ON-M | 24.6 |  | 24.9 |  | 22.6 |  | 33.7 |  | 32.9 |  | 33.0 |  | 31.0 |  | 33.2 |  | 26.7 |  | 34.5 |  |
| ON-M | 24.4 |  | 24.8 |  | 23.0 |  | 33.9 |  | 33.3 |  | 33.9 |  | 30.8 |  | 33.5 |  | 23.9 |  | 35.9 |  |
| OFF-M | 24.3 | 24.1 | 23.6 | 23.9 | 22.1 | 22.5 | 31.6 | 31.9 | 31.8 | 31.9 | 32.3 | 32.7 | 27.7 | 27.9 | 32.8 | 32.9 | 23.0 | 23.1 | 36.6 | 35.9 |
| OFF-M | 24.1 |  | 24.0 |  | 22.4 |  | 31.9 |  | 31.7 |  | 32.3 |  | 27.8 |  | 32.7 |  | 23.0 |  | 34.8 |  |
| OFF-M | 24.0 |  | 24.2 |  | 23.1 |  | 32.2 |  | 32.1 |  | 33.5 |  | 28.1 |  | 33.3 |  | 23.2 |  | 36.2 |  |
| ON-J | 24.0 | 23.8 | 31.3 | 31.8 | 27.0 | 26.9 | 35.7 | 36.0 | 31.1 | 31.3 | 31.3 | 31.5 | 34.9 | 34.6 | 32.6 | 32.8 | 30.9 | 30.6 | 38.6 | 37.3 |
| ON-J | 23.8 |  | 31.9 |  | 26.8 |  | 35.9 |  | 31.8 |  | 31.2 |  | 35.1 |  | 32.9 |  | 30.3 |  | 34.9 |  |
| ON-J | 23.7 |  | 32.2 |  | 27.0 |  | 36.3 |  | 31.2 |  | 31.9 |  | 34.0 |  | 33.0 |  | 30.6 |  | 38.3 |  |
| OFF-J | 23.6 | 23.6 | 30.5 | 30.6 | 26.4 | 26.5 | 35.5 | 35.2 | 31.7 | 31.9 | 29.8 | 30.0 | 31.3 | 31.3 | 33.9 | 33.6 | 29.7 | 29.5 | 35.3 | 34.3 |
| OFF-J | 23.5 |  | 30.6 |  | 26.3 |  | 35.5 |  | 31.9 |  | 30.1 |  | 31.2 |  | 32.6 |  | 29.8 |  | 33.9 |  |
| OFF-J | 23.5 |  | 30.8 |  | 26.7 |  | 34.5 |  | 32.1 |  | 30.2 |  | 31.3 |  | 34.1 |  | 29.0 |  | 33.6 |  |
